# Transcriptome Analysis Reveals a Gene Expression Pattern That Contributes to Sugarcane Bud Propagation Induced by Indole-3-Butyric Acid

**DOI:** 10.3389/fpls.2022.852886

**Published:** 2022-03-17

**Authors:** Lin Xu, Zhi-Nian Deng, Kai-Chao Wu, Mukesh Kumar Malviya, Manoj Kumar Solanki, Krishan K. Verma, Tian Pang, Yi-Jie Li, Xiao-Yan Liu, Brijendra Kumar Kashyap, Eldessoky S. Dessoky, Wei-Zan Wang, Hai-Rong Huang

**Affiliations:** ^1^Key Laboratory of Sugarcane Biotechnology and Genetic Improvement (Guangxi), Ministry of Agriculture and Rural Area, Sugarcane Research Center, Chinese Academy of Agricultural Sciences, Guangxi Key Laboratory of Sugarcane Genetic Improvement, Sugarcane Research Institute, Guangxi Academy of Agricultural Sciences, Nanning, China; ^2^Plant Cytogenetics and Molecular Biology Group, Institute of Biology, Biotechnology and Environmental Protection, Faculty of Natural Sciences, University of Silesia in Katowice, Katowice, Poland; ^3^Department of Biotechnology Engineering, Institute of Engineering and Technology, Bundelkhand University, Jhansi, India; ^4^Department of Plant Genetic Transformation, Agriculture Genetic Engineering Research Institute, Agriculture Research Center, Giza, Egypt; ^5^Department of Biology, College of Science, Taif University, Taif, Saudi Arabia

**Keywords:** IBA, single-bud seed, sugarcane, root, transcriptome

## Abstract

Sugarcane is a cash crop that plays an integral part in the sugar industry. The Sustainable Sugarcane Initiative (SSI) has been adopted globally, ensuring enough and aiming for more yield, helping increase disease-free sugarcane cultivation. Single-bud seeds could be the best approach for sugarcane cultivation. Indole-3-butyric acid (IBA) is a rooting agent utilized significantly in seedling propagation. Greenhouse experiment results discovered the significant growth promotion in sugarcane seedlings and accumulation of plant hormones at 100 ppm IBA. Next, we performed transcriptomic analysis of sugarcane buds using RNA sequencing and compared their gene expression during root development due to affect of IBA (100 ppm). A total of 113,475 unigenes were annotated with an average length of 836 bp (N50 = 1,536). The comparative RNA-seq study between the control (CK) and IBA-treated (T) buds showed significant differentially expressed unigenes (494 upregulated and 2086 downregulated). The IBA influenced major biological processes including metabolic process, the cellular process, and single-organism process. For cellular component category, cell, cell part, organelle, membrane, and organelle part were mainly affected. In addition, catalytic activity and binding were primarily affected in the molecular function categories. Furthermore, the expression of genes related to plant hormones and signaling pathways was analyzed by qRT-PCR, which was consistent with the RNA-seq expression profile. This study provides new insights into the IBA response to the bud sprouting in sugarcane based on RNA sequencing, and generated information could help further research on breeding improvement of sugarcane.

## Introduction

Sugarcane (*Saccharum officinarum*) is a globally grown commercial crop that belongs to the family Gramineae and is grown extensively all over China, which is the third-largest sugarcane producer in the world after Brazil and India with a production of around 11.6 MT of white sugar (Zhang and Govindaraju, 2018). Guangxi province contributes the highest (7.21 MT), which is one of the main sugarcane-growing areas in China other than Guangdong and Yunnan (Chen et al., 2021). The Chinese sugar industry played a significant part in several bio-product processing units like ethanol, energy (biomass power, bioethanol), yeast production, paper, chemicals, cane juice, and other associated byproducts (Lu and yang, 2019; Khaire et al., 2021). The sugar industry has a significant annual output that makes it very important for the Chinese economy, and it contributes significantly to the development of rural infrastructures like roads, education, medicine, and other facilities, consecutively playing a vital role in the livelihood of many sugarcane farmers and workers directly employed in sugar mills.

Sugarcane productivity is influenced by seed quality, land treatment, monoculture cropping system, fertilization timing, climate change, etc. Still, the most crucial factor is the planting distance, which decides the volume of nutrients absorbed by the plants (Singh et al., 2019). The investment in the area of the sugarcane plantation is vast, and since the economy rests on it, sometimes the conventional methods might incur a loss in its production. This problem can be handled by adopting the Sustainable Sugarcane Initiative (SSI), which combines different technologies to enhance the yield of sugarcane by enabling the crop to utilize plant nutrients (Loganandhan et al., 2013). One of the methods used is the bud chip technology, also called single-bud planting (Mohanty and Nayak, 2021). It uses the axillary buds from the sugarcane stems, where a root primordial along with the small quantity of tissue attached to the bud is used to regenerate the sugarcane plant. This technology saves more than 70% of the stalk material and reduces the cost of cultivation. The advantages of using bud chips are (1) high budding rate, (2) quicker stem development, (3) harvesting time decreased, (4) increased yield, and (5) free from disease and infection (Mohanty and Nayak, 2021).

Considering the socio-economic state of China, which is dependent on the sugar industry, it is essential for scientists working on a micro level, to the farmers, working at a macro level through business enthusiasts, educationists, marketing firms, and industrialists, to come together and help toward the increase of its production (Morris et al., 2017). However, the sugarcane plant is an ideal model plant species extensively researched by plant biologists and agricultural specialists on physiology, biochemistry, and molecular biology (Li et al., 2016a). One of the most critical aspects of the plant is the development and germination of its roots and leaves. For that, the rooting system should be proficient and well established. Phytohormone, like auxin, plays a vital role in establishing the root system (Jing and Strader, 2019). There have been many studies where scientists have used indole-3-butyric acid (IBA) and indole 3-acetic acid (IAA) to study *in vitro* the role of these hormones in inducing the roots (Ludwig-Muller et al., 2005; Rout, 2006; Pacurar et al., 2014). IAA and IBA have been seen to induce adventitious root formation in mung bean (Li et al., 2016a), lotus (Libao et al., 2018), tea (Wei et al., 2014), sugarcanes (Li et al., 2020), strawberry (Hossain and Gony, 2020), carrot taproot (Khadr et al., 2020), Arabidopsis (Yang et al., 2021), and *Euryodendron excelsum* HT (Xiong et al., 2022). Studies have shown that genes expressed predominantly on IBA treatment are involved in cell replication and weakening (Brinker et al., 2004). A protein phosphatase 2A gene was upregulated during the IBA-induced adventitious root formation on Arabidopsis stem segments (Fattorini et al., 2017). Transcriptome analysis has previously been performed on sugarcane internodes (Kasirajan et al., 2018; Wang et al., 2019), root (Zeng et al., 2015; Dharshini et al., 2019; Malviya et al., 2020), leaves (Li et al., 2016a; Liu et al., 2018), mature and furled immature leaves (Manechini et al., 2021), and stalk (Zhu et al., 2021). To the best of our knowledge, this is the first-of-its-kind attempt to study the transcriptome analysis of the single bud of sugarcane in response to IBA. The present study focuses on applying IBA on single-bud seeds to find out the critical differential expressed genes and metabolic pathways on sugarcane rooting and provides a theoretical basis for enhancing sugarcane growth through phytohormone.

## Materials and Methods

### Plant Material and Pot Experiment

The sugarcane variety Guitang 55 (GT55) was obtained from the germplasm of Sugarcane Research Institute, Guangxi Academy of Agricultural Sciences, Nanning, Guangxi, China. The GT55 variety is the least susceptible to bacterial infection. Therefore, we chose GT55 as the experimental material to reduce experimental error. Single bud of sugarcane sets (length 3 cm) with 3–5th buds was taken from cane stem of variety GT55 by counting ground eyes as first buds. Single buds of GT55 sugarcane sets (length 3 cm) were cut and immersed in the IBA at different concentrations (50, 100, and 200 ppm) for 10 min. The control set was immersed into the sterilized distilled water for 10 min as control. All of the single sugarcane buds were transplanted into pots containing 3.5 kg of field soil with moderate fertility (each kg soil containing 21 g organic matter, 130 mg available N, 85 mg available P, 74 mg available K, 0.81 total N, 3.10 g total P, 7.10 g total K and pH was 6.21). Soil analysis was performed according to Solanki et al. (2019) in the Soil testing laboratory of Guangxi University, Nanning, Guangxi. The plants were grown in a greenhouse with temperatures maintained between 25 to 30°C and relative humidity between 50 to 80%. As per the requirement, one time autoclaved tap water was used to irrigate sugarcane plants. After 35 days, top visible dewlap leaf samples were detached from 5 plants for each treatment and frozen in liquid nitrogen for phytohormone analysis. Five plants samples were separated from each treatment for plant growth parameter analysis.

**Figure 1:**
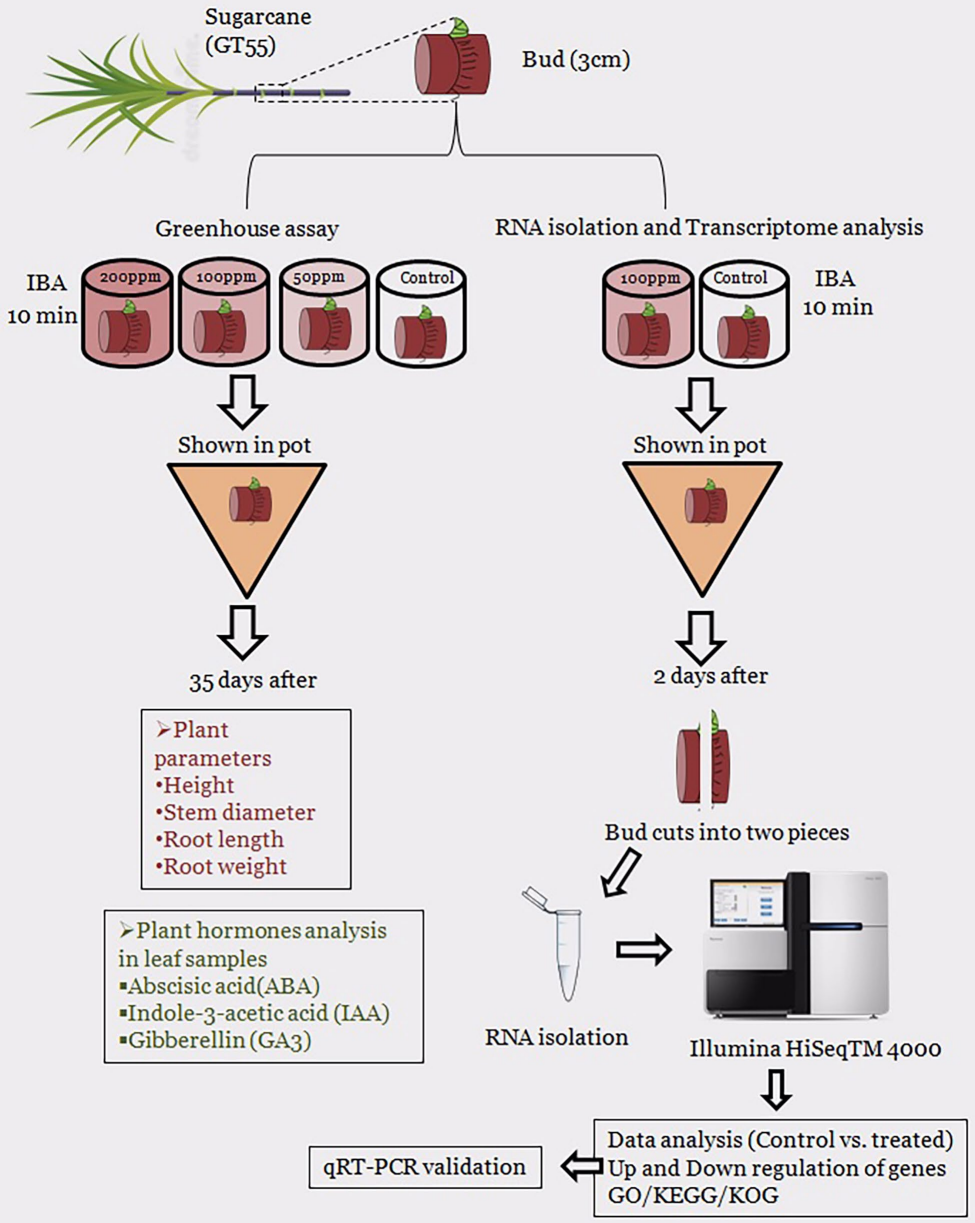
Schematic diagram of an overall research experiment that used in the current study.

Next, we performed an experiment for transcriptome analysis of IBA (100 mg/L) treated single buds of GT55 sugarcane (T) and the sterilized distilled water called control (CK). The sugarcane seedlings were transplanted into pots and watered same in the greenhouse. After 2 days, all the sugarcane buds were taken out and washed. After the sugarcane rooting of the single bud was cut two sides of the cross section and removed, the single eye sets were cut in half lengthwise. The half lengthwise were wrapped with tin paper, stored in liquid nitrogen for 40 min, and delivered by dry ice. The Schematic diagram of the overall experimental design is present in Figure 1.

### Quantification of Plant Hormones

To quantify the concentrations of the hormones, leaf extract was extracted according to Li et al. (2016a). Briefly, leaf sample (1 g) was crushed in iced mortar and pestle with 5 ml of 80% methanol (v/v) containing 1 mM butylated hydroxytoluene, and the homogenate was incubated at 4°C for 12 h, then centrifuged at 10,000 × *g* for 20 min. The supernatant was collected and then eluted through a Sep-Pak C18 cartridge (Waters, Milford, MA, United States) to get rid of the pigment. The solution was lyophilized as powder and liquefied with 2 ml of phosphate-buffered saline. Next, concentrations of abscisic acid (ABA), indole-3-acetic acid (IAA), and gibberellin (GA3) were quantified by an enzyme-linked immunosorbent assay described by Yang et al. (2001). A 96-well microtiter plate pre-coated with coating buffer containing synthetic ovalbumin conjugates for IAA, GA3, and ABA was used according to manufacturer protocol (China Agricultural University, Beijing, China). In brief, 50μLsample diluted in assay buffer was added to each well, followed by 50 μl of diluted (1:2,000) antibodies in assay buffer. The plates were incubated for 45 min at 37°C and then washed four times with scrubbing buffer, and anti-mouse IgG was added to each well and incubated for 30 min at 37°C, and washed. Next, 100 μl of a 1.5 mg mL^−1^ ortho-phenylenediamine substrate solution and 0.04% (v/v) of 30% H_2_O_2_ in substrate buffer were added. The enzyme reaction was carried out in the dark at 37°C for 15 min and then stopped by adding 50 μ L of 2 M H_2_SO_4_to each well. The absorbance was recorded at 490 nm. Concentrations of IAA, GA3, and ABA were calculated by standard curve data. All the experiments were performed in five biological replicates.

### RNA Isolation, cDNA Library Preparation, and Sequencing

Total RNA was extracted according to the manufacturer’s procedure using a TRIzol reagent kit (Invitrogen, Carlsbad, CA, United States). The quality of the RNA was determined using an Agilent 2,100 Bioanalyzer (Agilent Technologies, Palo Alto, CA, United States) and RNAse-free agarose gel electrophoresis. Following total RNA extraction, eukaryotic mRNA was enriched using Oligo (dT) beads, whereas prokaryotic mRNA was enriched using the Ribo-ZeroTM Magnetic Kit to remove rRNA (Epicentre, Madison, WI, United States). The enriched mRNA was then fragmented into short fragments with fragmentation buffer before being reverse transcribed into cDNA using random primers. DNA polymerase I, RNase H, dNTP, and buffer were used to make second-strand cDNA. The cDNA fragments were then purified using a QiaQuick PCR extraction kit (Qiagen, Venlo, Netherlands), end-repaired, poly (A) added, and ligated to Illumina sequencing adapters. The ligation products were size selected by agarose gel electrophoresis; PCR amplified and sequenced using Illumina HiSeqTM 4,000 by Gene Denovo Biotechnology Co. assembly and analysis.

The raw reads were processed under the data quality control protocol to ensure the data quality before further analysis. To filter low-quality data, fastp (Chen et al., 2018) was used to perform quality control on the offline raw reads and get clean reads. It removes low-quality reads (the number of bases with a quality value Q ≤ 20 accounts for more than 50% of the entire read). After the data is filtered, the base composition and quality distribution were analyzed to display the data quality visually. The more balanced the base composition, the higher the quality and the more accurate the subsequent analysis. The reads were assembled using the Trinity software (Grabherr et al., 2011). The software combines reads of a certain length of the overlap to form contigs, which are longer fragments without N. These give rise to unigenes, which are clusters of genes that is unique for a particular function. This software also sorts all unigenes from longest to shortest and accumulates the lengths in turn. The quality of the assembly result can be evaluated from the N50 value. When the accumulated fragment length reaches 50% of the total fragment length (the length of all Unigenes), the length and quantity of that fragment are the Unigene N50 length and quantity. The longer the Unigene N50, the smaller the quantity, the better the assembly quality. To access the integrity of the assembly, BUSCO (Simao et al., 2015) Software was used. Based on the results of each sample unigene, PCA analysis and calculation of the Pearson correlation coefficient between the control (CK) and IBA-treated (T) samples were done using the R suite to help exclude outliers.

### Functional Annotation and Classification of DEGs

The unigenes were further annotated. First, blastx was used to align the Unigene sequence according to their sequence similarity. To rule out any discrepancy, four protein databases were used. Swiss-Prot and NCBI NR database are two well-known protein databases, among which Swiss-Prot is strictly selected to eliminate any redundancy, NR (Non-Redundant Protein Sequence Database) included in NCBI, is equivalent to a cross-reference based on nucleic acid sequence, linking nucleic acid data and protein data, COG/KOG, a database for orthologous classification of gene products, and KEGG. This database systematically analyzes the metabolic pathways and functions of gene products in cells, with value of *e* < 0.00001, to obtain the protein function annotation information of the Unigene (Table 1).

**Table 1:** Summary for the BLASTx results of sugarcane transcriptome data set.

Annotation database	Annotation number
Total Unigenes	113,475
Nr	67,143
KEGG	58,470
COG	32,246
Swiss-Prot	40,453
Annotation genes	69,255
Without annotation gene	44,220

To further check the processes where the expressed unigenes play a role, the Gene Ontology (Biological Processes, Cellular Components, and Molecular Functions) analysis was done using Blast2GO (Conesa et al., 2005) and GO (Ashburner et al., 2000) software. Gene Ontology (referred to as GO) is an internationally standardized gene function classification system that provides a set of dynamically updated standard vocabulary (controlled vocabulary) to comprehensively describe the attributes of genes and gene products in organisms. On the one hand, GO function analysis provides annotations for the GO function classification of differentially expressed genes; on the other hand, it gives a significant enrichment analysis of GO functions of differentially expressed genes. Furthermore, COG and KEGG pathway annotations were performed using Blastall software against COG and KEGG databases. Principal component analysis (PCA) and heatmap analysis were performed with R package models.^1^

### Identification of Differentially Expressed Genes

The differential gene expression analysis was done using the software edgeR v14 (Robinson et al., 2010), where the reads were normalized, followed by the calculation of the probability of hypothesis test (value of p) according to the model and Finally, the multiple hypothesis test correction is carried out to obtain the FDR value (False discovery rate). Genes were considered to be significantly differentially expressed when FDR value < 0.05 and |log2FC| > 1. Differential gene expression patterns are hierarchically clustered, and heat maps are used to present clustering results. Genes with similar expression patterns may have common functions or participate in common metabolic and signaling pathways.

### Quantitative Real-Time PCR Assay

For expression validation of differentially expressed genes, special primers were designed for 8 differentially expressed genes were selected by using Premier 5.0 software. Sequencing PCR products checked the specificity of each primer set, and efficiencies of the different primer sets were similar. Details of all primers are described in Supplementary Table S1. Total RNA used as the template for reverse transcriptase reactions was initially treated with DNase I Amplification Grade enzyme (Invitrogen), an aliquot of treated RNA was used in qPCR to rule out DNA contamination. cDNA synthesis was done using SuperScript First-Strand Synthesis System for RT-PCR (Invitrogen) and random hexamers and oligo(dT) primers. qPCR reaction was performed in a volume of 20 μl containing 10 μl 2 × All-in-OneTM (GeneCopoeia, Los Angeles, United States) qPCR Mix, 2 μl cDNA, 1 μl of each 4 μM primer, and 6 μl RNase-free sterile water. To normalize the relative expression of selected genes, the GAPDH gene was used as a reference. qPCR was performed using an iQ5 Real-Time PCR Detection System (Bio-Rad, Hercules, United States). PCR reactions were performed at 95°C for 10 min, followed by 40 cycles of 95°C for 10 s, 60°C for 20 s, and 72°C for 20 s. Melting curve analysis was conducted for each reaction to confirm the specificity of the reaction, and all the cDNA samples were analyzed in triplicate. Relative expression levels of candidate genes were calculated using the 2^−ΔΔCt^ method (Livak and Schmittgen, 2001).

### Statistical Analysis

Statistical processing of plant phenotype data, hormone content, and qRT-PCR data was performed in Excel. The experiments were conducted in replicates, and data were analyzed using standard analysis of variance (ANOVA) followed by Duncan’s multiple range test (DMRT) through Origin 2017SR2 software (Northampton, MA, United States).

## Result

### Affect of IBA on Plant Growth Parameters and Plant Hormones

**Figure 2:**
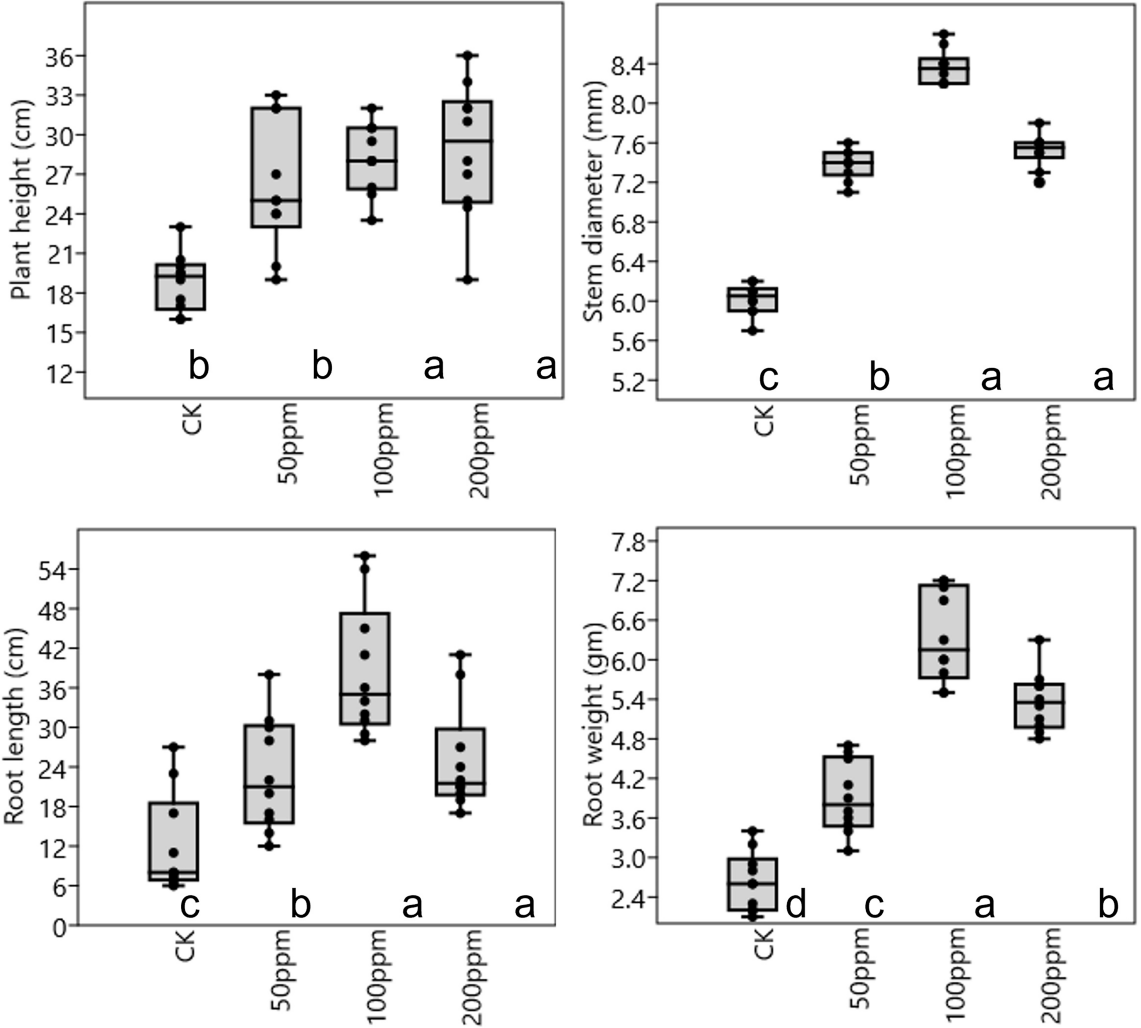
Plant physiological parameter in response to different concentrations of IBA. Bars with different letters indicate significant differences at the *p* < 0.05 according to DMRT.

**Figure 3:**
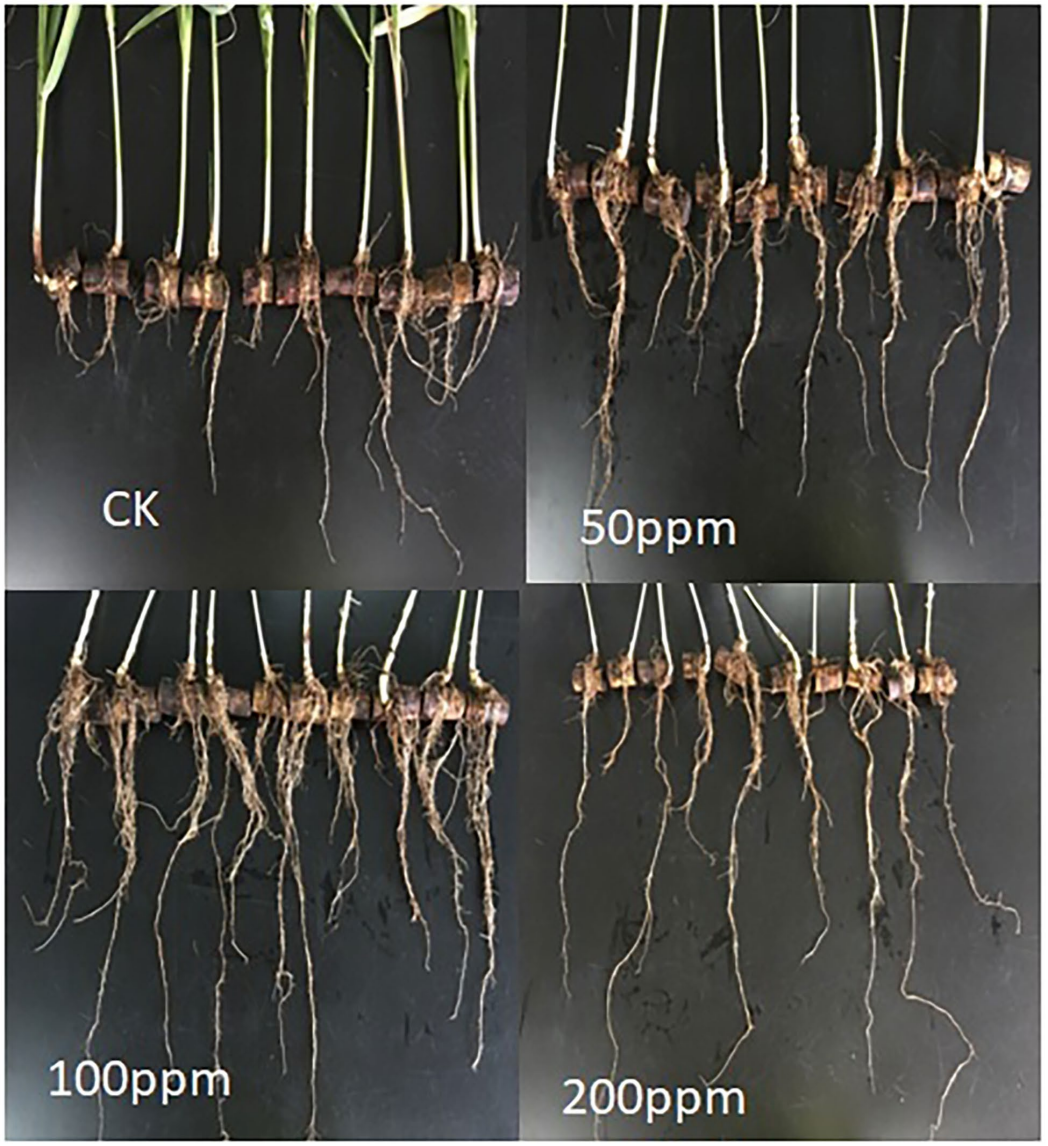
Response of different concentrations of IBA on sugarcane root.

**Figure 4:**
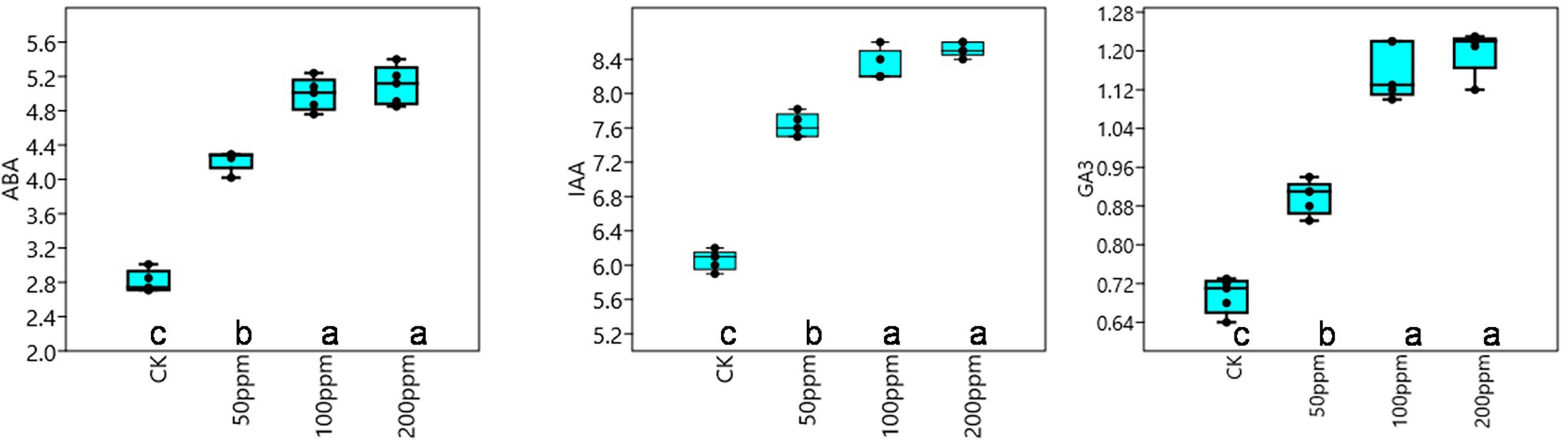
Response of IBA on different endogenous hormones, ABA (μg g^−1^ FW), IAA (nMol g^−1^ FW), and GA_3_ (nMol g^−1^ FW). Bars with different letters indicate significant differences at the *p* < 0.05 according to DMRT.

Affect of different concentrations of IBA on sugarcane bud sprouting was identified based on the greenhouse experiment. In comparison to control, IBA-treated plants were showed significant enrichment in the plant height, stem diameter, root length, and root weight (Figure 2). IBA-treated plants (100 and 200 ppm) height was enhanced significantly (*p* < 0.05) over the control. In the case of stem diameter, 100 ppm IBA showed relatively higher growth (*p* < 0.05) than the control plants. For root length, significant (*p* < 0.05) enhancement recovered with 100 ppm IBA over the control plants (Figures 2, 3). Similarly, root weight increased significantly in 100 ppm IBA-treated plants, and 200 ppm treated plants. Plant hormones analysis in response to IBA, ABA, IAA, and GA3 were determined. Compared to the control, the leaves of the 100 and 200 ppm IBA-treated plants had significant concentrations of all three hormones (Figure 4). The ABA concentration was enhanced by about 44% over to the control in IBA-treated (100 and 200 ppm) plant leaves. For IAA concentration, the maximum quantity resulted in 100 and 200 ppm treated plant leaves showing up to 27.1 and 28.8% enhancement over control, respectively. Moreover, a higher concentration of GA3 also recovered with 100, and 200 ppm treated plants than the control (Figure 4).

### Transcriptome Profile of RNA Samples of Sugarcane Buds

**Figure 5:**
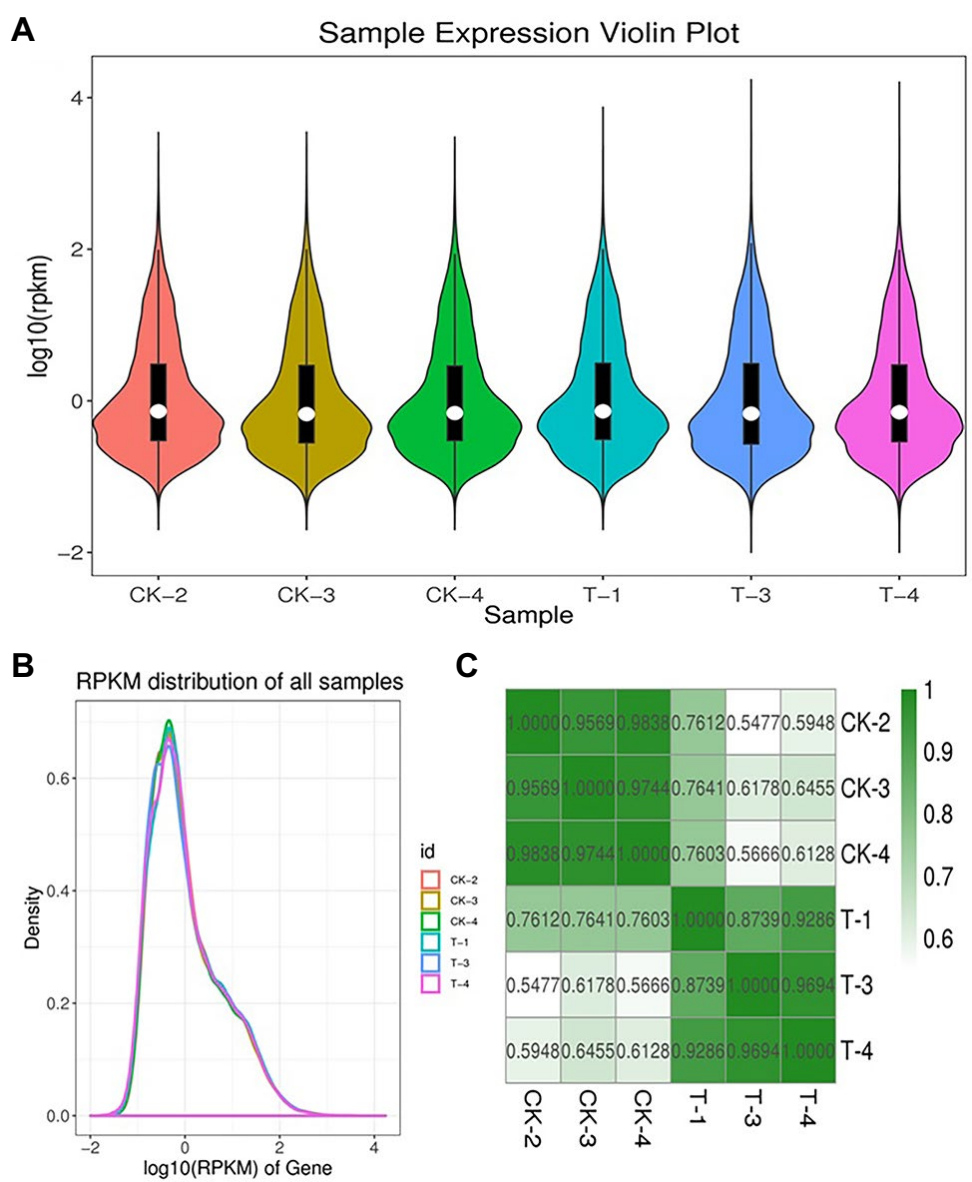
Expression levels of each sample. The violin plot showed the gene expression **(A)**. The height of the curves represents density **(B)**. Pearson correlation analysis between all biological RNA sample **(C)**.

To obtain genes involved in IBA-induced sugarcane buds, we generate two cDNA libraries from the control and IBA-treated samples (Supplementary Table S2). An average of 66.758 million clean reads with Q20 values from 99.79 to 99.86% per sample were generated. Data pre-processing statistics and quality control are presented in Supplementary Table S3. These data were then deposited in the National Center for Biotechnology Information (NCBI) with the accession number PRJNA766098. After trimming low-quality sequences, the software Trinity performed *de novo* assembly with combined cleaned reads from both libraries. As a result, the sequencing data were assembled into 113,475 unigenes with lengths ranging from 200 to 16,723 bp (mean length = 836 bp). Total length of the unigens was 94.94 Mb (94938,961 bp). GC percentage of the assembled transcripts was 50.64% and N50 length was 1,536. The genetic testing data also represents the percent sequenced gene among all samples varied from 58 to 88% (Supplementary Table S4). The unigene lengths distribution and frequency percentage are shown in Supplementary Figure S1. Distribution data showed the quality of assembly for further analysis. To investigate the difference between CK and T samples, the R statistical package was used to assess gene expression levels and determine DEGs. All genes expressed by different samples of CK and T are shown in a violin plot and curve plots (Figures 5A,B). Additionally, a principal component analysis (PCA) graph was made for both groups, and the higher difference was found between them specially, in the T samples (Supplementary Figure S2) and Pearson comparison clustering showed the similarity among the groups (Figure 5C), both groups were clustered together with replicates.

### Functional Annotation of DEGs

**Figure 6:**
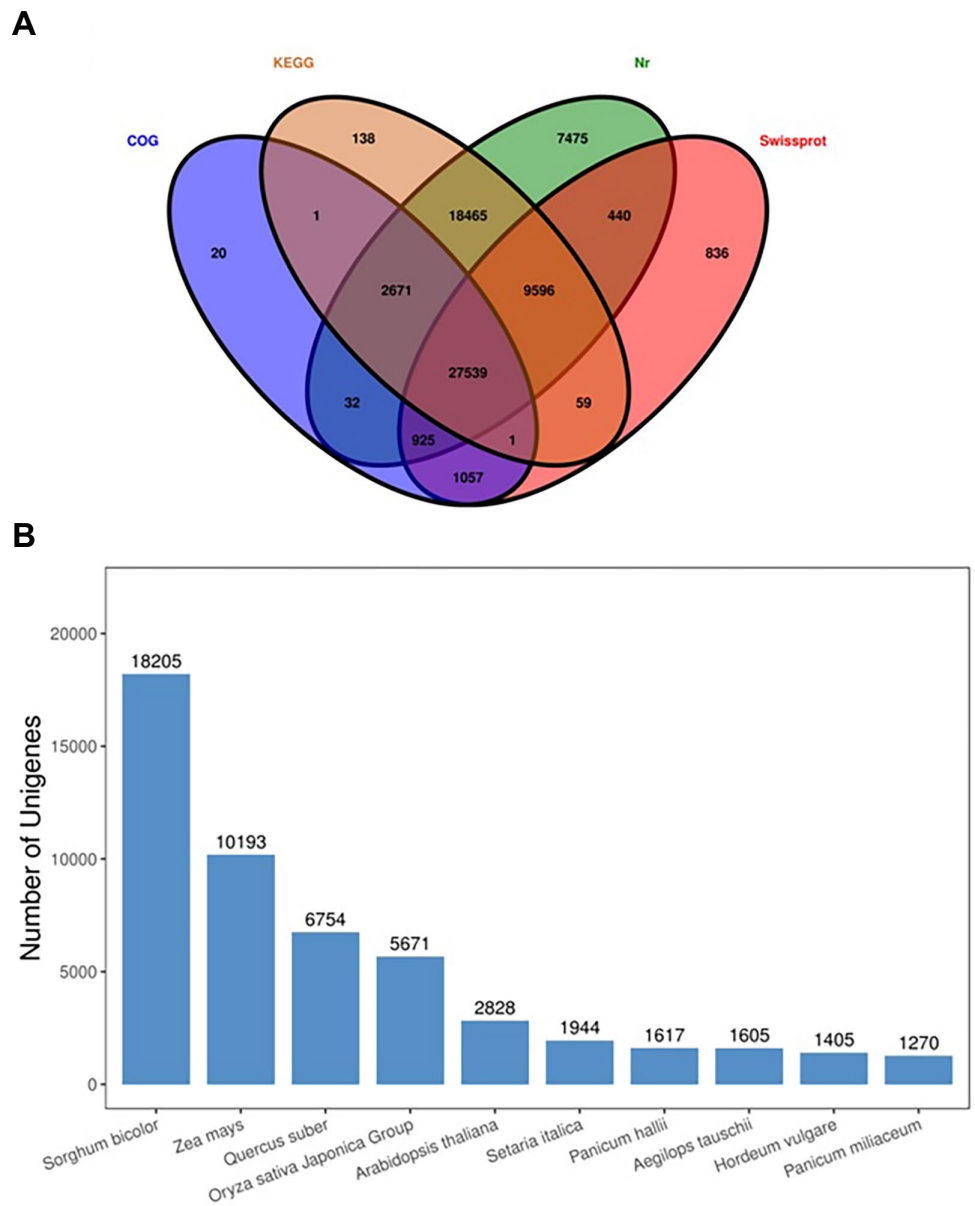
Venn diagram annotated of the four major databases **(A)**, species distribution of the top BLAST hits against the NR database **(B)**.

A total of 113,475 unigenes were annotated by blasting their sequences against four public databases. Significant annotation matches were found for 67,143 unigenes (59%) in the Non-Redundant Protein Sequence Database (Nr database), 58,470 (52%) unigenes in the Kyoto Encyclopedia of Genes and Genomes (KEGG) database, 40,453 (36%) unigenes in the Swiss-Prot database, 32,246 unigenes (28%) in the COG/KOG database. A total of 27,539 unigenes were found common among all databases. Four major databases are annotated in the Venn diagram (Figure 6A), and maximum unique genes were annotated with Nr database, followed by Swiss-Prot, KEGG, and COG. Interestingly, KEGG-Nr and KEGG-Swiss-Prot shared the maximum common unigenes. The NR database queries revealed that 67,143 unigenes annotated sequences were aligned with different plant species to known nucleotide sequences similarity with other plants. Annotation results showed 27, 15, 10, 8, 4, and 3%, matching with *Sorghum bicolor*, *Zea mays*, other species, *Quercus suber*, *Oryza sativa Japonica* Group, *Arabidopsis thaliana*, and *Searia italica*, respectively (Figure 6B). These results support the genomic similarity of sugarcane tissues with the other grass plants, such as *Zea mays* and *Oryza sativa.*

The annotation of GO terms discovered that unigenes 139,152 (50%) were assigned to biological processes, 97,637 (35%) to molecular functions, and 40,001 (14%) to cellular components (Supplementary Figure S3). Most annotated unigenes in biological processes were involved in “metabolic process” 23,453 (14%), “cellular process” 23,286 (14%), and “single-organism process” 19,215 (14%). In the cellular component category, most annotated unigenes were annotated as “cell” 22,875 (23%), “cell part” 22,820 (23%) and “organelle” 19,686 (20%). In the molecular functions, most annotated unigenes were categorized as “catalytic activity” 17,911 (45%) and “binding” 16,876 (42%). For KEGG analysis, 14,854 unigenes were classified in 141 pathways based on metabolism, genetic information processing, environmental information processing, cellular processes, and organismal systems pathway (Supplementary Figure S4). A total of 7,323 (49%) unigenes were annotated into “Metabolic pathways” of Global and overview maps, “translation” of genetic information processing, “signal transduction” of environmental information processing, “transport and catabolism” of cellular processes, and “Environmental adaptation” of organismal system. Besides, we compared unigenes with the KOG classification, and 38,945 unigenes were found to be aligned to the database and distributed into 25 categories (Supplementary Figure S5). Among them, the KOG category “general function prediction only” represented the largest group, followed by “Signal transduction mechanisms” and “posttranslational modification, protein turnover, chaperones.”

### Functional Enrichment of DGEs (CK vs. T)

**Figure 7:**
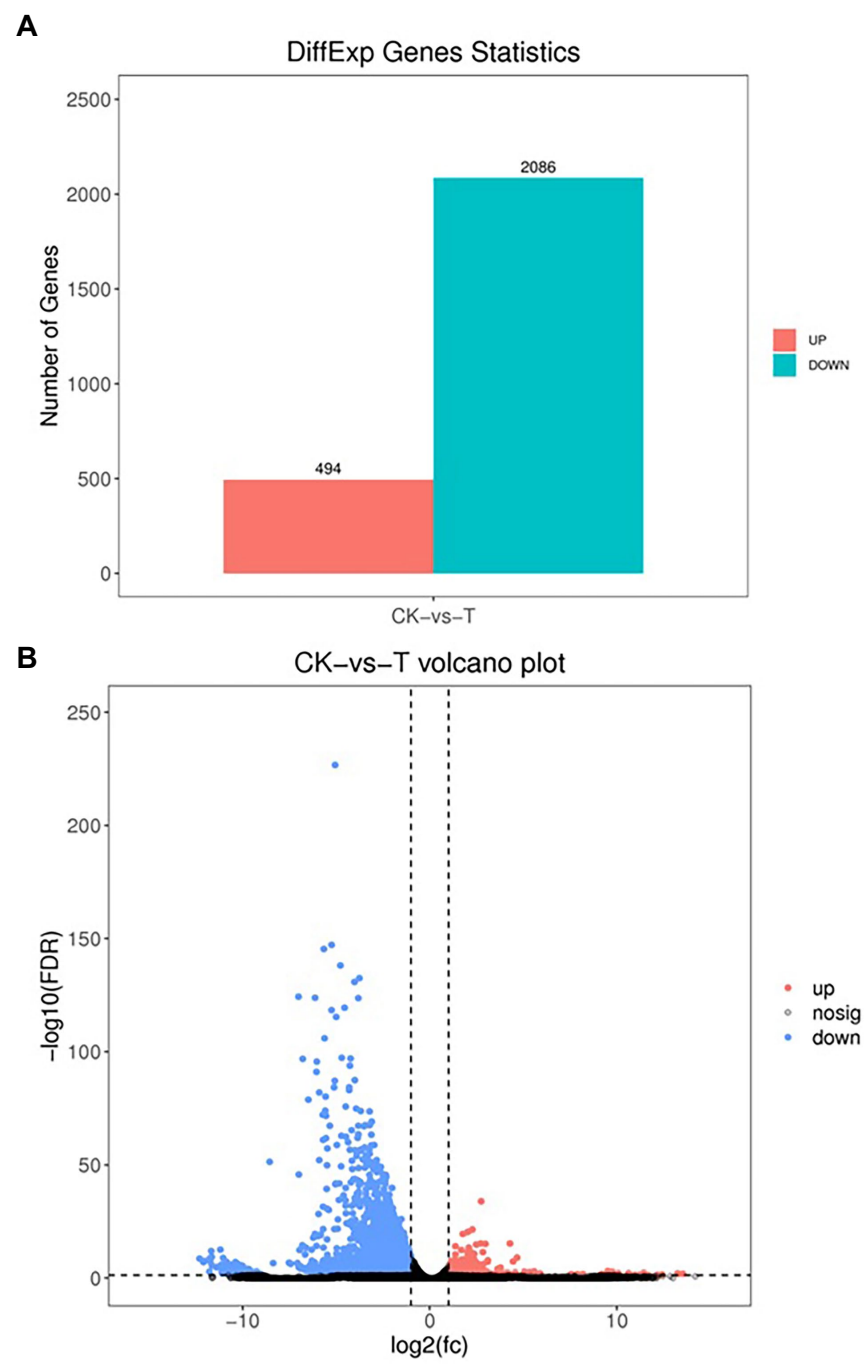
Differential expression analysis of RNA-seq **(A)**, the volcano indicates the differentially expressed genes (DEGs; **B**). Each dot in the figure signifies a particular DEG. The red dot shows upregulated DEGs. The blue dot indicates downregulated DEGs, and the dark grey dot is a non-significant differential gene.

**Figure 8:**
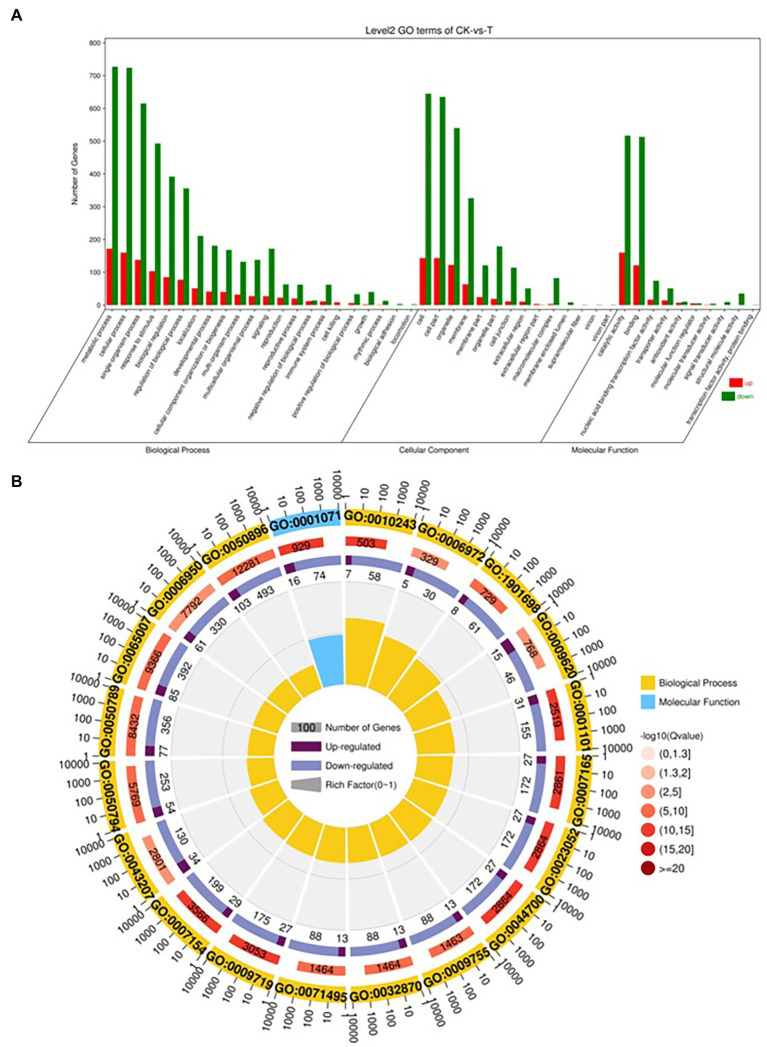
Gene classification was based on Gene Ontology (GO) analysis for differentially expressed genes (DEGs). Different classes are shown for biological processes, cellular components, and molecular functions **(A)**. GO Rich Circle Map **(B)**: (First lap: The pathway of the top 20 riches, the coordinates of the number of genes outside the circle.) Different colors represent different classes; Second lap: the number of pathways in the background gene and the Q value. The longer the gene, the smaller the Q value, the redder the color; the third circle: the upper reduction gene scale bar chart, dark purple represents the increase of the gene ratio, the light purple represents the reduction of the gene ratio; the specific values are shown below; the fourth circle: the number of differential genes in each pathway divided by all quantities), the background grid line, each grid represents 0.1).

**Figure 9:**
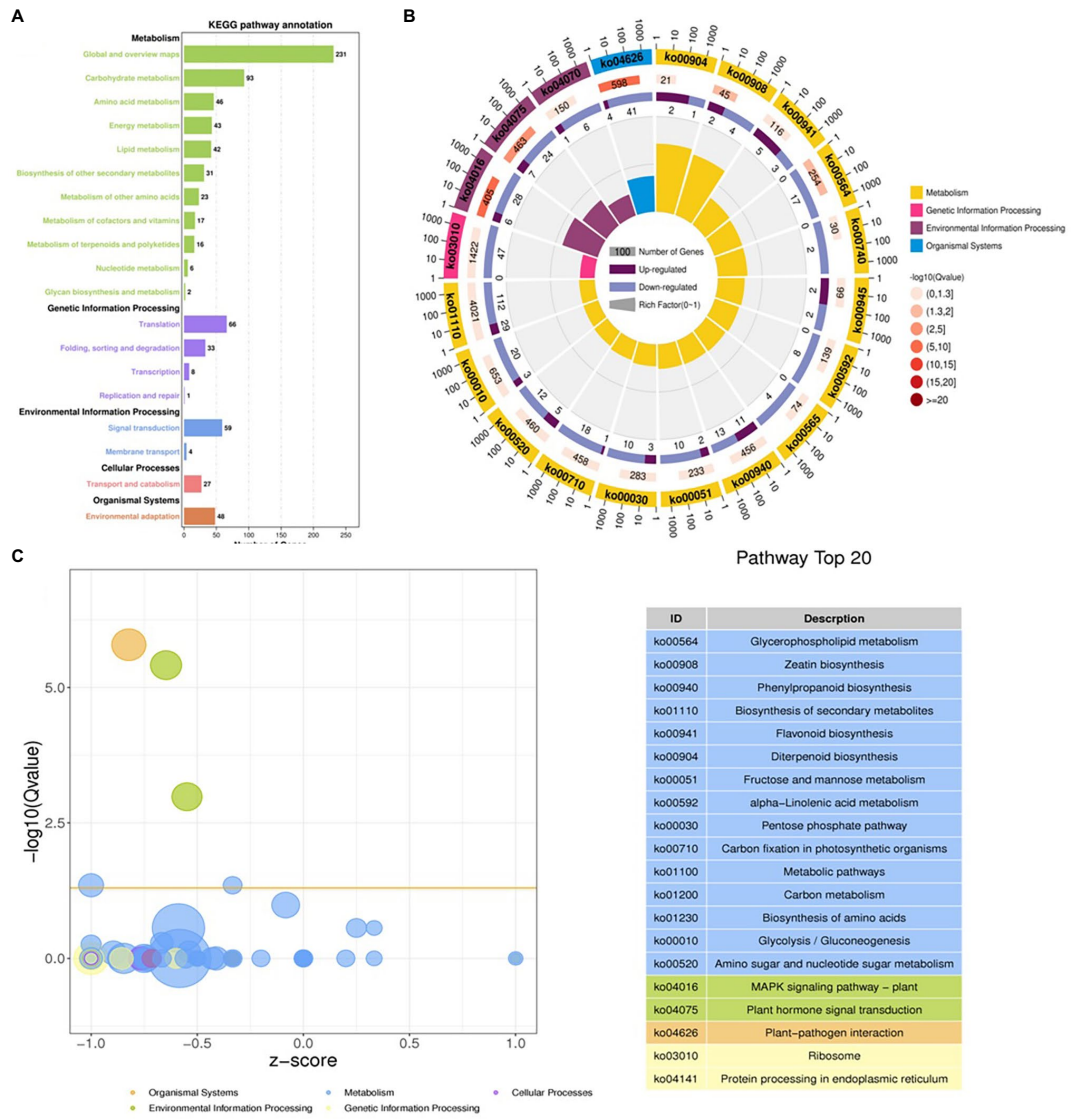
Distributions of the KEGG pathways. Putative proteins were mapped to the reference canonical pathways in the KEGG database. The bar chart shows the number of sequences in different pathway categories **(A)**, KEGG Rich Circle Map **(B)**, Bubble plot for visualizing top 20 KEGG Annotation **(C)**. The z-score predicts existence of a bias in gene regulation.

The DEGs were define by using value of *p* < 0.05 or | log2FC| ≥ 1. Among the total 2,580 DEGs, 494 upregulated and 2,086 downregulated unigenes were identified between CK and T (Figures 7A,B). Moreover, the hierarchical cluster (H-cluster) analysis of all DEGs was performed to show the expression pattern of DEGs in different samples (Supplementary Figure S6). GO, and KEGG analyses were performed to determine biological processes and functions enriched in DEGs in response to IBA (CK vs. T). Three major Go categories, biological process, cellular component, and molecular function classification, revealed that 52 pathways (24 biological processes, 17 cellular component, and 11 molecular functions) interact in CK vs. T (Figure 8A). In response to IBA, major influenced pathways in the biological process category are metabolic process, cellular process, and single-organism process. For the cellular component category, cell, cell part, organelle, membrane, and organelle part are mainly affected. In addition, catalytic activity and binding were primarily affected in the molecular function category (Figure 8A). Figure 8B showed the up and downregulation of top 20 GO IDs, and biological process genes influence more than molecular and cellular components. Supplementary Table S7 contains the list of gene functions and their *p*-values that revealed a significant enrichment of biological processes and molecular functions. Moreover, the KEGG pathway enrichment analysis of DEGs revealed that Global and overview maps, Carbohydrate metabolism, Translation Folding, sorting and degradation, Signal transduction, and Environmental adaptation were the most affected categories (Figure 9A), and the top 20 enriched pathways represented in Figure 9B, results showed the up and downregulation of genes in the pathways. In case of CK vs. T, KEGG pathways raveled significant enrichment in ko04626 Plant–pathogen interaction (*p* < 0.001), ko04016 MAPK signaling pathway—plant (*p* < 0.001, ko04075 Plant hormone signal transduction (*p* < 0.001), ko00564 Glycerophospholipid metabolism (*p* < 0.01), ko00908 Zeatin biosynthesis (*p* < 0.01, ko00940 Phenylpropanoid biosynthesis (*p* < 0.01), ko01110 Biosynthesis of secondary metabolites (*p* < 0.05), ko00904 Diterpenoid biosynthesis (*p* < 0.05) and ko00941 Flavonoid biosynthesis (*p* < 0.05; Figure 9C; Supplementary Figure S6).

### DEGs Enriched in Plant Hormone Signal Transduction Pathway

**Figure 10:**
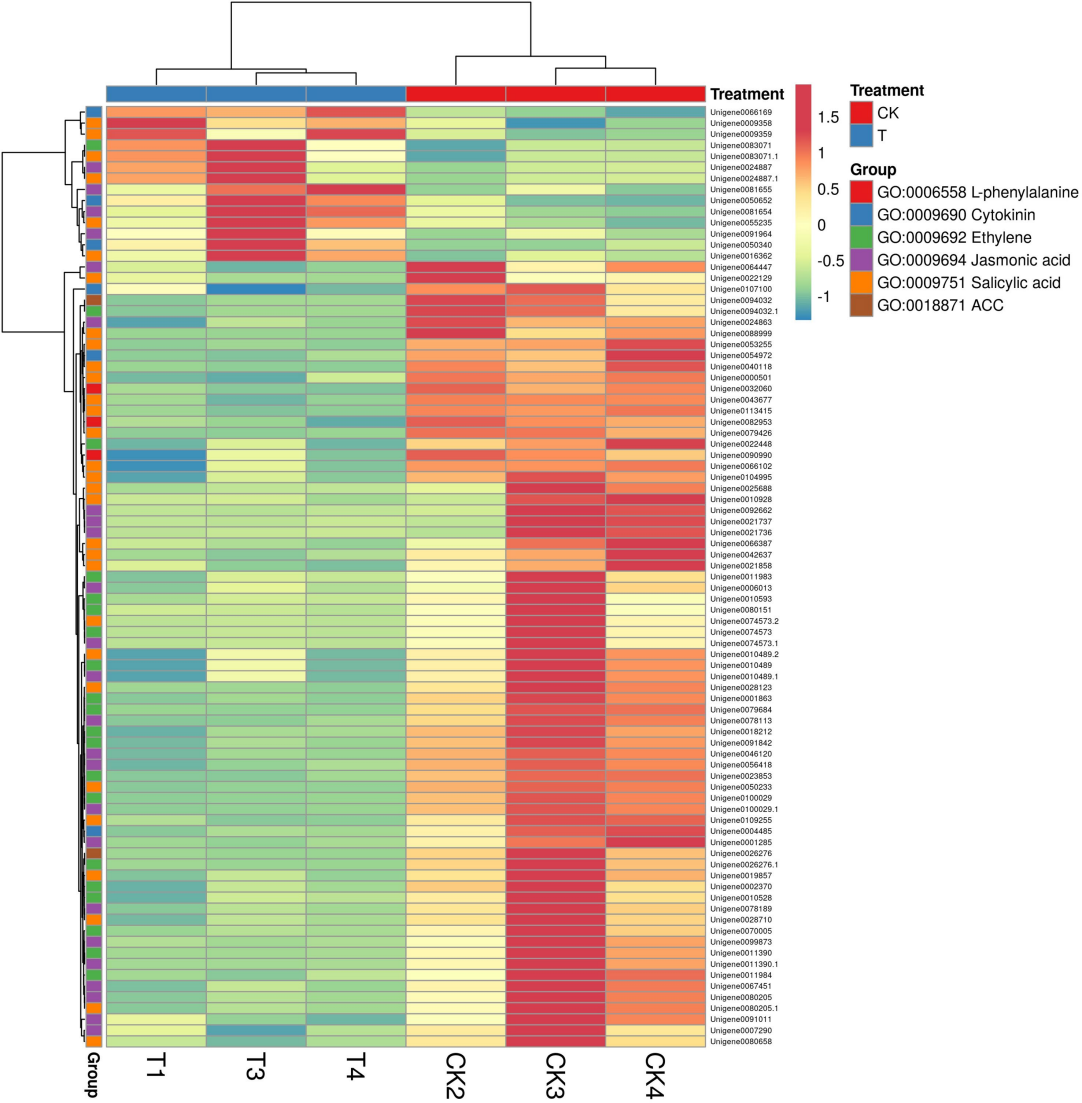
The heat map representation of genes involved in phytohormone metabolism. The expression values are RNA-seq FPKM values.

GO annotation results provide the information about the IBA treatment to enrich the essential biological process (Supplementary Table S8). GO results showed 101 genes were expressed in GO term GO:0009755 hormone-mediated signaling pathway. Next, we selected a few GO pathways to determine the response of IBA on plant hormone-related genes (Supplementary Table S9). In the cytokinin metabolic process, 6 genes were significantly (*p* = 0.004) expressed in the CK vs. T, and 3 (IPT5, CKX4, and LOGL1) were upregulated, and 3 (CKX5, LOG, and LOGL10) were downregulated. The two genes ACC1 and ACS7 of the 1-aminocyclopropane-1-carboxylate metabolic process (*p* = 0.005) were downregulated in the CK vs. T. For the response to salicylic acid genes, 30 genes were significantly (*p* = 0.005) enriched. Among them, 6 genes (AATP1, PGIP1, PGIP1, DIR1, ALD1, and NAC079) were upregulated, and 24 were downregulated. A total of 14 genes were expressed in the ethylene metabolic process (*p* = 0.06); among them, only ALD1 gene was upregulated. For the jasmonic acid metabolic process, total 24 genes were significantly (*p* = 0.006) enriched; among them, 20 genes were downregulated, while only 4 genes (At3g11180, At3g11180, SSL5, and NAC079) were upregulated. However, three genes of L-phenylalanine metabolic process were non-significantly downregulated in the biological process (Figure 10; Supplementary Table S9).

### DEGs Enriched in Other Functions of Plant

**Figure 11:**
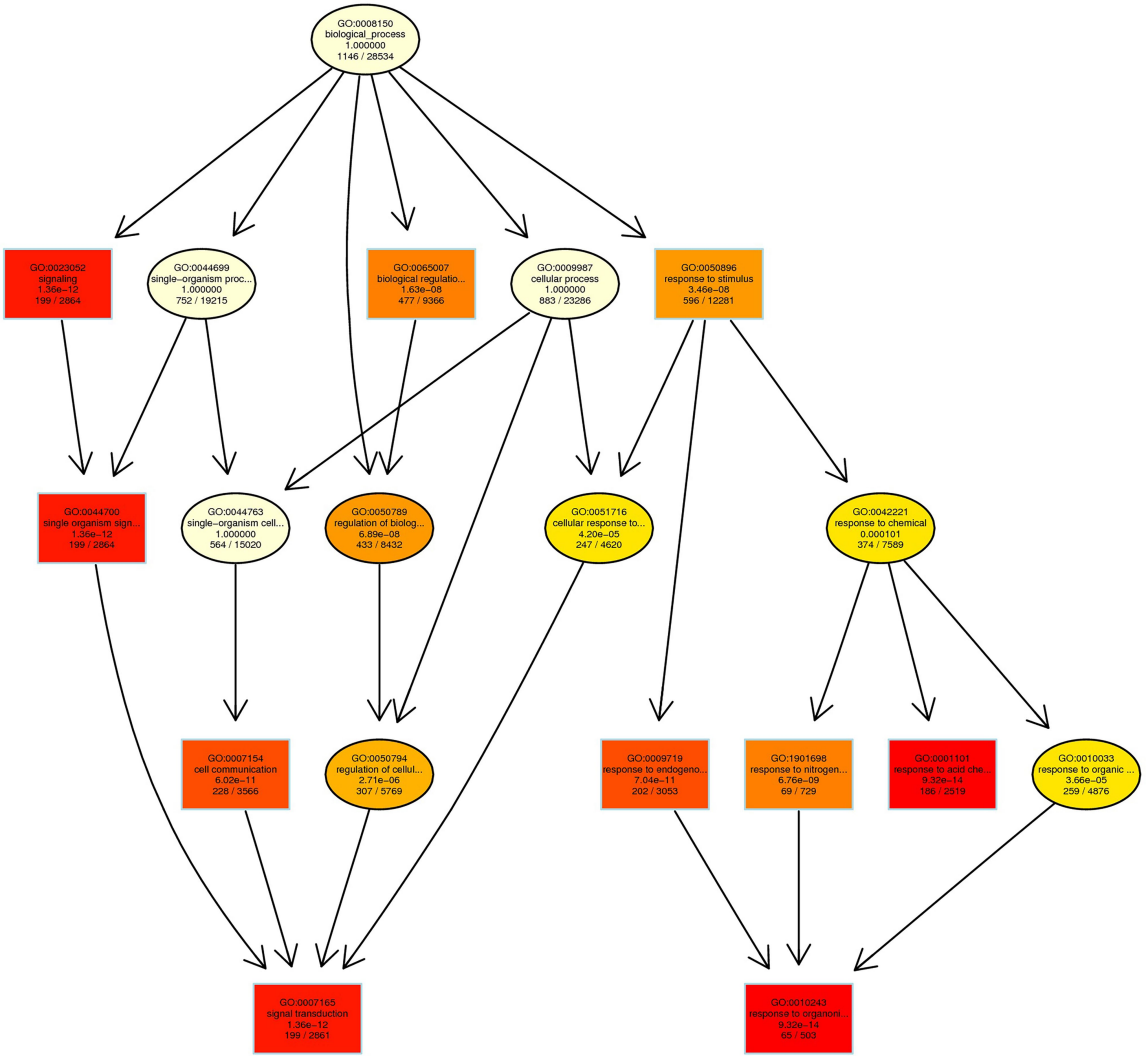
Biological process-based enriched GO term in CK vs. T. Red to yellow color exhibiting the high to low enrichment DEGs in each GO term.

In CK vs. T, the DEGs were categorized into more than 50 different terms that were dominated by biological processes pathways (Supplementary Table S9). In primary biological process related GO term includes different pathways (Figure 11). The maximum significant genes were found with GO:0050896 response to stimulus (596), and then followed by GO:0065007 biological regulation (477), GO:0050789 regulation of biological process (433), GO:0007154 cell communication (228), GO:0009719 response to endogenous stimulus (202), GO:0007165 signal transduction (199), GO:0023052 signaling (199), GO:0044700 single-organism signaling (199), GO:0001101 response to acid chemical (186), GO:1901698 response to nitrogen compound 69, and GO:0010243 response to organonitrogen compound (65; Figure 11; Supplementary Table S10). In CK vs. T, molecular function pathways classified higher with GO:0016740 transferase activity (307), GO:0016772 transferase activity, transferring phosphorus-containing groups (184), GO:0016301 kinase activity (141), GO:0016773 phosphotransferase activity, alcohol group as acceptor (120), and GO:0004672 protein kinase activity (117; Supplementary Figure S7). In cellular component related GO term includes GO:0030054 cell junction (125), GO:0005911 cell–cell junction (124), GO:0071944 cell periphery (96), GO:0030312 external encapsulating structure (85), and GO:0005618 cell wall (34; Supplementary Figure S8).

**Figure 12:**
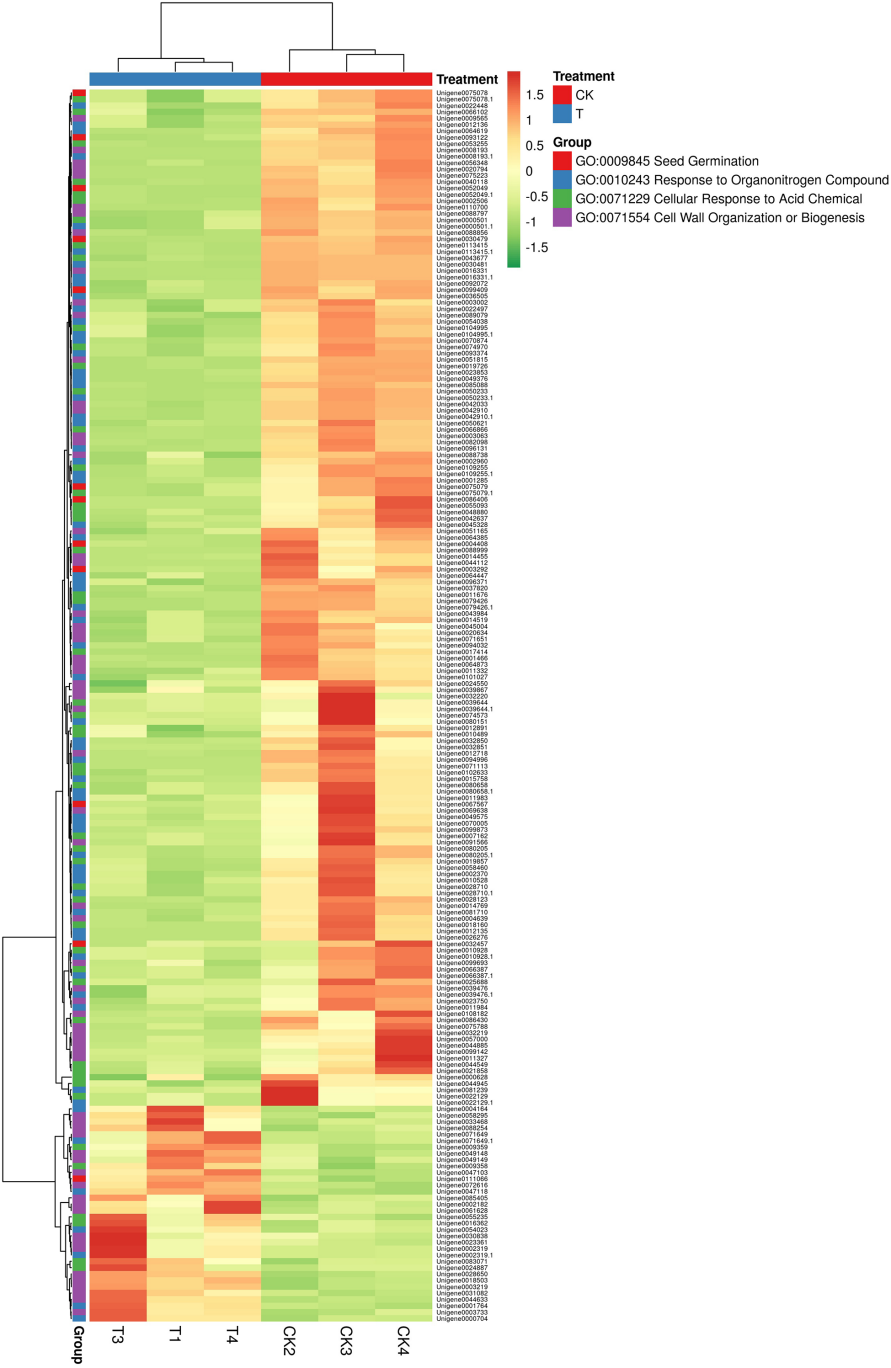
The heat map representation of genes involved in different biological functions. The expression values are RNA-seq FPKM values.

In GO term biological process, we selected a few unigenes to see their differential pattern compared to the control (Figure 12; Supplementary Table S11). For GO:0010243 response to organonitrogen compound, 65 genes were expressed significantly (*p* < 0.0001), among them, 58 were downregulated, while 7 genes (ERF1B, RLK5, CHIA (*Sorghum bicolor*), HAR1, PUB25, PTI5, and CHIA) were upregulated. In the case of GO:0071229 cellular response to acid chemical, 46 genes were downregulated and 6 genes (AATP1, PGIP1, PGIP1, DIR1, ALD1, and NAC079) were upregulated. A total of 68 genes were significantly (*p* = 0.045) enriched with the GO:0071554 cell wall organization or biogenesis. Among them, 23 genes were upregulated, and 45 genes were downregulated. For GO:0009845 seed germination, 12 differentially expressed genes were found, and among them, only one gene RR9 was upregulated (Figure 12; Supplementary Table S11).

### Validation of Gene Expression Using qRT-PCR

**Figure 13:**
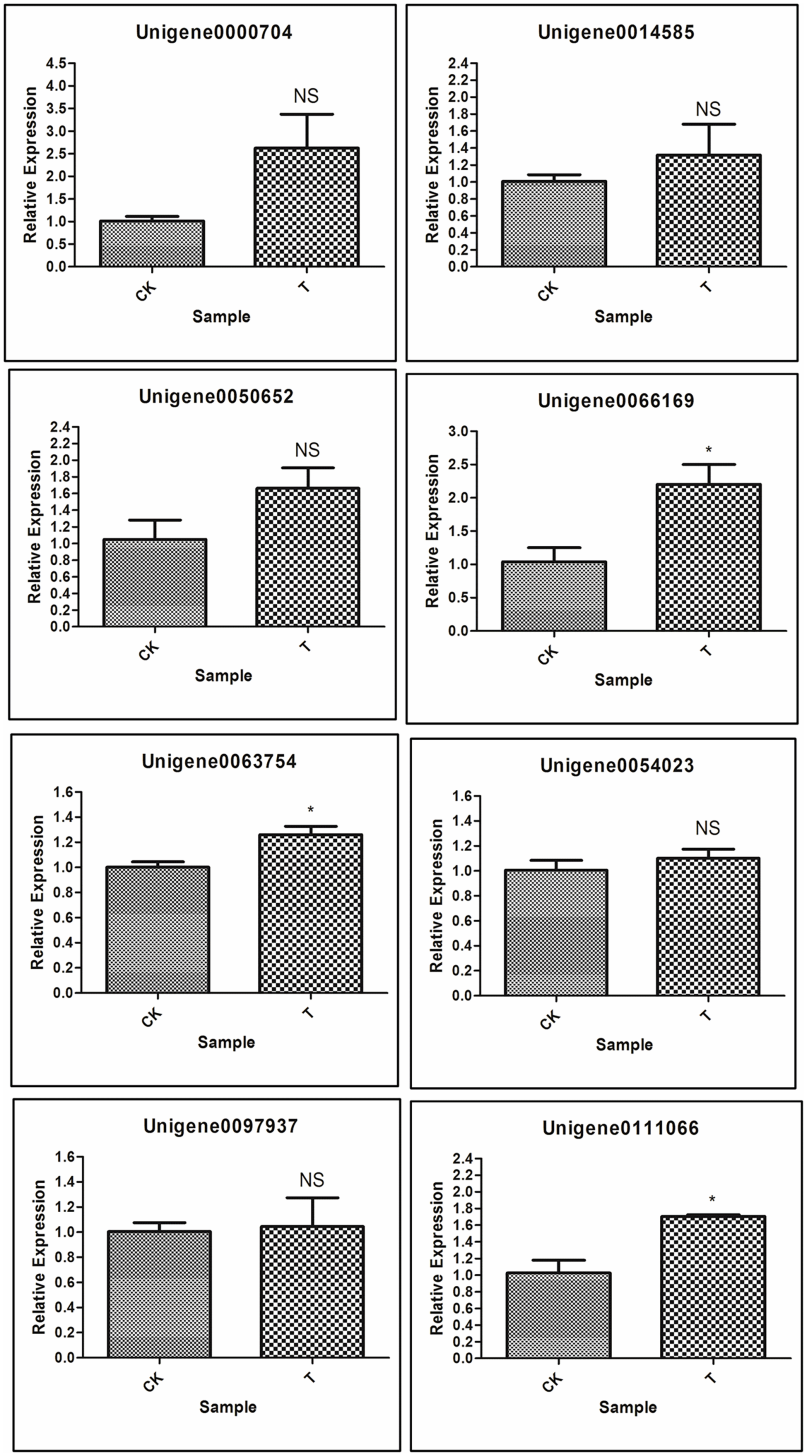
Validation of selected genes involved in rooting by qRT-PCR; Significant (*p* < 0.05) differences between CK and treatment are indicated by an asterisk.

Next, to gene function, eight differentially expressed unigenes were selected to test our RNA-seq results with real-time RT-PCR. These unigenes are related to plant hormone signal transduction, organonitrogen metabolism as well as other enzymatic processes (Table 2). Based on RNA-seq results, Unigene0054023 was showed higher log fold change as compared to other genes. The qRT-PCR results indicated that most of the genes were upregulated, but significant (*p* < 0.05) upregulation resulted only with 3 genes [Unigene0063754 Auxin-responsive protein SAUR32 (*Zea mays*), Unigene0066169 Cytokinin oxidase 2 (*Saccharum officinarum*), and Unigene0111066 two-component response regulator ORR9 (*Sorghum bicolor*)] as compared to control (Figure 13). Others genes ERF1B, MKK9, IPT5, PTI5, and GA20ox1B were showed a positive trend similar to RNA-seq analysis (Figure 13; Table 2).

**Table 2:** RNA-seq results of selected genes for qRT-PCR validation.

Gene ID	Symbol	Description	CK_mean_rpkm	T_mean_rpkm	log2(fc)	p-value	FDR
Unigene0000704	ERF1B	Ethylene-responsive transcription factor 1B (*Sorghum bicolor*)	3.99	10.91	1.45	0.0000	0.0014
Unigene0014585	MKK9	Mitogen-activated protein kinase kinase 9 (*Sorghum bicolor*)	21.96	45.63	1.06	0.0008	0.0154
Unigene0050652	IPT5	Adenylate isopentenyltransferase 5, chloroplastic (*Sorghum bicolor*)	8.31	17.82	1.10	0.0001	0.0037
Unigene0054023	PTI5	AP2-EREBP transcription factor, partial (*Zea mays*)	3.71	16.62	2.17	0.0029	0.0467
Unigene0063754	SAUR32	Auxin-responsive protein SAUR32 (*Zea mays*)	9.95	21.61	1.12	0.0000	0.0000
Unigene0066169	CKX4	Cytokinin oxidase 2 (*Saccharum officinarum*)	1.72	5.43	1.66	0.0000	0.0000
Unigene0097937	GA20ox1B	Gibberellin 20 oxidase 1 (*Saccharum* hybrid cultivar ROC22)	87.22	192.26	1.14	0.0000	0.0000
Unigene0111066	RR9	Two-component response regulator ORR9 (*Sorghum bicolor*)	10.58	23.57	1.15	0.0000	0.0001

Rpkm, reads per kilobase of transcript.

## Discussion

Bud sprouting is a critical phenomenon of the sugarcane plant that allows the development of adventitious roots (AR) during rooting. Phytohormones like IBA and Auxin are the major drivers in AR developments that regulate biological, cellular, and molecular functions (Vidoz et al., 2010; An et al., 2020; Khadr et al., 2020; Li et al., 2020). During plant rooting, IAA played a significant role at the stage of root primordia formation (Li et al., 2020; Xiong et al., 2022). Next to Auxin, IBA is a well-adopted plant growth regulator (PGR) that is frequently used for the clonal propagation of different crops. It is a need to understand the response of PGR like IBA to sugarcane bud sprouting to induce AR during *in vitro* propagation. On the other hand, inadequate cellular and molecular mechanisms information are accessible to answer how IBA regulates the AR of the bud of a long perennial grass-like sugarcane. In the present study, IBA-treated (50, 100, 200 ppm) sugarcane buds responded to a significant (*p* < 0.05) growth promotion in plant physiological parameters like root length, plant height, stem diameter with the 100 ppm concentration. Subsequently, three plant growth hormones like IAA, ABA, and GA_3_ accumulated significantly (*p* < 0.05) higher in 100 ppm concentration than control. These results are supported by several previous reports that discussed the efficacy of IBA in inducing the plant growth parameters, such as length, root weight, and leaves diameter, in different plants like strawberry and carrot plants (Hossain and Gony, 2020; Khadr et al., 2020). Auxin influence many plant growth regulating functions during developmental processes, such as cell division, elongation, and differentiation (Swarup et al., 2002; Yang et al., 2021; Xiong et al., 2022). Likewise, IBA is also known as a growth booster for the plant that regulates plant functions regards to roots, such as elongation, root hair development, lateral root formation, secondary metabolism, and nutrient transport (Muday et al., 2012; Damodaran and Stra, 2019; Wu et al., 2021). Recently, Meng et al. (2019) reported IBA treatment significantly enhanced the plant hormones at 3 days compared to control in apple rootstocks. Phytohormone-related genes, particularly those associated with auxin, ethylene, cytokinin, jasmonic acid, and salicylic acid, are vital pillars of plant growth promotion, especially in regulating adventitious AR formation (Wei et al., 2019; Li et al., 2020).

In addition to physiological data about hormones and plant growth promotion, we further analyzed the data at the molecular level by transcriptomic analysis experiment by using two kinds of samples, 100 ppm IBA-treated buds (T) and control buds (CK). In recent times, modern biotechnological, bioinformatics tools and big-data platforms have helped understand the functions of genes and gene families up to depth (Hoang et al., 2017; Abdullah et al., 2021; Faraji et al., 2021; Heidari et al., 2021; Manechini et al., 2021). In this study, RNA-seq results revealed that The IBA treatment significantly influenced DEGs regulation. An average of 66.758 million clean reads were assembled into 113,475 unigenes, and the total length of the unigenes was 94.94 Mb (94,938,961 bases). The GC percentage of the assembled transcripts was 50.64%. The N50 length was 1,536. Our results are aligned with the few previous research that generates the 59.4 mb unigenes length from basal tissue of sugarcane micro shoots treated with auxin (Li et al., 2020). In the case of CK vs. T, 86% unigenes were assembled, which is longer than reported previously in studies using the same technology (Kim et al., 2012; Yue et al., 2016; Quan et al., 2017; Li et al., 2020). Thus, the present sequencing depth contains high-quality bases for further investigation. To our knowledge, this study attempts the first time to investigate the sugarcane bud transcriptome dataset in response to IBA treatment. Additionally, the types and quantities of the genes expressed in CK and IBA-treated single-bud cuttings, along with their functions, classifications, and metabolic pathways regulating sugarcane bud sprouting. A total of 2,580 DEGs were identified, and this number was higher than similar previous studies that discussed the transcriptome of sugarcane tissues (Wei et al., 2014; Li et al., 2016a, 2020). Furthermore, *in silico* analysis showed that among 2,580 DEGs, 494 DEGs were upregulated, and 2,086 DEGs were downregulated with the cutoff FDR value < 0.05 and |log2FC| > 1. The response IBA in plants is not dominated by a single factor but by a balance of biosynthesis, metabolism, and function through various signaling pathways. Our results showed that IBA influenced the biological process dominantly, and metabolic process, cellular process, and single-organism process had a significant influence of IBA. In the case of hormone-mediated signaling pathway and IBA, upregulation of genes IPT5, CKX4, and LOGL1 and downregulated genes CKX5, LOG, and LOGL10 in the cytokinin metabolic process. Several previous studies discussed these genes in plant development in different plants (Kurakawa et al., 2007; Ramirez-Carvajal et al., 2009; Böttcher et al., 2015; Wang et al., 2020; Shibasaki et al., 2021), and Cytokinin has been known to be a negative regulator of adventitious rooting *via* its negative regulation of auxin (Ramirez-Carvajal et al., 2009; Li et al., 2016b). The two downregulated genes, ACC1 and ACS7 of the 1-aminocyclopropane-1-carboxylate metabolic process, need to understand more in the case of sugarcane. These two genes played a significant role in ethylene biosynthesis. Twenty-four genes were downregulated for the salicylic acid pathway, while 6 genes like AATP1, PGIP1, PGIP1, DIR1, ALD1, and NAC079 were upregulated. Upregulated genes may play a significant role in defense against stress-inducing factors. However, downregulation of a higher number of genes may limit the defense responses that may enhance the plant growth (Karasov et al., 2017; Smith, 2019), although it is not the absolute truth (Campos et al., 2016). A gene ALD1 was upregulated in the ethylene metabolic process among the 14 genes. These results indicated that more genes downstream of metabolic and regulatory networks might be activated under long-term stress, which would likely eventually affect sugarcane growth (Cunha et al., 2017). Similarly, another stress relating pathway (jasmonic acid) had 20 downregulated genes were found, while only four genes (At3g11180, At3g11180, SSL5, and NAC079) were upregulated. Jasmonic acid pathways played a decisive role in the defense management of plants against biotic and abiotic stresses (Antico et al., 2012; Du et al., 2013; Sanches et al., 2017; Kim et al., 2021). As per the experiment, we used a healthy sugarcane bud and healthy soil with proper moisture for the bud sprouting that limited the stress-inducing factors. The present study provides helpful information by the DEGs results of different stress regulating plant hormone pathways. It is known that sugarcane can tolerate early phase stress deficits without significantly affecting future yields (da Silva et al., 2013).

Next, to plant hormones, different biological processes were examined. Higher downregulated DEGs of GO:0010243 and ERF1B, RLK5, CHIA (*Sorghum bicolor*), HAR1, PUB25, PTI5 upregulated DEGs. These DEGs may play a significant role in the nitrogen metabolism and secondary metabolism of the plant. Zhang et al. (2020) reported the ERF1B gene played a considerable role in root development. Jinn et al. (2000) concluded that RLK5 played a significant function in floral organ abscission. CHIA and PTI5 genes are involved in plant defense to biotic and abiotic stresses (Gu et al., 2002; Su et al., 2014), HAR1 gene in nodulation (Okuma et al., 2020), and PUB25 gene in root meristem development (Wendrich et al., 2017). Next, GO:0071229 cellular response to acid chemical, AATP1, PGIP1, PGIP1, DIR1, ALD1, and NAC079 genes were upregulated, and their functions are cell development and plant defense (Song et al., 2004; Champigny et al., 2013; Yang et al., 2014; Kalunke et al., 2015; Conrath et al., 2017). A total of 23 genes were upregulated in cell wall organization or biogenesis, and one gene RR9 was upregulated with the seed germination pathway. Dissimilarity in the expression levels of specific genes encoding the cell wall components involved in cell wall modifications and the maintenance of cell wall integrity, as well as cytoskeleton-related proteins were detected in T and CK, and, perhaps, some of them were related to adventitious root induction in bud sprouting. Upregulated genes of the cell wall are associated with the cell developmental and environmental signals, including lateral root formation and root development (Villacorta-Martín et al., 2015; Pizarro and Díaz-Sala, 2022). We further validate the RNA-seq data of eight DEGs through the qRT-PCR (Figure 11; Table 2). qRT-PCR validation concluded that RNA-seq analysis data showed more accurate expression than the qRT-PCR, and based on these conclusions, we can avoid the use of qRT-PCR for gene quantification. However, systematic quantification by using two different methods could help to optimize information for the readers and new researchers.

## Conclusion

The comprehensive study shows that use of IBA on the single-bud seeds of sugarcane holds a lot of potential in sugarcane industry and showing the changes at the molecular level. The transcriptomic data generated in the present study for sugarcane provides insights into mechanisms of IBA-induced adventitious rooting and opens up economic avenues for agriculturists and farmers. Future works should characterize the functional role of these identified individual DEGs and their regulatory networks. The expression patterns of genes encoding phytohormones, cell wall development, and other function in response to IBA in sugarcane bud sprouting were concluded by the RNA-seq data. Identified DEGs and their functions may be associated with plant growth stimulation and specific modifications of the cell wall. Multiple gene profiling by cutting-edge technologies discover competitive information about bud sprouting and adventitious root formation, and these molecular maps will help to understand the expressional signature of different DEGs under different kinds of stress regulating factors, and that may help to improve the crop production against the climate resilience. Moreover, a depth study will help to stabilize the networking and functions of DEGs associated with IBA treatment and plant growth. Future activities to better understand the root growth stimulation of sugarcane under different stress like saline, drought, root inhibiting biotic pathogen, and heavy metal treatments might spotlight essential genes functions to compare with relative plant species and link with the plant evolutionary biology. Big-data information would significantly contribute for the long perennial grasses to conduct experiments and compare with the database that is not present abundantly for the grass crops like sugarcane.

## Data Availability Statement

The raw data have been deposited to the NCBI, Sequence Read Archive (SRA) database with accession number PRJNA766098. The test links are as follows: https://dataview.ncbi.nlm.nih.gov/object/PRJNA766098?reviewer=cpfmjtoasijgvpa9dq0q3ur0td.

## Author Contributions

LX, Z-ND, K-CW, W-ZW, and H-RH conceived and designed the experiments. LX, H-RH, MM, MS, and KV drafted the manuscript. TP, Y-JL, and X-YL contributed to reagents, materials, and analysis tools. MS, BK, and ED analyzed of the sequence data and performed the statistical analysis. All authors contributed to the article and approved the submitted version.

## Funding

This research was supported by the National key research and development program of China (2020YFD1000600), Government Booting Local Program (ZY20158007), Guangxi Natural Science Foundation (2020GXNSFBA159024), National Natural Science Foundation of China (32060468), and Guangxi Academy of Agricultural Sciences, Nanning, Guangxi, China, for providing the necessary facilities for this study.

## Conflict of Interest

The authors declare that the research was conducted in the absence of any commercial or financial relationships that could be construed as a potential conflict of interest.

## Publisher’s Note

All claims expressed in this article are solely those of the authors and do not necessarily represent those of their affiliated organizations, or those of the publisher, the editors and the reviewers. Any product that may be evaluated in this article, or claim that may be made by its manufacturer, is not guaranteed or endorsed by the publisher.

## Footnotes

^1^ http://www.r-project.org/
