# Supplementary material for: Transcriptome Analysis Reveals a Gene Expression Pattern That Contributes to Sugarcane Bud Propagation Induced by Indole-3-Butyric Acid
Source: Front Plant Sci. 2022 Mar 17;13:852886. doi: 10.3389/fpls.2022.852886 (PMC8969426; doi:10.3389/fpls.2022.852886)
Supplement: Supplementary file 1 [file Data_Sheet_1.docx]

Title: Transcriptome Analysis Reveals a Gene Expression Pattern that Contributes to Sugarcane Bud Propagation Induced by indole-3-butyric acid

Lin Xu^1 †^, Zhi-Nian Deng^1 †^, Kai-Chao Wu^1, †^, Mukesh Kumar Malviya^1^, Manoj Kumar Solanki^2^, Krishan K. Verma^1^, Tian Pang^1^, Yi-Jie Li^1^, Xiao-Yan Liu^1^, Brijendra Kumar Kashyap^3^, Eldessoky S. Dessoky^4^, Wei-Zan Wang^1,^*, Hai-Rong Huang^1^*

^1^Key Laboratory of Sugarcane Biotechnology and Genetic Improvement (Guangxi), Ministry of Agriculture and Rural Area, Sugarcane Research Center, Chinese Academy of Agricultural Sciences, Guangxi Key Laboratory of Sugarcane Genetic Improvement, Sugarcane Research Institute, Guangxi Academy of Agricultural Sciences, Nanning - 530007, Guangxi, China

^2^Plant Cytogenetics and Molecular Biology Group, Institute of Biology, Biotechnology and Environmental Protection, Faculty of Natural Sciences, University of Silesia in Katowice, Katowice, Poland

^3^Department of Biotechnology Engineering, Institute of Engineering and Technology, Bundelkhand University, Jhansi-284128, Uttar Pradesh, India

^4^Department of Plant Genetic Transformation, Agriculture Genetic Engineering Research Institute, Agriculture Research Center, P.O. Box 12619, Giza, Egypt

^†^These authors have contributed equally to this work

***Corresponding authors**

E-mails: [hhrong15937@126.com](mailto:hhrong15937@126.com) (H.-R. Huang), Cell no. +8613086710913; [wwz003411@126.com](mailto:wwz003411@126.com) (W.-Z. Wang), Cell no. +86 15877130580

**Supplementary Materials:**

**Table S1: Primers used in qRT PCR analysis**

| Primer |  | 5'--3' | Tm（℃） | Product (bp） |
| --- | --- | --- | --- | --- |
| Unigene0066169 | F | CTATCTCCATCTCACGGTCGG | 59.4 | 88 |
|  | R | CATCTCCTCTTCCTGCGGTC | 59.4 |  |
| Unigene0063754 | F | CACAGCAGCAACCACTTCCA | 60.1 | 542 |
|  | R | AGAGAAGCGAGGAGGACACA | 57 |  |
| Unigene0111066 | F | TAAGGCAAACAGCCAAGGGA | 60.7 | 118 |
|  | R | GAGAAAGGGAGCAGACAACGA | 59.4 |  |
| Unigene0000704 | F | TGCTGCTTCTTGGAACCCTT | 59.8 | 150 |
|  | R | CGACTCCTTCTCCGACGACA | 60.7 |  |
| Unigene0054023 | F | TTTCAATCAGGGAAGAGGCATAG | 60.5 | 439 |
|  | R | CAGCGACCATCATCACCAACA | 62.2 |  |
| Unigene0014585 | F | CGAACTTGTTGCCTCTTGGGA | 62.5 | 163 |
|  | R | CTGCCCCCTGCGATTAACT | 60 |  |
| Unigene0050652 | F | GTGACCAGGCAGAGTAGCG | 56.1 | 189 |
|  | R | CCCAAATCAACAGTGCCG | 57.5 |  |
| Unigene0097937 | F | TCTATCTATCACTTGGCACTCTG | 54.3 | 86 |
|  | R | CTCTGCTTGCTTTGCTACCC | 57.9 |  |
| Unigene0019704 | F | TTCCTCATCGTCACCTACTCTC | 56 | 211 |
|  | R | ACAACCAATCAAGCCAAGCAAG | 61.9 |  |

**Table S2: Replicates of each group, control (CK) and IBA treated (T), respectively.**

| Item | Name | Biological information | Treatment |
| --- | --- | --- | --- |
| GDR20050015 | CK1 | CK-1 | CK |
| GDR20050015 | CK2 | CK-2 |  |
| GDR20050015 | CK3 | CK-3 |  |
| GDR20050015 | CK4 | CK-4 |  |
| GDR20050015 | CK5 | CK-5 |  |
| GDR20050015 | T1 | T-1 | T |
| GDR20050015 | T2 | T-2 |  |
| GDR20050015 | T3 | T-3 |  |
| GDR20050015 | T4 | T-4 |  |
| GDR20050015 | T5 | T-5 |  |

**Table S3: Data pre-processing statistics and quality control**

| Sample | RawData | CleanData(%) | Adaper(%) | LowQuality(%) |
| --- | --- | --- | --- | --- |
| CK-1 | 75012924 | 74874918 (99.82%) | 23224 (0.03%) | 114782 (0.15%) |
| CK-2 | 60413560 | 60291210 (99.80%) | 21134 (0.03%) | 101216 (0.17%) |
| CK-3 | 67596006 | 67480332 (99.83%) | 19236 (0.03%) | 96438 (0.14%) |
| CK-4 | 64855608 | 64726232 (99.80%) | 20986 (0.03%) | 108390 (0.17%) |
| CK-5 | 71347726 | 71221834 (99.82%) | 21456 (0.03%) | 104436 (0.15%) |
| T-1 | 71115996 | 70975850 (99.80%) | 24078 (0.03%) | 116068 (0.16%) |
| T-2 | 68855688 | 68758528 (99.86%) | 16128 (0.02%) | 81032 (0.12%) |
| T-3 | 63383390 | 63253382 (99.79%) | 22118 (0.03%) | 107890 (0.17%) |
| T-4 | 70364772 | 70225724 (99.80%) | 24166 (0.03%) | 114882 (0.16%) |
| T-5 | 55878836 | 55775702 (99.82%) | 17906 (0.03%) | 85228 (0.15%) |

**Table S4: Genetic testing statistics**

| Sample | Total genes | Sequenced total gene (%) |
| --- | --- | --- |
| all | 113475 | 99569 (87.75%) |
| CK-2 | 113475 | 66890 (58.95%) |
| CK-3 | 113475 | 68228 (60.13%) |
| CK-4 | 113475 | 67608 (59.58%) |
| T-1 | 113475 | 67415 (59.41%) |
| T-3 | 113475 | 65284 (57.53%) |
| T-4 | 113475 | 68971 (60.78%) |

**Table S5: Details of GO term and DEGs.**

| GO ID | GO Term description | number_of_Unigene (All) |
| --- | --- | --- |
| **Biological Process** |  |  |
| GO:0008152 | metabolic process | 23453 |
| GO:0009987 | cellular process | 23286 |
| GO:0044699 | single-organism process | 19215 |
| GO:0050896 | response to stimulus | 12281 |
| GO:0065007 | biological regulation | 9366 |
| GO:0050789 | regulation of biological process | 8432 |
| GO:0071840 | cellular component organization or biogenesis | 7695 |
| GO:0051179 | Localization | 7355 |
| GO:0032502 | developmental process | 6581 |
| GO:0032501 | multicellular organismal process | 4633 |
| GO:0051704 | multi-organism process | 3048 |
| GO:0000003 | reproduction | 3034 |
| GO:0022414 | reproductive process | 2983 |
| GO:0023052 | signaling | 2864 |
| GO:0040007 | growth | 1305 |
| GO:0002376 | immune system process | 1194 |
| GO:0048519 | negative regulation of biological process | 975 |
| GO:0048518 | positive regulation of biological process | 939 |
| GO:0048511 | rhythmic process | 267 |
| GO:0022610 | biological adhesion | 128 |
| GO:0040011 | locomotion | 50 |
| GO:0098754 | detoxification | 35 |
| GO:0001906 | cell killing | 32 |
| GO:0007610 | behavior | 1 |
| **Cellular Component** |  |  |
| GO:0005623 | cell | 22875 |
| GO:0044464 | cell part | 22820 |
| GO:0043226 | organelle | 19686 |
| GO:0016020 | membrane | 10209 |
| GO:0044422 | organelle part | 8139 |
| GO:0044425 | membrane part | 4617 |
| GO:0032991 | macromolecular complex | 4329 |
| GO:0030054 | cell junction | 2742 |
| GO:0005576 | extracellular region | 1403 |
| GO:0031974 | membrane-enclosed lumen | 372 |
| GO:0019012 | virion | 146 |
| GO:0044423 | virion part | 146 |
| GO:0044421 | extracellular region part | 54 |
| GO:0099512 | supramolecular fiber | 46 |
| GO:0031012 | extracellular matrix | 21 |
| GO:0009295 | nucleoid | 20 |
| GO:0044420 | extracellular matrix component | 12 |
| **Molecular Function** |  |  |
| GO:0003824 | catalytic activity | 17911 |
| GO:0005488 | binding | 16876 |
| GO:0005215 | transporter activity | 2040 |
| GO:0005198 | structural molecule activity | 1140 |
| GO:0001071 | nucleic acid binding transcription factor activity | 929 |
| GO:0098772 | molecular function regulator | 274 |
| GO:0016209 | antioxidant activity | 273 |
| GO:0004871 | signal transducer activity | 225 |
| GO:0060089 | molecular transducer activity | 214 |
| GO:0009055 | electron carrier activity | 54 |
| GO:0000988 | transcription factor activity, protein binding | 53 |
| GO:0045182 | translation regulator activity | 12 |

**Table S6: Details of KEGG class, pathways and DEGs**.

| KEGG_B_class | Pathway | Count (14854) | Pathway_IDs |
| --- | --- | --- | --- |
| Global and overview maps | Metabolic pathways | 7323 | ko01100 |
| Global and overview maps | Biosynthesis of secondary metabolites | 4021 | ko01110 |
| Global and overview maps | Carbon metabolism | 1433 | ko01200 |
| Translation | Ribosome | 1422 | ko03010 |
| Global and overview maps | Biosynthesis of amino acids | 987 | ko01230 |
| Folding, sorting and degradation | Protein processing in endoplasmic reticulum | 879 | ko04141 |
| Carbohydrate metabolism | Glycolysis / Gluconeogenesis | 653 | ko00010 |
| Energy metabolism | Oxidative phosphorylation | 641 | ko00190 |
| Transcription | Spliceosome | 630 | ko03040 |
| Environmental adaptation | Plant-pathogen interaction | 598 | ko04626 |
| Transport and catabolism | Endocytosis | 566 | ko04144 |
| Translation | RNA transport | 558 | ko03013 |
| Carbohydrate metabolism | Pyruvate metabolism | 529 | ko00620 |
| Carbohydrate metabolism | Starch and sucrose metabolism | 510 | ko00500 |
| Carbohydrate metabolism | Glyoxylate and dicarboxylate metabolism | 474 | ko00630 |
| Carbohydrate metabolism | Citrate cycle (TCA cycle) | 472 | ko00020 |
| Signal transduction | Plant hormone signal transduction | 463 | ko04075 |
| Carbohydrate metabolism | Amino sugar and nucleotide sugar metabolism | 460 | ko00520 |
| Energy metabolism | Carbon fixation in photosynthetic organisms | 458 | ko00710 |
| Biosynthesis of other secondary metabolites | Phenylpropanoid biosynthesis | 456 | ko00940 |
| Amino acid metabolism | Cysteine and methionine metabolism | 432 | ko00270 |
| Metabolism of other amino acids | Glutathione metabolism | 408 | ko00480 |
| Signal transduction | MAPK signaling pathway - plant | 405 | ko04016 |
| Folding, sorting and degradation | Ubiquitin mediated proteolysis | 359 | ko04120 |
| Nucleotide metabolism | Purine metabolism | 337 | ko00230 |
| Transport and catabolism | Peroxisome | 329 | ko04146 |
| Transport and catabolism | Phagosome | 324 | ko04145 |
| Global and overview maps | Fatty acid metabolism | 302 | ko01212 |
| Folding, sorting and degradation | RNA degradation | 295 | ko03018 |
| Global and overview maps | 2-Oxocarboxylic acid metabolism | 284 | ko01210 |
| Carbohydrate metabolism | Pentose phosphate pathway | 283 | ko00030 |
| Amino acid metabolism | Alanine, aspartate and glutamate metabolism | 282 | ko00250 |
| Translation | mRNA surveillance pathway | 275 | ko03015 |
| Lipid metabolism | Glycerolipid metabolism | 262 | ko00561 |
| Lipid metabolism | Glycerophospholipid metabolism | 254 | ko00564 |
| Amino acid metabolism | Glycine, serine and threonine metabolism | 250 | ko00260 |
| Lipid metabolism | Fatty acid degradation | 249 | ko00071 |
| Amino acid metabolism | Tryptophan metabolism | 240 | ko00380 |
| Amino acid metabolism | Arginine and proline metabolism | 239 | ko00330 |
| Carbohydrate metabolism | Fructose and mannose metabolism | 233 | ko00051 |
| Folding, sorting and degradation | Proteasome | 225 | ko03050 |
| Carbohydrate metabolism | Galactose metabolism | 212 | ko00052 |
| Translation | Ribosome biogenesis in eukaryotes | 211 | ko03008 |
| Amino acid metabolism | Valine, leucine and isoleucine degradation | 209 | ko00280 |
| Metabolism of other amino acids | beta-Alanine metabolism | 193 | ko00410 |
| Carbohydrate metabolism | Propanoate metabolism | 186 | ko00640 |
| Translation | Aminoacyl-tRNA biosynthesis | 181 | ko00970 |
| Amino acid metabolism | Tyrosine metabolism | 178 | ko00350 |
| Nucleotide metabolism | Pyrimidine metabolism | 172 | ko00240 |
| Carbohydrate metabolism | Pentose and glucuronate interconversions | 171 | ko00040 |
| Metabolism of other amino acids | Cyanoamino acid metabolism | 170 | ko00460 |
| Carbohydrate metabolism | Ascorbate and aldarate metabolism | 167 | ko00053 |
| Metabolism of cofactors and vitamins | Porphyrin and chlorophyll metabolism | 164 | ko00860 |
| Amino acid metabolism | Arginine biosynthesis | 163 | ko00220 |
| Energy metabolism | Nitrogen metabolism | 161 | ko00910 |
| Energy metabolism | Photosynthesis | 156 | ko00195 |
| Folding, sorting and degradation | Protein export | 154 | ko03060 |
| Lipid metabolism | Fatty acid biosynthesis | 153 | ko00061 |
| Metabolism of terpenoids and polyketides | Terpenoid backbone biosynthesis | 153 | ko00900 |
| Signal transduction | Phosphatidylinositol signaling system | 150 | ko04070 |
| Amino acid metabolism | Phenylalanine metabolism | 149 | ko00360 |
| Amino acid metabolism | Lysine degradation | 139 | ko00310 |
| Lipid metabolism | alpha-Linolenic acid metabolism | 139 | ko00592 |
| Carbohydrate metabolism | Inositol phosphate metabolism | 133 | ko00562 |
| Lipid metabolism | Biosynthesis of unsaturated fatty acids | 128 | ko01040 |
| Replication and repair | Nucleotide excision repair | 127 | ko03420 |
| Replication and repair | DNA replication | 125 | ko03030 |
| Metabolism of cofactors and vitamins | Ubiquinone and other terpenoid-quinone biosynthesis | 120 | ko00130 |
| Carbohydrate metabolism | Butanoate metabolism | 120 | ko00650 |
| Environmental adaptation | Circadian rhythm - plant | 120 | ko04712 |
| Energy metabolism | Sulfur metabolism | 118 | ko00920 |
| Biosynthesis of other secondary metabolites | Flavonoid biosynthesis | 116 | ko00941 |
| Replication and repair | Homologous recombination | 116 | ko03440 |
| Amino acid metabolism | Phenylalanine, tyrosine and tryptophan biosynthesis | 114 | ko00400 |
| Glycan biosynthesis and metabolism | N-Glycan biosynthesis | 114 | ko00510 |
| Lipid metabolism | Steroid biosynthesis | 111 | ko00100 |
| Transcription | RNA polymerase | 111 | ko03020 |
| Glycan biosynthesis and metabolism | Various types of N-glycan biosynthesis | 110 | ko00513 |
| Membrane transport | ABC transporters | 109 | ko02010 |
| Transport and catabolism | Autophagy - other eukaryotes | 100 | ko04136 |
| Metabolism of other amino acids | Selenocompound metabolism | 99 | ko00450 |
| Biosynthesis of other secondary metabolites | Tropane, piperidine and pyridine alkaloid biosynthesis | 97 | ko00960 |
| Lipid metabolism | Sphingolipid metabolism | 94 | ko00600 |
| Transcription | Basal transcription factors | 87 | ko03022 |
| Folding, sorting and degradation | SNARE interactions in vesicular transport | 85 | ko04130 |
| Lipid metabolism | Fatty acid elongation | 84 | ko00062 |
| Biosynthesis of other secondary metabolites | Isoquinoline alkaloid biosynthesis | 81 | ko00950 |
| Replication and repair | Mismatch repair | 79 | ko03430 |
| Amino acid metabolism | Histidine metabolism | 77 | ko00340 |
| Lipid metabolism | Ether lipid metabolism | 74 | ko00565 |
| Metabolism of cofactors and vitamins | Thiamine metabolism | 74 | ko00730 |
| Metabolism of terpenoids and polyketides | Carotenoid biosynthesis | 73 | ko00906 |
| Replication and repair | Base excision repair | 70 | ko03410 |
| Metabolism of cofactors and vitamins | Pantothenate and CoA biosynthesis | 68 | ko00770 |
| Energy metabolism | Photosynthesis - antenna proteins | 66 | ko00196 |
| Biosynthesis of other secondary metabolites | Stilbenoid, diarylheptanoid and gingerol biosynthesis | 66 | ko00945 |
| Amino acid metabolism | Valine, leucine and isoleucine biosynthesis | 65 | ko00290 |
| Metabolism of cofactors and vitamins | Folate biosynthesis | 59 | ko00790 |
| Metabolism of cofactors and vitamins | One carbon pool by folate | 56 | ko00670 |
| Lipid metabolism | Cutin, suberine and wax biosynthesis | 51 | ko00073 |
| Lipid metabolism | Arachidonic acid metabolism | 50 | ko00590 |
| Metabolism of cofactors and vitamins | Nicotinate and nicotinamide metabolism | 49 | ko00760 |
| Lipid metabolism | Linoleic acid metabolism | 48 | ko00591 |
| Metabolism of cofactors and vitamins | Biotin metabolism | 47 | ko00780 |
| Metabolism of terpenoids and polyketides | Zeatin biosynthesis | 45 | ko00908 |
| Lipid metabolism | Synthesis and degradation of ketone bodies | 43 | ko00072 |
| Metabolism of terpenoids and polyketides | Limonene and pinene degradation | 43 | ko00903 |
| Metabolism of other amino acids | Taurine and hypotaurine metabolism | 42 | ko00430 |
| Glycan biosynthesis and metabolism | Glycosphingolipid biosynthesis - globo and isoglobo series | 42 | ko00603 |
| Glycan biosynthesis and metabolism | Other glycan degradation | 39 | ko00511 |
| Glycan biosynthesis and metabolism | Glycosylphosphatidylinositol(GPI)-anchor biosynthesis | 39 | ko00563 |
| Biosynthesis of other secondary metabolites | Monobactam biosynthesis | 32 | ko00261 |
| Amino acid metabolism | Lysine biosynthesis | 32 | ko00300 |
| Folding, sorting and degradation | Sulfur relay system | 32 | ko04122 |
| Glycan biosynthesis and metabolism | Glycosaminoglycan degradation | 31 | ko00531 |
| Metabolism of cofactors and vitamins | Vitamin B6 metabolism | 31 | ko00750 |
| Metabolism of cofactors and vitamins | Riboflavin metabolism | 30 | ko00740 |
| Biosynthesis of other secondary metabolites | Caffeine metabolism | 28 | ko00232 |
| Glycan biosynthesis and metabolism | Other types of O-glycan biosynthesis | 28 | ko00514 |
| Carbohydrate metabolism | C5-Branched dibasic acid metabolism | 28 | ko00660 |
| Biosynthesis of other secondary metabolites | Glucosinolate biosynthesis | 28 | ko00966 |
| Metabolism of terpenoids and polyketides | Monoterpenoid biosynthesis | 22 | ko00902 |
| Metabolism of terpenoids and polyketides | Brassinosteroid biosynthesis | 22 | ko00905 |
| Metabolism of terpenoids and polyketides | Sesquiterpenoid and triterpenoid biosynthesis | 22 | ko00909 |
| Metabolism of terpenoids and polyketides | Diterpenoid biosynthesis | 21 | ko00904 |
| Replication and repair | Non-homologous end-joining | 16 | ko03450 |
| Glycan biosynthesis and metabolism | Glycosphingolipid biosynthesis - ganglio series | 13 | ko00604 |
| Biosynthesis of other secondary metabolites | Betalain biosynthesis | 12 | ko00965 |
| Biosynthesis of other secondary metabolites | Aflatoxin biosynthesis | 9 | ko00254 |
| Metabolism of other amino acids | Phosphonate and phosphinate metabolism | 9 | ko00440 |
| Biosynthesis of other secondary metabolites | Benzoxazinoid biosynthesis | 8 | ko00402 |
| Metabolism of cofactors and vitamins | Lipoic acid metabolism | 8 | ko00785 |
| Biosynthesis of other secondary metabolites | Flavone and flavonol biosynthesis | 8 | ko00944 |
| Biosynthesis of other secondary metabolites | Indole alkaloid biosynthesis | 7 | ko00901 |
| Glycan biosynthesis and metabolism | Mannose type O-glycan biosyntheis | 6 | ko00515 |
| Biosynthesis of other secondary metabolites | Anthocyanin biosynthesis | 4 | ko00942 |
| Biosynthesis of other secondary metabolites | Isoflavonoid biosynthesis | 3 | ko00943 |
| Biosynthesis of other secondary metabolites | Biosynthesis of various secondary metabolites - part 2 | 3 | ko00998 |
| Biosynthesis of other secondary metabolites | Carbapenem biosynthesis | 1 | ko00332 |
| Metabolism of terpenoids and polyketides | Polyketide sugar unit biosynthesis | 1 | ko00523 |
| Glycan biosynthesis and metabolism | Glycosphingolipid biosynthesis - lacto and neolacto series | 1 | ko00601 |

**Table S7: Top 50 GO ID of CK vs T and their p values.**

| GO ID | Description | CK-vs-T (1018) | All (26093) | P value | p. adjust |
| --- | --- | --- | --- | --- | --- |
| GO:0001071 | nucleic acid binding transcription factor activity | 90 | 929 | 1.79E-15 | 6.06E-13 |
| GO:0003677 | DNA binding | 58 | 727 | 2.36E-07 | 3.99E-05 |
| GO:0004672 | protein kinase activity | 117 | 1923 | 1.00E-06 | 0.000113071 |
| GO:0016773 | phosphotransferase activity, alcohol group as acceptor | 120 | 2037 | 3.51E-06 | 0.000296785 |
| GO:0008194 | UDP-glycosyltransferase activity | 29 | 326 | 3.55E-05 | 0.002400432 |
| GO:0016301 | kinase activity | 141 | 2627 | 5.51E-05 | 0.002758917 |
| GO:0016758 | transferase activity, transferring hexosyl groups | 43 | 584 | 5.71E-05 | 0.002758917 |
| GO:0016757 | transferase activity, transferring glycosyl groups | 60 | 956 | 0.000215 | 0.009088584 |
| GO:0000975 | regulatory region DNA binding | 17 | 170 | 0.00037 | 0.012514891 |
| GO:0001067 | regulatory region nucleic acid binding | 17 | 170 | 0.00037 | 0.012514891 |
| GO:0016772 | transferase activity, transferring phosphorus-containing groups | 184 | 3750 | 0.00049 | 0.015049315 |
| GO:0008378 | galactosyltransferase activity | 8 | 59 | 0.001983 | 0.055866779 |
| GO:0016682 | oxidoreductase activity, acting on diphenols and related substances as donors, oxygen as acceptor | 9 | 79 | 0.003604 | 0.093711546 |
| GO:0016679 | oxidoreductase activity, acting on diphenols and related substances as donors | 9 | 83 | 0.005017 | 0.121120219 |
| GO:0035250 | UDP-galactosyltransferase activity | 5 | 31 | 0.006538 | 0.140466077 |
| GO:0016798 | hydrolase activity, acting on glycosyl bonds | 36 | 596 | 0.006721 | 0.140466077 |
| GO:0004721 | phosphoprotein phosphatase activity | 17 | 224 | 0.007065 | 0.140466077 |
| GO:0071617 | lysophospholipid acyltransferase activity | 2 | 4 | 0.008657 | 0.162563946 |
| GO:0004553 | hydrolase activity, hydrolyzing O-glycosyl compounds | 28 | 449 | 0.010484 | 0.186510416 |
| GO:0016160 | amylase activity | 4 | 27 | 0.019771 | 0.334128885 |
| GO:0008422 | beta-glucosidase activity | 6 | 56 | 0.021492 | 0.336020795 |
| GO:0015020 | glucuronosyltransferase activity | 4 | 28 | 0.022372 | 0.336020795 |
| GO:0016740 | transferase activity | 307 | 7137 | 0.022865 | 0.336020795 |
| GO:0016791 | phosphatase activity | 26 | 440 | 0.024339 | 0.342778798 |
| GO:0004857 | enzyme inhibitor activity | 7 | 75 | 0.026907 | 0.363785882 |
| GO:0016857 | racemase and epimerase activity, acting on carbohydrates and derivatives | 6 | 61 | 0.031264 | 0.406427217 |
| GO:0016209 | antioxidant activity | 17 | 273 | 0.040019 | 0.497340581 |
| GO:0030414 | peptidase inhibitor activity | 5 | 49 | 0.041473 | 0.497340581 |
| GO:0042578 | phosphoric ester hydrolase activity | 30 | 551 | 0.042671 | 0.497340581 |
| GO:0005261 | cation channel activity | 5 | 50 | 0.04466 | 0.503173893 |
| GO:0046906 | tetrapyrrole binding | 34 | 645 | 0.047768 | 0.52082692 |
| GO:0061134 | peptidase regulator activity | 5 | 52 | 0.051463 | 0.5435761 |
| GO:0010178 | IAA-amino acid conjugate hydrolase activity | 2 | 10 | 0.055583 | 0.569307677 |
| GO:0004866 | endopeptidase inhibitor activity | 4 | 38 | 0.059588 | 0.592374605 |
| GO:0046527 | glucosyltransferase activity | 16 | 269 | 0.06322 | 0.603724688 |
| GO:0019187 | beta-1,4-mannosyltransferase activity | 3 | 24 | 0.065196 | 0.603724688 |
| GO:0016298 | lipase activity | 8 | 110 | 0.066088 | 0.603724688 |
| GO:0015926 | glucosidase activity | 8 | 112 | 0.071811 | 0.638593096 |
| GO:0061135 | endopeptidase regulator activity | 4 | 41 | 0.074729 | 0.638593096 |
| GO:0035251 | UDP-glucosyltransferase activity | 10 | 153 | 0.077136 | 0.638593096 |
| GO:0016597 | amino acid binding | 2 | 12 | 0.077462 | 0.638593096 |
| GO:0004620 | phospholipase activity | 7 | 96 | 0.081412 | 0.639937174 |
| GO:0052689 | carboxylic ester hydrolase activity | 7 | 96 | 0.081412 | 0.639937174 |
| GO:0008374 | O-acyltransferase activity | 5 | 61 | 0.089105 | 0.65579573 |
| GO:0050734 | hydroxycinnamoyltransferase activity | 2 | 13 | 0.08925 | 0.65579573 |
| GO:0050737 | O-hydroxycinnamoyltransferase activity | 2 | 13 | 0.08925 | 0.65579573 |
| GO:0070717 | poly-purine tract binding | 3 | 28 | 0.094276 | 0.66386181 |
| GO:1902936 | phosphatidylinositol bisphosphate binding | 3 | 28 | 0.094276 | 0.66386181 |
| GO:0008514 | organic anion transmembrane transporter activity | 12 | 203 | 0.101086 | 0.68291803 |
| GO:0046923 | ER retention sequence binding | 2 | 14 | 0.101523 | 0.68291803 |

**Table S8: Details of top 100 GO Id and up and down regulated DEGs of CK vs T.**

| GO ID and description | num | q value | per | ratio | class | P value | Q value | Up | Down |
| --- | --- | --- | --- | --- | --- | --- | --- | --- | --- |
| GO:0010243 response to organonitrogen compound | 65 | 9.32E-14 | 5.672 | 0.129 | Biological Process | 9.90E-17 | 9.32E-14 | 7 | 58 |
| GO:0001101 response to acid chemical | 186 | 9.32E-14 | 16.23 | 0.074 | Biological Process | 1.57E-16 | 9.32E-14 | 31 | 155 |
| GO:0001071 nucleic acid binding transcription factor activity | 90 | 6.06E-13 | 8.841 | 0.097 | Molecular Function | 1.79E-15 | 6.06E-13 | 16 | 74 |
| GO:0007165 signal transduction | 199 | 1.36E-12 | 17.365 | 0.07 | Biological Process | 5.17E-15 | 1.36E-12 | 27 | 172 |
| GO:0023052 signaling | 199 | 1.36E-12 | 17.365 | 0.069 | Biological Process | 5.74E-15 | 1.36E-12 | 27 | 172 |
| GO:0044700 single organism signaling | 199 | 1.36E-12 | 17.365 | 0.069 | Biological Process | 5.74E-15 | 1.36E-12 | 27 | 172 |
| GO:0007154 cell communication | 228 | 6.02E-11 | 19.895 | 0.064 | Biological Process | 3.05E-13 | 6.02E-11 | 29 | 199 |
| GO:0009719 response to endogenous stimulus | 202 | 7.04E-11 | 17.627 | 0.066 | Biological Process | 4.17E-13 | 7.04E-11 | 27 | 175 |
| GO:1901698 response to nitrogen compound | 69 | 6.76E-09 | 6.021 | 0.095 | Biological Process | 4.57E-11 | 6.76E-09 | 8 | 61 |
| GO:0065007 biological regulation | 477 | 1.63E-08 | 41.623 | 0.051 | Biological Process | 1.24E-10 | 1.63E-08 | 85 | 392 |
| GO:0050896 response to stimulus | 596 | 3.46E-08 | 52.007 | 0.049 | Biological Process | 2.92E-10 | 3.46E-08 | 103 | 493 |
| GO:0050789 regulation of biological process | 433 | 6.89E-08 | 37.784 | 0.051 | Biological Process | 6.40E-10 | 6.89E-08 | 77 | 356 |
| GO:0050794 regulation of cellular process | 307 | 2.71E-06 | 26.789 | 0.053 | Biological Process | 2.75E-08 | 2.71E-06 | 54 | 253 |
| GO:0009755 hormone-mediated signaling pathway | 101 | 6.34E-06 | 8.813 | 0.069 | Biological Process | 7.78E-08 | 6.34E-06 | 13 | 88 |
| GO:0032870 cellular response to hormone stimulus | 101 | 6.34E-06 | 8.813 | 0.069 | Biological Process | 8.04E-08 | 6.34E-06 | 13 | 88 |
| GO:0071495 cellular response to endogenous stimulus | 101 | 6.34E-06 | 8.813 | 0.069 | Biological Process | 8.04E-08 | 6.34E-06 | 13 | 88 |
| GO:0006950 response to stress | 391 | 1.02E-05 | 34.119 | 0.05 | Biological Process | 1.38E-07 | 1.02E-05 | 61 | 330 |
| GO:0006972 hyperosmotic response | 35 | 1.26E-05 | 3.054 | 0.106 | Biological Process | 1.81E-07 | 1.26E-05 | 5 | 30 |
| GO:0003677 DNA binding | 58 | 3.99E-05 | 5.697 | 0.08 | Molecular Function | 2.36E-07 | 3.99E-05 | 4 | 54 |
| GO:0009620 response to fungus | 61 | 2.48E-05 | 5.323 | 0.079 | Biological Process | 3.77E-07 | 2.48E-05 | 15 | 46 |
| GO:0043207 response to external biotic stimulus | 164 | 2.99E-05 | 14.311 | 0.059 | Biological Process | 4.80E-07 | 2.99E-05 | 34 | 130 |
| GO:0010033 response to organic substance | 259 | 3.66E-05 | 22.6 | 0.053 | Biological Process | 6.19E-07 | 3.66E-05 | 34 | 225 |
| GO:0051716 cellular response to stimulus | 247 | 4.20E-05 | 21.553 | 0.053 | Biological Process | 7.45E-07 | 4.20E-05 | 31 | 216 |
| GO:0004672 protein kinase activity | 117 | 0.000113 | 11.493 | 0.061 | Molecular Function | 1.00E-06 | 0.000113 | 25 | 92 |
| GO:0009607 response to biotic stimulus | 164 | 7.35E-05 | 14.311 | 0.058 | Biological Process | 1.38E-06 | 7.35E-05 | 34 | 130 |
| GO:0051707 response to other organism | 159 | 7.35E-05 | 13.874 | 0.058 | Biological Process | 1.43E-06 | 7.35E-05 | 31 | 128 |
| GO:0071310 cellular response to organic substance | 122 | 8.41E-05 | 10.646 | 0.061 | Biological Process | 1.71E-06 | 8.41E-05 | 18 | 104 |
| GO:0042221 response to chemical | 374 | 0.000101 | 32.635 | 0.049 | Biological Process | 2.14E-06 | 0.000101 | 52 | 322 |
| GO:0016773 phosphotransferase activity, alcohol group as acceptor | 120 | 0.000297 | 11.788 | 0.059 | Molecular Function | 3.51E-06 | 0.000297 | 27 | 93 |
| GO:0006952 defense response | 107 | 0.000174 | 9.337 | 0.062 | Biological Process | 3.83E-06 | 0.000174 | 15 | 92 |
| GO:0070887 cellular response to chemical stimulus | 123 | 0.000218 | 10.733 | 0.06 | Biological Process | 4.98E-06 | 0.000218 | 18 | 105 |
| GO:0009725 response to hormone | 159 | 0.000235 | 13.874 | 0.057 | Biological Process | 5.55E-06 | 0.000235 | 24 | 135 |
| GO:0002252 immune effector process | 24 | 0.000247 | 2.094 | 0.112 | Biological Process | 6.05E-06 | 0.000247 | 5 | 19 |
| GO:0008219 cell death | 61 | 0.0003 | 5.323 | 0.072 | Biological Process | 7.62E-06 | 0.0003 | 9 | 52 |
| GO:0009605 response to external stimulus | 206 | 0.000312 | 17.976 | 0.053 | Biological Process | 8.16E-06 | 0.000312 | 38 | 168 |
| GO:0012501 programmed cell death | 58 | 0.00033 | 5.061 | 0.073 | Biological Process | 8.94E-06 | 0.00033 | 9 | 49 |
| GO:0043067 regulation of programmed cell death | 55 | 0.000401 | 4.799 | 0.074 | Biological Process | 1.12E-05 | 0.000401 | 8 | 47 |
| GO:0010941 regulation of cell death | 55 | 0.000405 | 4.799 | 0.074 | Biological Process | 1.16E-05 | 0.000405 | 8 | 47 |
| GO:0016265 death | 65 | 0.000409 | 5.672 | 0.07 | Biological Process | 1.21E-05 | 0.000409 | 9 | 56 |
| GO:0009624 response to nematode | 5 | 0.000725 | 0.436 | 0.5 | Biological Process | 2.20E-05 | 0.000725 | 2 | 3 |
| GO:0000160 phosphorelay signal transduction system | 27 | 0.000799 | 2.356 | 0.097 | Biological Process | 2.50E-05 | 0.000799 | 6 | 21 |
| GO:0001906 cell killing | 8 | 0.000914 | 0.698 | 0.25 | Biological Process | 2.94E-05 | 0.000914 | 8 | 0 |
| GO:0008194 UDP-glycosyltransferase activity | 29 | 0.0024 | 2.849 | 0.089 | Molecular Function | 3.55E-05 | 0.0024 | 2 | 27 |
| GO:0016301 kinase activity | 141 | 0.002759 | 13.851 | 0.054 | Molecular Function | 5.51E-05 | 0.002759 | 33 | 108 |
| GO:0016758 transferase activity, transferring hexosyl groups | 43 | 0.002759 | 4.224 | 0.074 | Molecular Function | 5.71E-05 | 0.002759 | 5 | 38 |
| GO:0051704 multi-organism process | 164 | 0.00175 | 14.311 | 0.054 | Biological Process | 5.77E-05 | 0.00175 | 32 | 132 |
| GO:0035556 intracellular signal transduction | 57 | 0.00298 | 4.974 | 0.068 | Biological Process | 0.000101 | 0.00298 | 9 | 48 |
| GO:0016757 transferase activity, transferring glycosyl groups | 60 | 0.009089 | 5.894 | 0.063 | Molecular Function | 0.000215 | 0.009089 | 10 | 50 |
| GO:0002238 response to molecule of fungal origin | 3 | 0.007167 | 0.262 | 0.75 | Biological Process | 0.000251 | 0.007167 | 1 | 2 |
| GO:0002376 immune system process | 73 | 0.007167 | 6.37 | 0.061 | Biological Process | 0.000254 | 0.007167 | 11 | 62 |
| GO:0009832 plant-type cell wall biogenesis | 12 | 0.008585 | 1.047 | 0.13 | Biological Process | 0.000312 | 0.008585 | 6 | 6 |
| GO:0000975 regulatory region DNA binding | 17 | 0.012515 | 1.67 | 0.1 | Molecular Function | 0.00037 | 0.012515 | 2 | 15 |
| GO:0001067 regulatory region nucleic acid binding | 17 | 0.012515 | 1.67 | 0.1 | Molecular Function | 0.00037 | 0.012515 | 2 | 15 |
| GO:0016772 transferase activity, transferring phosphorus-containing groups | 184 | 0.015049 | 18.075 | 0.049 | Molecular Function | 0.00049 | 0.015049 | 47 | 137 |
| GO:0010104 regulation of ethylene-activated signaling pathway | 6 | 0.015953 | 0.524 | 0.222 | Biological Process | 0.000593 | 0.015953 | 0 | 6 |
| GO:0080134 regulation of response to stress | 17 | 0.022879 | 1.483 | 0.096 | Biological Process | 0.00087 | 0.022879 | 2 | 15 |
| GO:0071229 cellular response to acid chemical | 52 | 0.034463 | 4.538 | 0.062 | Biological Process | 0.00134 | 0.034463 | 6 | 46 |
| GO:0071669 plant-type cell wall organization or biogenesis | 32 | 0.035367 | 2.792 | 0.071 | Biological Process | 0.001405 | 0.035367 | 11 | 21 |
| GO:0031347 regulation of defense response | 16 | 0.03721 | 1.396 | 0.094 | Biological Process | 0.00151 | 0.03721 | 2 | 14 |
| GO:0002218 activation of innate immune response | 10 | 0.04233 | 0.873 | 0.12 | Biological Process | 0.001789 | 0.04233 | 1 | 9 |
| GO:0002253 activation of immune response | 10 | 0.04233 | 0.873 | 0.12 | Biological Process | 0.001789 | 0.04233 | 1 | 9 |
| GO:0008378 galactosyltransferase activity | 8 | 0.055867 | 0.786 | 0.136 | Molecular Function | 0.001983 | 0.055867 | 0 | 8 |
| GO:0002757 immune response-activating signal transduction | 3 | 0.044366 | 0.262 | 0.429 | Biological Process | 0.002003 | 0.044366 | 0 | 3 |
| GO:0002758 innate immune response-activating signal transduction | 3 | 0.044366 | 0.262 | 0.429 | Biological Process | 0.002003 | 0.044366 | 0 | 3 |
| GO:0002764 immune response-regulating signaling pathway | 3 | 0.044366 | 0.262 | 0.429 | Biological Process | 0.002003 | 0.044366 | 0 | 3 |
| GO:0009891 positive regulation of biosynthetic process | 18 | 0.044366 | 1.571 | 0.086 | Biological Process | 0.002025 | 0.044366 | 5 | 13 |
| GO:0015833 peptide transport | 12 | 0.049257 | 1.047 | 0.104 | Biological Process | 0.00229 | 0.049257 | 3 | 9 |
| GO:0030054 cell junction | 125 | 0.199372 | 13.843 | 0.046 | Cellular Component | 0.002378 | 0.199372 | 11 | 114 |
| GO:0010468 regulation of gene expression | 171 | 0.050533 | 14.921 | 0.049 | Biological Process | 0.002392 | 0.050533 | 27 | 144 |
| GO:0070297 regulation of phosphorelay signal transduction system | 6 | 0.05958 | 0.524 | 0.167 | Biological Process | 0.002871 | 0.05958 | 0 | 6 |
| GO:0042546 cell wall biogenesis | 25 | 0.063212 | 2.182 | 0.073 | Biological Process | 0.003099 | 0.063212 | 8 | 17 |
| GO:0005911 cell-cell junction | 124 | 0.199372 | 13.732 | 0.045 | Cellular Component | 0.003175 | 0.199372 | 11 | 113 |
| GO:0060255 regulation of macromolecule metabolic process | 190 | 0.063695 | 16.579 | 0.048 | Biological Process | 0.003177 | 0.063695 | 33 | 157 |
| GO:0009308 amine metabolic process | 27 | 0.065681 | 2.356 | 0.071 | Biological Process | 0.003331 | 0.065681 | 9 | 18 |
| GO:0016682 oxidoreductase activity, acting on diphenols and related substances as donors, oxygen as acceptor | 9 | 0.093712 | 0.884 | 0.114 | Molecular Function | 0.003604 | 0.093712 | 1 | 8 |
| GO:0019932 second-messenger-mediated signaling | 15 | 0.070264 | 1.309 | 0.088 | Biological Process | 0.003667 | 0.070264 | 1 | 14 |
| GO:0005618 cell wall | 34 | 0.199372 | 3.765 | 0.058 | Cellular Component | 0.003786 | 0.199372 | 11 | 23 |
| GO:0009690 cytokinin metabolic process | 6 | 0.070264 | 0.524 | 0.158 | Biological Process | 0.003801 | 0.070264 | 3 | 3 |
| GO:0009873 ethylene-activated signaling pathway | 6 | 0.070264 | 0.524 | 0.158 | Biological Process | 0.003801 | 0.070264 | 0 | 6 |
| GO:0071369 cellular response to ethylene stimulus | 6 | 0.070264 | 0.524 | 0.158 | Biological Process | 0.003801 | 0.070264 | 0 | 6 |
| GO:0042886 amide transport | 12 | 0.076879 | 1.047 | 0.097 | Biological Process | 0.004266 | 0.076879 | 3 | 9 |
| GO:0002684 positive regulation of immune system process | 10 | 0.076879 | 0.873 | 0.106 | Biological Process | 0.004484 | 0.076879 | 1 | 9 |
| GO:0031349 positive regulation of defense response | 10 | 0.076879 | 0.873 | 0.106 | Biological Process | 0.004484 | 0.076879 | 1 | 9 |
| GO:0045089 positive regulation of innate immune response | 10 | 0.076879 | 0.873 | 0.106 | Biological Process | 0.004484 | 0.076879 | 1 | 9 |
| GO:0050778 positive regulation of immune response | 10 | 0.076879 | 0.873 | 0.106 | Biological Process | 0.004484 | 0.076879 | 1 | 9 |
| GO:0018871 1-aminocyclopropane-1-carboxylate metabolic process | 2 | 0.077895 | 0.175 | 0.667 | Biological Process | 0.004706 | 0.077895 | 0 | 2 |
| GO:0015849 organic acid transport | 35 | 0.077895 | 3.054 | 0.064 | Biological Process | 0.004741 | 0.077895 | 8 | 27 |
| GO:0046942 carboxylic acid transport | 35 | 0.077895 | 3.054 | 0.064 | Biological Process | 0.004741 | 0.077895 | 8 | 27 |
| GO:0016679 oxidoreductase activity, acting on diphenols and related substances as donors | 9 | 0.12112 | 0.884 | 0.108 | Molecular Function | 0.005017 | 0.12112 | 1 | 8 |
| GO:0009751 response to salicylic acid | 30 | 0.088247 | 2.618 | 0.066 | Biological Process | 0.005446 | 0.088247 | 6 | 24 |
| GO:0009694 jasmonic acid metabolic process | 24 | 0.091059 | 2.094 | 0.071 | Biological Process | 0.005696 | 0.091059 | 4 | 20 |
| GO:0009692 ethylene metabolic process | 14 | 0.091483 | 1.222 | 0.086 | Biological Process | 0.005877 | 0.091483 | 1 | 13 |
| GO:0043449 cellular alkene metabolic process | 14 | 0.091483 | 1.222 | 0.086 | Biological Process | 0.005877 | 0.091483 | 1 | 13 |
| GO:1900673 olefin metabolic process | 14 | 0.095235 | 1.222 | 0.086 | Biological Process | 0.006199 | 0.095235 | 1 | 13 |
| GO:0035250 UDP-galactosyltransferase activity | 5 | 0.140466 | 0.491 | 0.161 | Molecular Function | 0.006538 | 0.140466 | 0 | 5 |
| GO:0016310 phosphorylation | 55 | 0.100033 | 4.799 | 0.057 | Biological Process | 0.006596 | 0.100033 | 13 | 42 |
| GO:0016798 hydrolase activity, acting on glycosyl bonds | 36 | 0.140466 | 3.536 | 0.06 | Molecular Function | 0.006721 | 0.140466 | 18 | 18 |
| GO:0004721 phosphoprotein phosphatase activity | 17 | 0.140466 | 1.67 | 0.076 | Molecular Function | 0.007065 | 0.140466 | 2 | 15 |
| GO:0034703 cation channel complex | 2 | 0.229685 | 0.221 | 0.5 | Cellular Component | 0.007269 | 0.229685 | 0 | 2 |
| GO:0034705 potassium channel complex | 2 | 0.229685 | 0.221 | 0.5 | Cellular Component | 0.007269 | 0.229685 | 0 | 2 |

**Table S9: Details of unigene, GO id and log2 (fc) of CK vs T of plant hormones.**

| GO ID and pathways | Id | Description | Symbol | CK_mean_rpkm | T_mean_rpkm | log2(fc) | P Value | FDR |
| --- | --- | --- | --- | --- | --- | --- | --- | --- |
| GO:0018871 ACC | Unigene0094032 | 1-aminocyclopropane-1-carboxylate synthase 1 [Sorghum bicolor] | ACC1 | 1.326667 | 0.113333 | -3.54916 | 0.00000 | 3.76E-06 |
| GO:0018871 ACC | Unigene0026276 | ACC synthase 2 [Saccharum hybrid cultivar SP80-3280] | ACS7 | 45.59333 | 1.12 | -5.34725 | 0.00000 | 5.58E-68 |
| GO:0009690 Cytokinin | Unigene0050652 | adenylate isopentenyltransferase 5, chloroplastic [Sorghum bicolor] | IPT5 | 8.31 | 17.81667 | 1.100307 | 0.00014 | 0.003674 |
| GO:0009690 Cytokinin | Unigene0004485 | cytokinin dehydrogenase 5 [Sorghum bicolor] | CKX5 | 11.72 | 1.503333 | -2.96274 | 0.00000 | 8.83E-15 |
| GO:0009690 Cytokinin | Unigene0066169 | Cytokinin oxidase 2 [Saccharum officinarum] | CKX4 | 1.72 | 5.43 | 1.658544 | 0.00000 | 5.39E-07 |
| GO:0009690 Cytokinin | Unigene0054972 | cytokinin riboside 5'-monophosphate phosphoribohydrolase LOG [Panicum hallii] | LOG | 3.063333 | 1.273333 | -1.26649 | 0.00052 | 0.011261 |
| GO:0009690 Cytokinin | Unigene0050340 | probable cytokinin riboside 5'-monophosphate phosphoribohydrolase LOGL1 [Sorghum bicolor] | LOGL1 | 7.373333 | 16.43 | 1.155944 | 0.00033 | 0.007572 |
| GO:0009690 Cytokinin | Unigene0107100 | probable cytokinin riboside 5'-monophosphate phosphoribohydrolase LOGL10 [Sorghum bicolor] | LOGL10 | 7.306667 | 2.363333 | -1.62839 | 0.00090 | 0.018073 |
| GO:0009692 Ethylene | Unigene0022448 | 9-cis-epoxycarotenoid dioxygenase 1, chloroplastic [Dichanthelium oligosanthes] | -- | 14.47 | 3.693333 | -1.97007 | 2.43E-06 | 9.51E-05 |
| GO:0009692 Ethylene | Unigene0011984 | AP2 domain-containing protein [Sorghum bicolor] | ERF4 | 227.5333 | 104.1467 | -1.12746 | 5.72E-08 | 2.88E-06 |
| GO:0009692 Ethylene | Unigene0002370 | ethylene-responsive transcription factor 11 [Sorghum bicolor] | ERF4 | 152.68 | 43.56 | -1.80944 | 4.63E-10 | 3.09E-08 |
| GO:0009692 Ethylene | Unigene0018212 | ethylene-responsive transcription factor 11-like [Panicum miliaceum] | ERF3 | 291.1467 | 62.11 | -2.22885 | 4.38E-15 | 4.47E-13 |
| GO:0009692 Ethylene | Unigene0010593 | ethylene-responsive transcription factor 3 [Zea mays] | ERF4 | 22.29 | 4.783333 | -2.22031 | 1.70E-07 | 7.97E-06 |
| GO:0009692 Ethylene | Unigene0010528 | ethylene-responsive transcription factor 3-like [Sorghum bicolor] | ERF3 | 281.3633 | 86.69667 | -1.69839 | 6.79E-08 | 3.39E-06 |
| GO:0009692 Ethylene | Unigene0011983 | ethylene-responsive transcription factor 8 [Sorghum bicolor] | ERF4 | 386.7167 | 121.15 | -1.67448 | 4.77E-07 | 2.09E-05 |
| GO:0009692 Ethylene | Unigene0079684 | ethylene-responsive transcription factor ERF109 [Sorghum bicolor] | ERF109 | 131.75 | 1.976667 | -6.05859 | 2.71E-95 | 7.09E-92 |
| GO:0009692 Ethylene | Unigene0080151 | ethylene-responsive transcription factor ERF109 [Sorghum bicolor] | ERF110 | 9.33 | 0.833333 | -3.48491 | 4.21E-08 | 2.15E-06 |
| GO:0009692 Ethylene | Unigene0023853 | HS1-like protein [Saccharum hybrid cultivar R570] | HSPRO2 | 2092.547 | 231.2967 | -3.17744 | 6.60E-47 | 3.41E-44 |
| GO:0009692 Ethylene | Unigene0070005 | mitochondrial uncoupling protein 5 [Saccharum officinarum] | PUMP5 | 557.06 | 65.13667 | -3.09629 | 3.05E-20 | 4.36E-18 |
| GO:0009692 Ethylene | Unigene0001863 | probable calcium-binding protein CML31 [Sorghum bicolor] | CML19 | 21.28667 | 2.016667 | -3.39991 | 4.47E-21 | 6.64E-19 |
| GO:0009692 Ethylene | Unigene0091842 | protein MODIFYING WALL LIGNIN-1 [Ipomoea triloba] | -- | 47.67 | 9.963333 | -2.25838 | 6.27E-17 | 7.39E-15 |
| GO:0009692 Ethylene | Unigene0083071 | aminotransferase ALD1 homolog [Sorghum bicolor] | ALD1 | 4.81 | 17.38 | 1.853319 | 2.01E-05 | 0.000648 |
| GO:0009692 Ethylene | Unigene0010489 | Grx_I1 - glutaredoxin subgroup III [Zea mays] | GRXS9 | 74.07 | 29.38667 | -1.33373 | 3.64E-05 | 0.001096 |
| GO:0009692 Ethylene | Unigene0074573 | monothiol glutaredoxin-S5-like [Panicum hallii] | GRXS5 | 53.60667 | 3.683333 | -3.86333 | 2.16E-08 | 1.15E-06 |
| GO:0009692 Ethylene | Unigene0094032 | 1-aminocyclopropane-1-carboxylate synthase 1 [Sorghum bicolor] | ACC1 | 1.326667 | 0.113333 | -3.54916 | 7.58E-08 | 3.76E-06 |
| GO:0009692 Ethylene | Unigene0026276 | ACC synthase 2 [Saccharum hybrid cultivar SP80-3280] | ACS7 | 45.59333 | 1.12 | -5.34725 | 4.39E-71 | 5.58E-68 |
| GO:0009692 Ethylene | Unigene0011390 | Dihydrofolate reductase [Zea mays] | -- | 40.85333 | 2.553333 | -4 | 1.28E-38 | 4.67E-36 |
| GO:0009692 Ethylene | Unigene0100029 | phospholipase A1-Ibeta2, chloroplastic [Sorghum bicolor] | At4g16820 | 461.05 | 23.34 | -4.30405 | 3.88E-87 | 8.03E-84 |
| GO:0009694 Jasmonic acid | Unigene0046120 | arginine decarboxylase 1 [Sorghum bicolor] | ADC1 | 225.1067 | 82.63333 | -1.44581 | 2.01E-15 | 2.11E-13 |
| GO:0009694 Jasmonic acid | Unigene0056418 | cytosolic sulfotransferase 14 [Setaria italica] | SOT15 | 3.18 | 0.526667 | -2.59406 | 1.27E-07 | 6.07E-06 |
| GO:0009694 Jasmonic acid | Unigene0021737 | fructose-bisphosphate aldolase 1 [Arabidopsis thaliana] | FBA1 | 4.113333 | 0.396667 | -3.37431 | 3.02E-06 | 0.000116 |
| GO:0009694 Jasmonic acid | Unigene0021736 | fructose-bisphosphate aldolase 2 [Arabidopsis thaliana] | FBA2 | 6.25 | 1.49 | -2.06854 | 0.000127 | 0.003331 |
| GO:0009694 Jasmonic acid | Unigene0092662 | GDP-L-galactose phosphorylase 1 [Arabidopsis thaliana] | VTC2 | 0.816667 | 0.07 | -3.54432 | 0.00118 | 0.02253 |
| GO:0009694 Jasmonic acid | Unigene0006013 | IAA-amino acid hydrolase ILR1-like 2 [Sorghum bicolor] | ILL2 | 3.493333 | 1.24 | -1.49426 | 0.000708 | 0.014652 |
| GO:0009694 Jasmonic acid | Unigene0091011 | Jasmonate O-methyltransferase [Zea mays] | JMT | 2.253333 | 0.8 | -1.49399 | 0.001948 | 0.033824 |
| GO:0009694 Jasmonic acid | Unigene0081654 | probable 2-oxoglutarate-dependent dioxygenase At3g111800 [Sorghum bicolor] | At3g11180 | 8.38 | 20.48333 | 1.289428 | 0.000736 | 0.015126 |
| GO:0009694 Jasmonic acid | Unigene0081655 | probable 2-oxoglutarate-dependent dioxygenase At5g05600 [Sorghum bicolor] | At3g11180 | 9.66 | 22.30667 | 1.20738 | 0.0012 | 0.022855 |
| GO:0009694 Jasmonic acid | Unigene0064447 | probable choline kinase 2 isoform X1 [Panicum hallii] | At1g74320 | 8.15 | 3.576667 | -1.18818 | 1.09E-07 | 5.27E-06 |
| GO:0009694 Jasmonic acid | Unigene0078113 | probable galacturonosyltransferase-like 1 [Sorghum bicolor] | GATL1 | 95.34 | 13.88333 | -2.77973 | 1.15E-31 | 2.97E-29 |
| GO:0009694 Jasmonic acid | Unigene0007290 | probable GTP diphosphokinase RSH2, chloroplastic isoform X1 [Zea mays] | RSH2 | 3.473333 | 1.533333 | -1.17965 | 0.000218 | 0.005306 |
| GO:0009694 Jasmonic acid | Unigene0067451 | probable monogalactosyldiacylglycerol synthase 3, chloroplastic [Sorghum bicolor] | MGD3 | 21.39333 | 7.53 | -1.50644 | 5.45E-09 | 3.17E-07 |
| GO:0009694 Jasmonic acid | Unigene0078189 | probable ribose-5-phosphate isomerase 1 [Sorghum bicolor] | RPI2 | 75.54667 | 23.54333 | -1.68205 | 1.24E-11 | 9.66E-10 |
| GO:0009694 Jasmonic acid | Unigene0024863 | probable serine acetyltransferase 4 [Sorghum bicolor] | SAT4 | 16.53 | 6.563333 | -1.33259 | 2.84E-07 | 1.29E-05 |
| GO:0009694 Jasmonic acid | Unigene0091964 | protein STRICTOSIDINE SYNTHASE-LIKE 5 [Setaria italica] | SSL5 | 12.20333 | 30.37667 | 1.315688 | 0.000805 | 0.01636 |
| GO:0009694 Jasmonic acid | Unigene0001285 | transcription factor MYB4 [Sorghum bicolor] | MYB4 | 10.12667 | 2.63 | -1.94502 | 1.36E-12 | 1.15E-10 |
| GO:0009694 Jasmonic acid | Unigene0099873 | transcription factor MYB41 [Setaria italica] | MYB102 | 9.553333 | 1.323333 | -2.85183 | 1.13E-08 | 6.27E-07 |
| GO:0009694 Jasmonic acid | Unigene0011390 | Dihydrofolate reductase [Zea mays] | -- | 40.85333 | 2.553333 | -4 | 1.28E-38 | 4.67E-36 |
| GO:0009694 Jasmonic acid | Unigene0100029 | phospholipase A1-Ibeta2, chloroplastic [Sorghum bicolor] | At4g16820 | 461.05 | 23.34 | -4.30405 | 3.88E-87 | 8.03E-84 |
| GO:0009694 Jasmonic acid | Unigene0080205 | cytochrome P450 [Saccharum hybrid cultivar R570] | CYP74A1 | 295.56 | 57.96667 | -2.35016 | 3.36E-15 | 3.48E-13 |
| GO:0009694 Jasmonic acid | Unigene0024887 | NAC domain-containing protein 92 [Sorghum bicolor] | NAC079 | 2.143333 | 9.98 | 2.219184 | 3.63E-05 | 0.001094 |
| GO:0009694 Jasmonic acid | Unigene0010489 | Grx_I1 - glutaredoxin subgroup III [Zea mays] | GRXS9 | 74.07 | 29.38667 | -1.33373 | 3.64E-05 | 0.001096 |
| GO:0009694 Jasmonic acid | Unigene0074573 | monothiol glutaredoxin-S5-like [Panicum hallii] | GRXS5 | 53.60667 | 3.683333 | -3.86333 | 2.16E-08 | 1.15E-06 |
| GO:0006558 L-phenylalanine | Unigene0090990 | Arogenate dehydratase 3, chloroplastic [Zea mays] | ADT3 | 29.93333 | 13.07667 | -1.19476 | 1.16E-05 | 0.000395 |
| GO:0006558 L-phenylalanine | Unigene0032060 | Phenylalanine ammonia-lyase [Dichanthelium oligosanthes] | ZB8 | 73.5 | 30.27333 | -1.2797 | 1.34E-14 | 1.31E-12 |
| GO:0006558 L-phenylalanine | Unigene0082953 | phenylalanine ammonia-lyase [Sorghum bicolor] | ZB8 | 142.1633 | 57.16667 | -1.3143 | 4.30E-14 | 4.10E-12 |
| GO:0009751 Salicylic acid | Unigene0016362 | ATPase 3 [Zea mays] | AATP1 | 7.863333 | 21.25 | 1.43425 | 0.00066 | 0.013764 |
| GO:0009751 Salicylic acid | Unigene0010928 | carbonic anhydrase 1 [Arabidopsis thaliana] | BCA1 | 4.016667 | 0.79 | -2.34607 | 0.000137 | 0.003562 |
| GO:0009751 Salicylic acid | Unigene0079426 | exocyst complex component EXO70B1 [Sorghum bicolor] | EXO70B1 | 136.5533 | 50.28 | -1.44141 | 1.24E-21 | 1.89E-19 |
| GO:0009751 Salicylic acid | Unigene0042637 | late embryogenesis abundant protein Lea5-D [Sorghum bicolor] | -- | 24.37333 | 2.733333 | -3.15657 | 8.93E-18 | 1.12E-15 |
| GO:0009751 Salicylic acid | Unigene0021858 | late embryogenis abundant protein 2 [Sorghum bicolor] | -- | 10.05333 | 3.893333 | -1.3686 | 1.99E-05 | 0.000641 |
| GO:0009751 Salicylic acid | Unigene0066102 | LOB domain-containing protein 42 [Sorghum bicolor] | LBD42 | 159.3767 | 43.36 | -1.878 | 0.001734 | 0.030928 |
| GO:0009751 Salicylic acid | Unigene0109255 | MACPF domain-containing protein NSL1 [Sorghum bicolor] | NSL1 | 20.59 | 2.366667 | -3.12102 | 4.36E-24 | 7.57E-22 |
| GO:0009751 Salicylic acid | Unigene0050233 | mitogen-activated protein kinase 4 [Saccharum hybrid cultivar ROC22] | MPK5 | 425.2833 | 55.52333 | -2.93726 | 2.58E-53 | 1.71E-50 |
| GO:0009751 Salicylic acid | Unigene0040118 | mitogen-activated protein kinase 4 [Sorghum bicolor] | MPK4 | 73.35 | 14.34 | -2.35475 | 3.97E-49 | 2.22E-46 |
| GO:0009751 Salicylic acid | Unigene0043677 | Orphans transcription factor [Saccharum spontaneum] | BBX22 | 18.28667 | 6.086667 | -1.58707 | 1.15E-24 | 2.07E-22 |
| GO:0009751 Salicylic acid | Unigene0113415 | patatin-like protein 2 [Sorghum bicolor] | PLP2 | 5.703333 | 0.333333 | -4.09677 | 3.48E-25 | 6.50E-23 |
| GO:0009751 Salicylic acid | Unigene0066387 | photosystem II subunit O-2 [Arabidopsis thaliana] | PSBO2 | 3.006667 | 1.12 | -1.42467 | 0.002303 | 0.038744 |
| GO:0009751 Salicylic acid | Unigene0009358 | polygalacturonase inhibitor [Sorghum bicolor] | PGIP1 | 8.383333 | 24.61333 | 1.553844 | 4.45E-06 | 0.000165 |
| GO:0009751 Salicylic acid | Unigene0009359 | polygalacturonase inhibitor [Sorghum bicolor] | PGIP1 | 8.953333 | 20.99667 | 1.229664 | 2.06E-05 | 0.000661 |
| GO:0009751 Salicylic acid | Unigene0053255 | probable 1-acyl-sn-glycerol-3-phosphate acyltransferase 5 [Sorghum bicolor] | LPAT5 | 10.84667 | 3.443333 | -1.65537 | 3.60E-17 | 4.33E-15 |
| GO:0009751 Salicylic acid | Unigene0000501 | probable E3 ubiquitin-protein ligase XBOS34 [Sorghum bicolor] | XBOS34 | 2.763333 | 1.26 | -1.13299 | 2.49E-06 | 9.71E-05 |
| GO:0009751 Salicylic acid | Unigene0104995 | probable LRR receptor-like serine/threonine-protein kinase At1g74360 [Sorghum bicolor] | At1g74360 | 48.67333 | 16.97667 | -1.51958 | 8.49E-09 | 4.84E-07 |
| GO:0009751 Salicylic acid | Unigene0080658 | protein DOWNY MILDEW RESISTANCE 6 [Sorghum bicolor] | F3H-2 | 4.91 | 2.146667 | -1.19362 | 7.13E-05 | 0.002003 |
| GO:0009751 Salicylic acid | Unigene0022129 | protein LURP-one-related 15-like [Panicum miliaceum] | At5g01750 | 2.516667 | 0.753333 | -1.74015 | 0.002672 | 0.043764 |
| GO:0009751 Salicylic acid | Unigene0028710 | protein MLO [Sorghum bicolor] | MLO-H1 | 1.196667 | 0.163333 | -2.87313 | 5.77E-05 | 0.001658 |
| GO:0009751 Salicylic acid | Unigene0028123 | protein phosphatase 2C 35 [Sorghum bicolor] | XB15 | 29.49 | 3.633333 | -3.02086 | 7.92E-32 | 2.09E-29 |
| GO:0009751 Salicylic acid | Unigene0055235 | PVR3-like protein [Zea mays] | DIR1 | 10.89667 | 25.32667 | 1.21677 | 0.001341 | 0.024926 |
| GO:0009751 Salicylic acid | Unigene0019857 | pyruvate dehydrogenase phosphatase [Salvia splendens] | Os05g0111800 | 6.653333 | 2.463333 | -1.43347 | 3.01E-05 | 0.000924 |
| GO:0009751 Salicylic acid | Unigene0088999 | stem-specific protein TSJT1 [Oryza sativa Japonica Group] | TSJT1 | 0.79 | 0.001 | -9.62571 | 7.16E-05 | 0.002006 |
| GO:0009751 Salicylic acid | Unigene0025688 | transcription factor MYB44 [Sorghum bicolor] | MYB73 | 5.81 | 1.513333 | -1.94081 | 7.46E-06 | 0.000265 |
| GO:0009751 Salicylic acid | Unigene0083071 | aminotransferase ALD1 homolog [Sorghum bicolor] | ALD1 | 4.81 | 17.38 | 1.853319 | 2.01E-05 | 0.000648 |
| GO:0009751 Salicylic acid | Unigene0080205 | cytochrome P450 [Saccharum hybrid cultivar R570] | CYP74A1 | 295.56 | 57.96667 | -2.35016 | 3.36E-15 | 3.48E-13 |
| GO:0009751 Salicylic acid | Unigene0024887 | NAC domain-containing protein 92 [Sorghum bicolor] | NAC079 | 2.143333 | 9.98 | 2.219184 | 3.63E-05 | 0.001094 |
| GO:0009751 Salicylic acid | Unigene0010489 | Grx_I1 - glutaredoxin subgroup III [Zea mays] | GRXS9 | 74.07 | 29.38667 | -1.33373 | 3.64E-05 | 0.001096 |
| GO:0009751 Salicylic acid | Unigene0074573 | monothiol glutaredoxin-S5-like [Panicum hallii] | GRXS5 | 53.60667 | 3.683333 | -3.86333 | 2.16E-08 | 1.15E-06 |

**Table S10: Details of significantly expressed GO ids in CK vs T.**

| **Biological process** | |  |  |  |
| --- | --- | --- | --- | --- |
| GO ID | Description | CK-vs-T (1146) | All (28534) | p value |
| GO:0010243 | response to organonitrogen compound | 65 | 503 | 0.000 |
| GO:0001101 | response to acid chemical | 186 | 2519 | 0.000 |
| GO:0007165 | signal transduction | 199 | 2861 | 0.000 |
| GO:0023052 | signaling | 199 | 2864 | 0.000 |
| GO:0044700 | single organism signaling | 199 | 2864 | 0.000 |
| GO:0007154 | cell communication | 228 | 3566 | 0.000 |
| GO:0009719 | response to endogenous stimulus | 202 | 3053 | 0.000 |
| GO:1901698 | response to nitrogen compound | 69 | 729 | 0.000 |
| GO:0065007 | biological regulation | 477 | 9366 | 0.000 |
| GO:0050896 | response to stimulus | 596 | 12281 | 0.000 |
| GO:0050789 | regulation of biological process | 433 | 8432 | 0.000 |
| GO:0050794 | regulation of cellular process | 307 | 5769 | 0.000 |
| GO:0009755 | hormone-mediated signaling pathway | 101 | 1463 | 0.000 |
| GO:0032870 | cellular response to hormone stimulus | 101 | 1464 | 0.000 |
| GO:0071495 | cellular response to endogenous stimulus | 101 | 1464 | 0.000 |
| GO:0006950 | response to stress | 391 | 7792 | 0.000 |
| GO:0006972 | hyperosmotic response | 35 | 329 | 0.000 |
| GO:0009620 | response to fungus | 61 | 768 | 0.000 |
| GO:0043207 | response to external biotic stimulus | 164 | 2801 | 0.000 |
| GO:0010033 | response to organic substance | 259 | 4876 | 0.000 |
| GO:0051716 | cellular response to stimulus | 247 | 4620 | 0.000 |
| GO:0009607 | response to biotic stimulus | 164 | 2850 | 0.000 |
| GO:0051707 | response to other organism | 159 | 2746 | 0.000 |
| GO:0071310 | cellular response to organic substance | 122 | 1986 | 0.000 |
| GO:0042221 | response to chemical | 374 | 7589 | 0.000 |
| GO:0006952 | defense response | 107 | 1714 | 0.000 |
| GO:0070887 | cellular response to chemical stimulus | 123 | 2051 | 0.000 |
| GO:0009725 | response to hormone | 159 | 2812 | 0.000 |
| GO:0002252 | immune effector process | 24 | 214 | 0.000 |
| GO:0008219 | cell death | 61 | 843 | 0.000 |
| GO:0009605 | response to external stimulus | 206 | 3854 | 0.000 |
| GO:0012501 | programmed cell death | 58 | 792 | 0.000 |
| GO:0043067 | regulation of programmed cell death | 55 | 743 | 0.000 |
| GO:0010941 | regulation of cell death | 55 | 744 | 0.000 |
| GO:0016265 | death | 65 | 931 | 0.000 |
| GO:0009624 | response to nematode | 5 | 10 | 0.000 |
| GO:0000160 | phosphorelay signal transduction system | 27 | 279 | 0.000 |
| GO:0001906 | cell killing | 8 | 32 | 0.000 |
| GO:0051704 | multi-organism process | 164 | 3048 | 0.000 |
| GO:0035556 | intracellular signal transduction | 57 | 844 | 0.000 |
| GO:0002238 | response to molecule of fungal origin | 3 | 4 | 0.000 |
| GO:0002376 | immune system process | 73 | 1194 | 0.000 |
| GO:0009832 | plant-type cell wall biogenesis | 12 | 92 | 0.000 |
| GO:0010104 | regulation of ethylene-activated signaling pathway | 6 | 27 | 0.001 |
| GO:0080134 | regulation of response to stress | 17 | 178 | 0.001 |
| GO:0071229 | cellular response to acid chemical | 52 | 837 | 0.001 |
| GO:0071669 | plant-type cell wall organization or biogenesis | 32 | 450 | 0.001 |
| GO:0031347 | regulation of defense response | 16 | 171 | 0.002 |
| GO:0002218 | activation of innate immune response | 10 | 83 | 0.002 |
| GO:0002253 | activation of immune response | 10 | 83 | 0.002 |
| GO:0002757 | immune response-activating signal transduction | 3 | 7 | 0.002 |
| GO:0002758 | innate immune response-activating signal transduction | 3 | 7 | 0.002 |
| GO:0002764 | immune response-regulating signaling pathway | 3 | 7 | 0.002 |
| GO:0009891 | positive regulation of biosynthetic process | 18 | 209 | 0.002 |
| GO:0015833 | peptide transport | 12 | 115 | 0.002 |
| GO:0010468 | regulation of gene expression | 171 | 3465 | 0.002 |
| GO:0070297 | regulation of phosphorelay signal transduction system | 6 | 36 | 0.003 |
| GO:0042546 | cell wall biogenesis | 25 | 342 | 0.003 |
| GO:0060255 | regulation of macromolecule metabolic process | 190 | 3925 | 0.003 |
| GO:0009308 | amine metabolic process | 27 | 381 | 0.003 |
| GO:0019932 | second-messenger-mediated signaling | 15 | 170 | 0.004 |
| GO:0009690 | cytokinin metabolic process | 6 | 38 | 0.004 |
| GO:0009873 | ethylene-activated signaling pathway | 6 | 38 | 0.004 |
| GO:0071369 | cellular response to ethylene stimulus | 6 | 38 | 0.004 |
| GO:0042886 | amide transport | 12 | 124 | 0.004 |
| GO:0002684 | positive regulation of immune system process | 10 | 94 | 0.004 |
| GO:0031349 | positive regulation of defense response | 10 | 94 | 0.004 |
| GO:0045089 | positive regulation of innate immune response | 10 | 94 | 0.004 |
| GO:0050778 | positive regulation of immune response | 10 | 94 | 0.004 |
| GO:0018871 | 1-aminocyclopropane-1-carboxylate metabolic process | 2 | 3 | 0.005 |
| GO:0015849 | organic acid transport | 35 | 546 | 0.005 |
| GO:0046942 | carboxylic acid transport | 35 | 546 | 0.005 |
| GO:0009751 | response to salicylic acid | 30 | 453 | 0.005 |
| GO:0009694 | jasmonic acid metabolic process | 24 | 340 | 0.006 |
| GO:0009692 | ethylene metabolic process | 14 | 162 | 0.006 |
| GO:0043449 | cellular alkene metabolic process | 14 | 162 | 0.006 |
| GO:1900673 | olefin metabolic process | 14 | 163 | 0.006 |
| GO:0016310 | phosphorylation | 55 | 969 | 0.007 |
| GO:0010162 | seed dormancy process | 4 | 20 | 0.007 |
| GO:0022611 | dormancy process | 4 | 20 | 0.007 |
| GO:0015711 | organic anion transport | 37 | 605 | 0.008 |
| GO:0009863 | salicylic acid mediated signaling pathway | 29 | 446 | 0.008 |
| GO:0071446 | cellular response to salicylic acid stimulus | 29 | 446 | 0.008 |
| GO:0055090 | acylglycerol homeostasis | 2 | 4 | 0.009 |
| GO:0019222 | regulation of metabolic process | 221 | 4766 | 0.010 |
| GO:0048583 | regulation of response to stimulus | 31 | 495 | 0.010 |
| GO:0048584 | positive regulation of response to stimulus | 11 | 123 | 0.011 |
| GO:0045088 | regulation of innate immune response | 10 | 107 | 0.011 |
| GO:0050776 | regulation of immune response | 10 | 107 | 0.011 |
| GO:0006820 | anion transport | 61 | 1132 | 0.013 |
| GO:1902531 | regulation of intracellular signal transduction | 7 | 63 | 0.013 |
| GO:0000271 | polysaccharide biosynthetic process | 9 | 94 | 0.013 |
| GO:0032890 | regulation of organic acid transport | 2 | 5 | 0.015 |
| GO:0051952 | regulation of amine transport | 2 | 5 | 0.015 |
| GO:0051955 | regulation of amino acid transport | 2 | 5 | 0.015 |
| GO:0009723 | response to ethylene | 6 | 51 | 0.016 |
| GO:0098542 | defense response to other organism | 44 | 783 | 0.016 |
| GO:0016567 | protein ubiquitination | 9 | 99 | 0.018 |
| GO:0009827 | plant-type cell wall modification | 7 | 69 | 0.021 |
| GO:0015837 | amine transport | 2 | 6 | 0.022 |
| GO:0010431 | seed maturation | 4 | 27 | 0.022 |
| GO:0043086 | negative regulation of catalytic activity | 7 | 71 | 0.024 |
| GO:0009834 | plant-type secondary cell wall biogenesis | 3 | 16 | 0.024 |
| GO:0009267 | cellular response to starvation | 32 | 552 | 0.025 |
| GO:0042594 | response to starvation | 32 | 552 | 0.025 |
| GO:0010466 | negative regulation of peptidase activity | 6 | 59 | 0.031 |
| GO:0052547 | regulation of peptidase activity | 6 | 59 | 0.031 |
| GO:0002682 | regulation of immune system process | 10 | 127 | 0.032 |
| GO:0031669 | cellular response to nutrient levels | 32 | 568 | 0.035 |
| GO:0010565 | regulation of cellular ketone metabolic process | 6 | 61 | 0.035 |
| GO:0045861 | negative regulation of proteolysis | 6 | 61 | 0.035 |
| GO:0010646 | regulation of cell communication | 24 | 402 | 0.036 |
| GO:1901136 | carbohydrate derivative catabolic process | 7 | 78 | 0.037 |
| GO:0006468 | protein phosphorylation | 39 | 720 | 0.037 |
| GO:1901701 | cellular response to oxygen-containing compound | 55 | 1070 | 0.037 |
| GO:0044106 | cellular amine metabolic process | 21 | 343 | 0.038 |
| GO:0016099 | monoterpenoid biosynthetic process | 2 | 8 | 0.038 |
| GO:0003156 | regulation of organ formation | 1 | 1 | 0.040 |
| GO:0006884 | cell volume homeostasis | 1 | 1 | 0.040 |
| GO:0061392 | regulation of transcription from RNA polymerase II promoter in response to osmotic stress | 1 | 1 | 0.040 |
| GO:0071470 | cellular response to osmotic stress | 1 | 1 | 0.040 |
| GO:0051346 | negative regulation of hydrolase activity | 6 | 63 | 0.040 |
| GO:0009966 | regulation of signal transduction | 17 | 266 | 0.041 |
| GO:0023051 | regulation of signaling | 17 | 266 | 0.041 |
| GO:0009625 | response to insect | 7 | 80 | 0.042 |
| GO:0032269 | negative regulation of cellular protein metabolic process | 7 | 80 | 0.042 |
| GO:0051248 | negative regulation of protein metabolic process | 7 | 80 | 0.042 |
| GO:0006576 | cellular biogenic amine metabolic process | 20 | 327 | 0.042 |
| GO:0016051 | carbohydrate biosynthetic process | 10 | 133 | 0.042 |
| GO:0031667 | response to nutrient levels | 32 | 577 | 0.042 |
| GO:0030162 | regulation of proteolysis | 6 | 64 | 0.043 |
| GO:0031668 | cellular response to extracellular stimulus | 32 | 579 | 0.044 |
| GO:0071496 | cellular response to external stimulus | 32 | 579 | 0.044 |
| GO:0071554 | cell wall organization or biogenesis | 68 | 1374 | 0.045 |
| GO:0009845 | seed germination | 12 | 173 | 0.047 |
| GO:0033692 | cellular polysaccharide biosynthetic process | 6 | 66 | 0.049 |
| GO:0009991 | response to extracellular stimulus | 32 | 585 | 0.049 |
| GO:0055081 | anion homeostasis | 5 | 50 | 0.050 |
| **Molecular function** | |  |  |  |
| GO ID | Description | CK-vs-T (1018) | All (26093) | pvalue |
| GO:0001071 | nucleic acid binding transcription factor activity | 90 | 929 | 0.000 |
| GO:0003677 | DNA binding | 58 | 727 | 0.000 |
| GO:0004672 | protein kinase activity | 117 | 1923 | 0.000 |
| GO:0016773 | phosphotransferase activity, alcohol group as acceptor | 120 | 2037 | 0.000 |
| GO:0008194 | UDP-glycosyltransferase activity | 29 | 326 | 0.000 |
| GO:0016301 | kinase activity | 141 | 2627 | 0.000 |
| GO:0016758 | transferase activity, transferring hexosyl groups | 43 | 584 | 0.000 |
| GO:0016757 | transferase activity, transferring glycosyl groups | 60 | 956 | 0.000 |
| GO:0000975 | regulatory region DNA binding | 17 | 170 | 0.000 |
| GO:0001067 | regulatory region nucleic acid binding | 17 | 170 | 0.000 |
| GO:0016772 | transferase activity, transferring phosphorus-containing groups | 184 | 3750 | 0.000 |
| GO:0008378 | galactosyltransferase activity | 8 | 59 | 0.002 |
| GO:0016682 | oxidoreductase activity, acting on diphenols and related substances as donors, oxygen as acceptor | 9 | 79 | 0.004 |
| GO:0016679 | oxidoreductase activity, acting on diphenols and related substances as donors | 9 | 83 | 0.005 |
| GO:0035250 | UDP-galactosyltransferase activity | 5 | 31 | 0.007 |
| GO:0016798 | hydrolase activity, acting on glycosyl bonds | 36 | 596 | 0.007 |
| GO:0004721 | phosphoprotein phosphatase activity | 17 | 224 | 0.007 |
| GO:0071617 | lysophospholipid acyltransferase activity | 2 | 4 | 0.009 |
| GO:0004553 | hydrolase activity, hydrolyzing O-glycosyl compounds | 28 | 449 | 0.010 |
| GO:0016160 | amylase activity | 4 | 27 | 0.020 |
| GO:0008422 | beta-glucosidase activity | 6 | 56 | 0.021 |
| GO:0015020 | glucuronosyltransferase activity | 4 | 28 | 0.022 |
| GO:0016740 | transferase activity | 307 | 7137 | 0.023 |
| GO:0016791 | phosphatase activity | 26 | 440 | 0.024 |
| GO:0004857 | enzyme inhibitor activity | 7 | 75 | 0.027 |
| GO:0016857 | racemase and epimerase activity, acting on carbohydrates and derivatives | 6 | 61 | 0.031 |
| GO:0016209 | antioxidant activity | 17 | 273 | 0.040 |
| GO:0030414 | peptidase inhibitor activity | 5 | 49 | 0.041 |
| GO:0042578 | phosphoric ester hydrolase activity | 30 | 551 | 0.043 |
| GO:0005261 | cation channel activity | 5 | 50 | 0.045 |
| GO:0046906 | tetrapyrrole binding | 34 | 645 | 0.048 |
| **Cell component** | |  |  |  |
| GO ID | Description | CK-vs-T (903) | All (25316) | pvalue |
| GO:0030054 | cell junction | 125 | 2742 | 0.002378 |
| GO:0005911 | cell-cell junction | 124 | 2740 | 0.003175 |
| GO:0005618 | cell wall | 34 | 584 | 0.003786 |
| GO:0034703 | cation channel complex | 2 | 4 | 0.007269 |
| GO:0034705 | potassium channel complex | 2 | 4 | 0.007269 |
| GO:0071944 | cell periphery | 96 | 2118 | 0.008838 |
| GO:0031225 | anchored component of membrane | 9 | 128 | 0.039958 |
| GO:0030312 | external encapsulating structure | 85 | 1985 | 0.044832 |
| GO:0044445 | cytosolic part | 4 | 39 | 0.049317 |

**Table S11: Details of unigene, GO id and log2 (fc) of CK vs T of functional genes of biological process.**

| GO:0071229 Cellular Response to Acid Chemical | | | | |  |  |  |
| --- | --- | --- | --- | --- | --- | --- | --- |
| Unigenes | Description | Symbol | CK_mean_rpkm | T_mean_rpkm | log2(fc) | PValue | FDR |
| Unigene0055093 | 3-ketoacyl-CoA thiolase, peroxisomal-like [Quercus suber] | POT1 | 0.8 | 0.001 | -9.64386 | 0.002531 | 0.041955 |
| Unigene0044549 | ammonium transporter 1-like [Quercus suber] | amt1 | 0.716667 | 0.053333 | -3.74819 | 0.000901 | 0.018012 |
| Unigene0066866 | B2 protein isoform X1 [Setaria italica] | -- | 538.3767 | 57.99667 | -3.21457 | 1.59E-77 | 2.46E-74 |
| Unigene0074970 | calmodulin [Aegilops tauschii subsp. tauschii] [Aegilops tauschii] | CP1 | 26.84667 | 7.336667 | -1.87155 | 7.10E-12 | 5.67E-10 |
| Unigene0086430 | Os01g0256500, partial [Oryza sativa Japonica Group] | -- | 0.886667 | 0.05 | -4.14839 | 0.003151 | 0.049843 |
| Unigene0011676 | phospholipase D alpha 1 [Sorghum bicolor] | PLD1 | 124.2133 | 47.35667 | -1.39118 | 4.35E-21 | 6.48E-19 |
| Unigene0000628 | phospholipase D alpha 2 [Sorghum bicolor] | PLD2 | 14.09667 | 5.983333 | -1.23633 | 0.000858 | 0.017281 |
| Unigene0044945 | phospholipase D family protein isoform X1 [Zea mays] | PLD1 | 6.886667 | 3.13 | -1.13764 | 5.54E-05 | 0.001606 |
| Unigene0071113 | PREDICTED: tubulin beta-8 chain-like, partial [Camelina sativa] | TUBB1 | 0.763333 | 0.001 | -9.57617 | 0.001135 | 0.021811 |
| Unigene0002506 | protein SAR DEFICIENT 1 [Sorghum bicolor] | CBP60B | 108.7933 | 7.603333 | -3.83881 | 1.15E-46 | 5.86E-44 |
| Unigene0018160 | protein TIFY 11b [Setaria italica] | TIFY11F | 4.976667 | 0.156667 | -4.98941 | 1.80E-13 | 1.64E-11 |
| Unigene0017414 | receptor-like protein kinase FERONIA [Sorghum bicolor] | FER | 147.9967 | 15.63 | -3.24317 | 2.43E-60 | 2.12E-57 |
| Unigene0102633 | receptor-like protein kinase FERONIA [Sorghum bicolor] | FER | 85.66333 | 38.09 | -1.16927 | 1.40E-12 | 1.19E-10 |
| Unigene0019726 | SNAP25 ous protein SNAP33 [Zea mays] | SNAP33 | 45.11667 | 9 | -2.32566 | 1.47E-36 | 4.80E-34 |
| Unigene0012891 | WRKY transcription factor WRKY28 [Sorghum bicolor] | WRKY28 | 153.06 | 68.61333 | -1.15754 | 0.000101 | 0.00271 |
| Unigene0048880 | ZEB2-regulated ABC transporter 1-like [Quercus suber] | abcC | 0.953333 | 0.04 | -4.57491 | 6.63E-08 | 3.32E-06 |
| Unigene0007162 | Zinc-finger homeodomain protein 8 [Dichanthelium oligosanthes] | ZHD8 | 1.186667 | 0.05 | -4.56884 | 0.001397 | 0.025799 |
| Unigene0016362 | ATPase 3 [Zea mays] | AATP1 | 7.863333 | 21.25 | 1.43425 | 0.00066 | 0.013764 |
| Unigene0010928 | carbonic anhydrase 1 [Arabidopsis thaliana] | BCA1 | 4.016667 | 0.79 | -2.34607 | 0.000137 | 0.003562 |
| Unigene0079426 | exocyst complex component EXO70B1 [Sorghum bicolor] | EXO70B1 | 136.5533 | 50.28 | -1.44141 | 1.24E-21 | 1.89E-19 |
| Unigene0042637 | late embryogenesis abundant protein Lea5-D [Sorghum bicolor] | -- | 24.37333 | 2.733333 | -3.15657 | 8.93E-18 | 1.12E-15 |
| Unigene0021858 | late embryogenis abundant protein 2 [Sorghum bicolor] | -- | 10.05333 | 3.893333 | -1.3686 | 1.99E-05 | 0.000641 |
| Unigene0066102 | LOB domain-containing protein 42 [Sorghum bicolor] | LBD42 | 159.3767 | 43.36 | -1.878 | 0.001734 | 0.030928 |
| Unigene0109255 | MACPF domain-containing protein NSL1 [Sorghum bicolor] | NSL1 | 20.59 | 2.366667 | -3.12102 | 4.36E-24 | 7.57E-22 |
| Unigene0050233 | mitogen-activated protein kinase 4 [Saccharum hybrid cultivar ROC22] | MPK5 | 425.2833 | 55.52333 | -2.93726 | 2.58E-53 | 1.71E-50 |
| Unigene0040118 | mitogen-activated protein kinase 4 [Sorghum bicolor] | MPK4 | 73.35 | 14.34 | -2.35475 | 3.97E-49 | 2.22E-46 |
| Unigene0043677 | Orphans transcription factor [Saccharum spontaneum] | BBX22 | 18.28667 | 6.086667 | -1.58707 | 1.15E-24 | 2.07E-22 |
| Unigene0113415 | patatin-like protein 2 [Sorghum bicolor] | PLP2 | 5.703333 | 0.333333 | -4.09677 | 3.48E-25 | 6.50E-23 |
| Unigene0066387 | photosystem II subunit O-2 [Arabidopsis thaliana] | PSBO2 | 3.006667 | 1.12 | -1.42467 | 0.002303 | 0.038744 |
| Unigene0009358 | polygalacturonase inhibitor [Sorghum bicolor] | PGIP1 | 8.383333 | 24.61333 | 1.553844 | 4.45E-06 | 0.000165 |
| Unigene0009359 | polygalacturonase inhibitor [Sorghum bicolor] | PGIP1 | 8.953333 | 20.99667 | 1.229664 | 2.06E-05 | 0.000661 |
| Unigene0053255 | probable 1-acyl-sn-glycerol-3-phosphate acyltransferase 5 [Sorghum bicolor] | LPAT5 | 10.84667 | 3.443333 | -1.65537 | 3.60E-17 | 4.33E-15 |
| Unigene0000501 | probable E3 ubiquitin-protein ligase XBOS34 [Sorghum bicolor] | XBOS34 | 2.763333 | 1.26 | -1.13299 | 2.49E-06 | 9.71E-05 |
| Unigene0104995 | probable LRR receptor-like serine/threonine-protein kinase At1g74360 [Sorghum bicolor] | At1g74360 | 48.67333 | 16.97667 | -1.51958 | 8.49E-09 | 4.84E-07 |
| Unigene0080658 | protein DOWNY MILDEW RESISTANCE 6 [Sorghum bicolor] | F3H-2 | 4.91 | 2.146667 | -1.19362 | 7.13E-05 | 0.002003 |
| Unigene0022129 | protein LURP-one-related 15-like [Panicum miliaceum] | At5g01750 | 2.516667 | 0.753333 | -1.74015 | 0.002672 | 0.043764 |
| Unigene0028710 | protein MLO [Sorghum bicolor] | MLO-H1 | 1.196667 | 0.163333 | -2.87313 | 5.77E-05 | 0.001658 |
| Unigene0028123 | protein phosphatase 2C 35 [Sorghum bicolor] | XB15 | 29.49 | 3.633333 | -3.02086 | 7.92E-32 | 2.09E-29 |
| Unigene0055235 | PVR3-like protein [Zea mays] | DIR1 | 10.89667 | 25.32667 | 1.21677 | 0.001341 | 0.024926 |
| Unigene0019857 | pyruvate dehydrogenase phosphatase [Salvia splendens] | Os05g0111800 | 6.653333 | 2.463333 | -1.43347 | 3.01E-05 | 0.000924 |
| Unigene0088999 | stem-specific protein TSJT1 [Oryza sativa Japonica Group] | TSJT1 | 0.79 | 0.001 | -9.62571 | 7.16E-05 | 0.002006 |
| Unigene0025688 | transcription factor MYB44 [Sorghum bicolor] | MYB73 | 5.81 | 1.513333 | -1.94081 | 7.46E-06 | 0.000265 |
| Unigene0083071 | aminotransferase ALD1 homolog [Sorghum bicolor] | ALD1 | 4.81 | 17.38 | 1.853319 | 2.01E-05 | 0.000648 |
| Unigene0080205 | cytochrome P450 [Saccharum hybrid cultivar R570] | CYP74A1 | 295.56 | 57.96667 | -2.35016 | 3.36E-15 | 3.48E-13 |
| Unigene0024887 | NAC domain-containing protein 92 [Sorghum bicolor] | NAC079 | 2.143333 | 9.98 | 2.219184 | 3.63E-05 | 0.001094 |
| Unigene0010489 | Grx_I1 - glutaredoxin subgroup III [Zea mays] | GRXS9 | 74.07 | 29.38667 | -1.33373 | 3.64E-05 | 0.001096 |
| Unigene0074573 | monothiol glutaredoxin-S5-like [Panicum hallii] | GRXS5 | 53.60667 | 3.683333 | -3.86333 | 2.16E-08 | 1.15E-06 |
| Unigene0075079 | probable protein phosphatase 2C 30 [Sorghum bicolor] | PP2C30 | 110.3667 | 28.44333 | -1.95614 | 2.25E-15 | 2.34E-13 |
| Unigene0075078 | probable protein phosphatase 2C 49 [Sorghum bicolor] | Os05g0457200 | 6.546667 | 2.853333 | -1.19811 | 8.17E-05 | 0.002247 |
| Unigene0052049 | serine/threonine-protein kinase SAPK2 [Panicum miliaceum] | SAPK2 | 29.20333 | 6.36 | -2.19903 | 2.56E-31 | 6.38E-29 |
| Unigene0039644 | transcription factor HY5 [Sorghum bicolor] | HY5 | 2.536667 | 0.316667 | -3.0019 | 0.000146 | 0.003748 |
| GO:0071554 Cell Wall Organization or Biogenesis | | | | |  |  |  |
| Unigenes | Description | Symbol | CK_mean_rpkm | T_mean_rpkm | log2(fc) | PValue | FDR |
| Unigene0088856 | 3-ketoacyl-CoA synthase 11 [Sorghum bicolor] | KCS11 | 149.87 | 26.57667 | -2.49548 | 1.67E-33 | 4.74E-31 |
| Unigene0088738 | 3-ketoacyl-CoA synthase 11 [Sorghum bicolor] | KCS11 | 11.00667 | 4.753333 | -1.21137 | 3.06E-05 | 0.000936 |
| Unigene0056348 | 40S ribosomal protein S10-A-like [Quercus suber] | rps1002 | 0.793333 | 0.023333 | -5.08746 | 0.002251 | 0.038036 |
| Unigene0110700 | 40S ribosomal protein S26-like [Oryza sativa Japonica Group] | RPS26 | 3.196667 | 0.001 | -11.6424 | 4.69E-08 | 2.39E-06 |
| Unigene0020794 | 60S acidic ribosomal protein P0 [Oryza sativa Japonica Group] | Os08g0130500 | 1.613333 | 0.001 | -10.6558 | 2.79E-08 | 1.46E-06 |
| Unigene0001466 | 60S acidic ribosomal protein P1 [Oryza sativa Japonica Group] | RPP1A | 3.386667 | 0.036667 | -6.52925 | 7.40E-07 | 3.16E-05 |
| Unigene0075223 | 60S ribosomal protein L3 [Oryza sativa Japonica Group] | RPL3 | 0.823333 | 0.001 | -9.68533 | 0.000636 | 0.013375 |
| Unigene0099693 | 60S ribosomal protein L3-like [Rhodamnia argentea] | RPL3 | 6.39 | 2.546667 | -1.32721 | 0.001196 | 0.022778 |
| Unigene0057000 | 6-phosphogluconate dehydrogenase, decarboxylating 1-like [Quercus suber] | GND1 | 0.956667 | 0.046667 | -4.35755 | 3.04E-05 | 0.000933 |
| Unigene0014455 | callose synthase 12 [Sorghum bicolor] | CALS12 | 25.57667 | 10.4 | -1.29824 | 9.35E-13 | 8.07E-11 |
| Unigene0020634 | Cellulose synthase A catalytic subunit 5 [UDP-forming] [Zea mays] | CESA6 | 27.61667 | 10.96 | -1.33329 | 8.19E-08 | 4.05E-06 |
| Unigene0003063 | cellulose synthase-like protein D2 [Sorghum bicolor] | CSLD2 | 120.9567 | 13.98333 | -3.11271 | 3.59E-67 | 4.14E-64 |
| Unigene0071649 | chitinase [Saccharum hybrid cultivar Yacheng05-179] | CHIA | 324.6833 | 1600.273 | 2.301213 | 1.37E-12 | 1.16E-10 |
| Unigene0071651 | chitinase 6 [Sorghum bicolor] | Cht6 | 18.89 | 5.77 | -1.71098 | 4.86E-10 | 3.22E-08 |
| Unigene0024550 | chloride channel protein CLC-a [Sorghum bicolor] | CLC-B | 23.96333 | 10.14667 | -1.23982 | 6.28E-05 | 0.001791 |
| Unigene0045004 | COBRA-like protein 3 [Sorghum bicolor] | BC1L4 | 2.823333 | 0.336667 | -3.06801 | 4.23E-07 | 1.88E-05 |
| Unigene0033468 | COBRA-like protein 5 [Sorghum bicolor] | BC1 | 8.666667 | 18.10667 | 1.062972 | 0.000209 | 0.005114 |
| Unigene0002182 | coleoptile phototropism protein 1 [Sorghum bicolor] | CPT1 | 3.623333 | 7.816667 | 1.109236 | 0.00052 | 0.011248 |
| Unigene0002319 | endochitinase A [Sorghum bicolor] | CHIA | 19.73 | 87.60667 | 2.15065 | 1.85E-08 | 9.94E-07 |
| Unigene0028650 | expansin 37 [Saccharum hybrid cultivar] | EXPA9 | 0.72 | 2.7 | 1.906891 | 0.000557 | 0.011915 |
| Unigene0044633 | expansin 40 [Saccharum hybrid cultivar] | EXLA1 | 23.66333 | 55.91333 | 1.240539 | 3.84E-07 | 1.72E-05 |
| Unigene0023361 | expansin 77 [Saccharum hybrid cultivar] | EXLA3 | 12.55 | 41.64667 | 1.730514 | 1.48E-05 | 0.000493 |
| Unigene0023750 | expansin-like A2 [Sorghum bicolor] | EXLA2 | 8.15 | 1.876667 | -2.11863 | 2.91E-09 | 1.76E-07 |
| Unigene0069638 | fasciclin-like arabinogalactan protein 11 [Sorghum bicolor] | FLA9 | 92.50667 | 32.21667 | -1.52175 | 1.35E-08 | 7.44E-07 |
| Unigene0004639 | F-box protein At3g49450 [Sorghum bicolor] | JAMYB | 43.77667 | 11.10333 | -1.97917 | 6.37E-14 | 6.03E-12 |
| Unigene0075788 | galactosylgalactosylxylosylprotein 3-beta-glucuronosyltransferase 1 [Zea mays] | IRX9L | 0.713333 | 0.13 | -2.45606 | 0.00213 | 0.036374 |
| Unigene0064873 | Glycerophosphodiester phosphodiesterase GDPDL3 [Zea mays] | GDPDL1 | 31.24667 | 7.443333 | -2.06968 | 1.26E-28 | 2.81E-26 |
| Unigene0091566 | glycosyltransferase [Zea mays] | Os10g0205300 | 5.673333 | 1.39 | -2.02911 | 3.91E-07 | 1.74E-05 |
| Unigene0044885 | GTP-binding protein rhoA-like [Quercus suber] | rhoA | 0.66 | 0.016667 | -5.30743 | 0.001264 | 0.023856 |
| Unigene0061628 | LysM domain-containing protein [Cephalotus follicularis] | -- | 1.373333 | 6.11 | 2.153491 | 0.00053 | 0.011433 |
| Unigene0042910 | lysM receptor-like kinase [Zea mays] | CERK1 | 48.48 | 12.15333 | -1.99604 | 4.55E-28 | 9.82E-26 |
| Unigene0043984 | mitogen-activated protein kinase 7 isoform X1 [Sorghum bicolor] | MPK7 | 28.92 | 8.523333 | -1.76258 | 1.60E-18 | 2.10E-16 |
| Unigene0088254 | MYB transcription factor 103 [Miscanthus sinensis var. sinensis] [Miscanthus sinensis] | MYB86 | 1.593333 | 3.873333 | 1.281528 | 0.002408 | 0.040197 |
| Unigene0072616 | Myb-related protein Hv33 [Zea mays] | MYB2 | 1.963333 | 6.746667 | 1.78087 | 6.75E-05 | 0.001906 |
| Unigene0049148 | pectinesterase [Zea mays] | PME41 | 12.39333 | 29.59667 | 1.25587 | 1.49E-05 | 0.000496 |
| Unigene0049149 | pectinesterase [Zea mays] | PME20 | 6.233333 | 14.65 | 1.232825 | 0.003147 | 0.049799 |
| Unigene0032220 | plasma membrane ATPase-like [Quercus suber] | pma-1 | 1.326667 | 0.096667 | -3.77864 | 0.000195 | 0.004815 |
| Unigene0032219 | plasma membrane ATPase-like [Quercus suber] | pma-1 | 0.646667 | 0.001 | -9.33688 | 0.00048 | 0.010527 |
| Unigene0044112 | PREDICTED: 40S ribosomal protein S14 [Oryza brachyantha] | RPS14C | 1.28 | 0.001 | -10.3219 | 7.36E-05 | 0.002049 |
| Unigene0030838 | PREDICTED: transmembrane protein 45B-like [Oryza brachyantha] | -- | 1.966667 | 7.243333 | 1.880901 | 0.002208 | 0.037475 |
| Unigene0051815 | probable fucosyltransferase 8 [Sorghum bicolor] | FUT1 | 15.10333 | 2.763333 | -2.45039 | 7.33E-41 | 2.96E-38 |
| Unigene0089079 | probable glucuronosyltransferase Os03g0287800 [Sorghum bicolor] | Os03g0287800 | 3.013333 | 1.033333 | -1.54405 | 2.04E-07 | 9.46E-06 |
| Unigene0012718 | probable mixed-linked glucan synthase 6 [Sorghum bicolor] | CSLF6 | 86.46 | 29.35667 | -1.55834 | 1.56E-15 | 1.65E-13 |
| Unigene0014769 | probable pectinesterase/pectinesterase inhibitor 51 [Sorghum bicolor] | PME51 | 7.576667 | 1.86 | -2.02626 | 4.14E-13 | 3.69E-11 |
| Unigene0058295 | probable protein phosphatase 2C 59 isoform X2 [Oryza sativa Japonica Group] | Os06g0698300 | 3.65 | 7.596667 | 1.05747 | 0.000562 | 0.011998 |
| Unigene0099142 | probable UTP--glucose-1-phosphate uridylyltransferase [Quercus suber] | fyu1 | 0.793333 | 0.001 | -9.63178 | 0.001926 | 0.033508 |
| Unigene0039476 | probable xyloglucan endotransglucosylase/hydrolase protein 25 [Setaria italica] | XTH25 | 57.22 | 18.71667 | -1.6122 | 1.05E-06 | 4.39E-05 |
| Unigene0042033 | proline-rich receptor-like protein kinase PERK1 isoform X1 [Brachypodium distachyon] | PERK1 | 28.38 | 6.55 | -2.11531 | 1.97E-23 | 3.33E-21 |
| Unigene0011332 | proline-rich receptor-like protein kinase PERK2 [Zea mays] | PERK1 | 4.123333 | 1.536667 | -1.42401 | 2.48E-05 | 0.000777 |
| Unigene0108182 | proline-rich receptor-like protein kinase PERK5 isoform X2 [Panicum hallii] | PBL15 | 1.04 | 0.023333 | -5.47805 | 2.29E-05 | 0.000723 |
| Unigene0088797 | Protein NRT1/ PTR FAMILY 6.3 [Zea mays] | NPF6.3 | 1.01 | 0.18 | -2.48829 | 0.002266 | 0.038265 |
| Unigene0039867 | Protein WALLS ARE THIN 1 [Zea mays] | WAT1 | 39.44667 | 17.67 | -1.1586 | 0.000481 | 0.01055 |
| Unigene0018503 | protein YLS3 [Setaria italica] | YLS3 | 1.126667 | 5.31 | 2.236651 | 1.94E-06 | 7.75E-05 |
| Unigene0031082 | thaumatin-like protein 1b [Sorghum bicolor] | TLP1 | 1.056667 | 6.186667 | 2.549642 | 5.55E-09 | 3.23E-07 |
| Unigene0003733 | transcription factor JAMYB [Sorghum bicolor] | JAMYB | 50.08667 | 107.4033 | 1.10054 | 2.09E-05 | 0.000669 |
| Unigene0051165 | transcription factor MYB2 [Sorghum bicolor] | MYB2 | 12.87333 | 2.25 | -2.51639 | 3.41E-09 | 2.04E-07 |
| Unigene0047103 | transcription factor MYB82-like [Sorghum bicolor] | PP2 | 1.513333 | 6.386667 | 2.077333 | 1.32E-05 | 0.000444 |
| Unigene0082098 | transcription repressor OFP3 [Sorghum bicolor] | OFP2 | 10.19 | 0.583333 | -4.12669 | 1.09E-31 | 2.83E-29 |
| Unigene0003002 | ubiquitin-40S ribosomal protein S27a-like [Citrus sinensis] | -- | 77.17667 | 26.41667 | -1.54672 | 2.37E-08 | 1.26E-06 |
| Unigene0009565 | UDP-glucose 4-epimerase 1 [Sorghum bicolor] | UGE-1 | 41.49333 | 16.34 | -1.34447 | 2.87E-09 | 1.74E-07 |
| Unigene0085405 | UDP-glucuronate:xylan alpha-glucuronosyltransferase 1 isoform X1 [Sorghum bicolor] | GUX1 | 1.173333 | 3.716667 | 1.663396 | 0.000256 | 0.006124 |
| Unigene0016331 | xyloglucan endotransglucosylase/hydrolase protein 22 [Sorghum bicolor] | XTH22 | 301.9367 | 9.026667 | -5.06391 | ######## | ######## |
| Unigene0008193 | Xyloglucan endotransglucosylase/hydrolase protein 24 [Saccharum officinarum] | XTH24 | 38.14667 | 0.793333 | -5.58749 | 7.37E-78 | 1.22E-74 |
| Unigene0003219 | xyloglucan endotransglucosylase/hydrolase protein 31 [Sorghum bicolor] | XTH32 | 6.213333 | 15.55 | 1.323475 | 2.31E-06 | 9.10E-05 |
| Unigene0011327 | zink finger-like fusion protein [Guillardia theta CCMP2712] | RPS27AA | 2.833333 | 0.063333 | -5.48339 | 1.13E-06 | 4.69E-05 |
| Unigene0039644 | transcription factor HY5 [Sorghum bicolor] | HY5 | 2.536667 | 0.316667 | -3.0019 | 0.000146 | 0.003748 |
| GO:0009845 Seed Germination | | |  |  |  |  |  |
| Unigenes | Description | Symbol | CK_mean_rpkm | T_mean_rpkm | log2(fc) | PValue | FDR |
| Unigene0093122 | EID1-like F-box protein 3 [Sorghum bicolor] | EDL3 | 54.63667 | 4.19 | -3.70485 | 1.14E-23 | 1.94E-21 |
| Unigene0067567 | EID1-like F-box protein 3 [Sorghum bicolor] | EDL3 | 5.713333 | 0.29 | -4.30021 | 7.92E-14 | 7.43E-12 |
| Unigene0032457 | heat shock cognate protein 70-1 [Arabidopsis thaliana] | HSP70 | 2.996667 | 1.163333 | -1.36509 | 0.002082 | 0.035717 |
| Unigene0004408 | heat shock protein 90 [Oryza sativa Japonica Group] | HSP81-2 | 1.126667 | 0.053333 | -4.40088 | 7.00E-05 | 0.001967 |
| Unigene0003292 | heat shock protein 90-like, partial [Quercus suber] | hsp90 | 2.656667 | 0.026667 | -6.63844 | 1.56E-06 | 6.33E-05 |
| Unigene0086406 | PREDICTED: heat shock 70 kDa protein 18-like [Raphanus sativus] | HSC-2 | 1.69 | 0.001 | -10.7228 | 0.000967 | 0.01907 |
| Unigene0111066 | two-component response regulator ORR9 [Sorghum bicolor] | RR9 | 10.58333 | 23.56667 | 1.154954 | 1.59E-06 | 6.46E-05 |
| Unigene0030479 | U-box domain-containing protein 19 [Sorghum bicolor] | PUB19 | 49.87333 | 1.316667 | -5.24331 | ######## | ######## |
| Unigene0075079 | probable protein phosphatase 2C 30 [Sorghum bicolor] | PP2C30 | 110.3667 | 28.44333 | -1.95614 | 2.25E-15 | 2.34E-13 |
| Unigene0075078 | probable protein phosphatase 2C 49 [Sorghum bicolor] | Os05g0457200 | 6.546667 | 2.853333 | -1.19811 | 8.17E-05 | 0.002247 |
| Unigene0052049 | serine/threonine-protein kinase SAPK2 [Panicum miliaceum] | SAPK2 | 29.20333 | 6.36 | -2.19903 | 2.56E-31 | 6.38E-29 |
| GO:0010243 Response to Organonitrogen Compound | | | | | |  |  |
| Unigenes | Description | Symbol | CK_mean | T_mean | log2(fc) | P Value | FDR |
| Unigene0000501 | probable E3 ubiquitin-protein ligase XBOS34 [Sorghum bicolor] | XBOS34 | 2.763333 | 1.26 | -1.13299 | 2.49E-06 | 9.71E-05 |
| Unigene0000704 | ethylene-responsive transcription factor 1B [Sorghum bicolor] | ERF1B | 3.99 | 10.90667 | 1.45075 | 4.66E-05 | 0.001373 |
| Unigene0001285 | transcription factor MYB4 [Sorghum bicolor] | MYB4 | 10.12667 | 2.63 | -1.94502 | 1.36E-12 | 1.15E-10 |
| Unigene0001764 | receptor-like protein kinase 5 [Sorghum bicolor] | RLK5 | 41.59667 | 90.98667 | 1.129187 | 1.91E-06 | 7.63E-05 |
| Unigene0002319 | endochitinase A [Sorghum bicolor] | CHIA | 19.73 | 87.60667 | 2.15065 | 1.85E-08 | 9.94E-07 |
| Unigene0002370 | ethylene-responsive transcription factor 11 [Sorghum bicolor] | ERF4 | 152.68 | 43.56 | -1.80944 | 4.63E-10 | 3.09E-08 |
| Unigene0002960 | Monoglyceride lipase [Dichanthelium oligosanthes] | MGLL | 9.456667 | 3.72 | -1.34603 | 1.95E-10 | 1.36E-08 |
| Unigene0004164 | receptor-like protein kinase 5 [Sorghum bicolor] | HAR1 | 23.15 | 46.74333 | 1.013748 | 8.97E-06 | 0.000313 |
| Unigene0008193 | Xyloglucan endotransglucosylase/hydrolase protein 24 [Saccharum officinarum] | XTH24 | 38.14667 | 0.793333 | -5.58749 | 7.37E-78 | 1.22E-74 |
| Unigene0010528 | ethylene-responsive transcription factor 3-like [Sorghum bicolor] | ERF3 | 281.3633 | 86.69667 | -1.69839 | 6.79E-08 | 3.39E-06 |
| Unigene0010928 | carbonic anhydrase 1 [Arabidopsis thaliana] | BCA1 | 4.016667 | 0.79 | -2.34607 | 0.000137 | 0.003562 |
| Unigene0011983 | ethylene-responsive transcription factor 8 [Sorghum bicolor] | ERF4 | 386.7167 | 121.15 | -1.67448 | 4.77E-07 | 2.09E-05 |
| Unigene0011984 | AP2 domain-containing protein [Sorghum bicolor] | ERF4 | 227.5333 | 104.1467 | -1.12746 | 5.72E-08 | 2.88E-06 |
| Unigene0012135 | WRKY39v2 - superfamily of TFs having WRKY and zinc finger domains [Zea mays] | WRKY27 | 11.38667 | 0.636667 | -4.16066 | 4.30E-35 | 1.31E-32 |
| Unigene0012136 | WRKY transcription factor WRKY51 isoform X2 [Zea mays] | WRKY21 | 32.38667 | 13.29333 | -1.2847 | 8.26E-09 | 4.72E-07 |
| Unigene0014519 | F-box protein [Zea mays] | At1g78280 | 17.67333 | 6.08 | -1.53943 | 1.66E-16 | 1.90E-14 |
| Unigene0015758 | probable xyloglucan 6-xylosyltransferase 1 [Panicum hallii] | XXT1 | 46.93 | 7.59 | -2.62834 | 3.55E-33 | 9.90E-31 |
| Unigene0016331 | xyloglucan endotransglucosylase/hydrolase protein 22 [Sorghum bicolor] | XTH22 | 301.9367 | 9.026667 | -5.06391 | ######## | ######## |
| Unigene0022129 | protein LURP-one-related 15-like [Panicum miliaceum] | At5g01750 | 2.516667 | 0.753333 | -1.74015 | 0.002672 | 0.043764 |
| Unigene0022448 | 9-cis-epoxycarotenoid dioxygenase 1, chloroplastic [Dichanthelium oligosanthes] | -- | 14.47 | 3.693333 | -1.97007 | 2.43E-06 | 9.51E-05 |
| Unigene0022497 | transcription factor MYB4 [Sorghum bicolor] | MYB30 | 3.396667 | 1.396667 | -1.28213 | 0.00184 | 0.032306 |
| Unigene0023853 | HS1-like protein [Saccharum hybrid cultivar R570] | HSPRO2 | 2092.547 | 231.2967 | -3.17744 | 6.60E-47 | 3.41E-44 |
| Unigene0026276 | ACC synthase 2 [Saccharum hybrid cultivar SP80-3280] | ACS7 | 45.59333 | 1.12 | -5.34725 | 4.39E-71 | 5.58E-68 |
| Unigene0028710 | protein MLO [Sorghum bicolor] | MLO-H1 | 1.196667 | 0.163333 | -2.87313 | 5.77E-05 | 0.001658 |
| Unigene0030481 | calcium-transporting ATPase 10, plasma membrane-type [Sorghum bicolor] | ACA2 | 94.39333 | 5.78 | -4.02954 | ######## | ######## |
| Unigene0032850 | abscisic acid 8'-hydroxylase 1 [Sorghum bicolor] | CYP707A5 | 28.58 | 2.323333 | -3.62074 | 2.88E-27 | 6.00E-25 |
| Unigene0032851 | abscisic acid 8'-hydroxylase 1 [Sorghum bicolor] | CYP707A5 | 73.53667 | 4.62 | -3.9925 | 2.92E-37 | 9.98E-35 |
| Unigene0036505 | heat stress transcription factor A-4b [Sorghum bicolor] | HSFA4B | 45.42667 | 11.51333 | -1.98023 | 4.60E-34 | 1.34E-31 |
| Unigene0037820 | NAC domain-containing protein 90 [Zea mays] | NAC061 | 3.073333 | 0.34 | -3.1762 | 9.68E-11 | 7.01E-09 |
| Unigene0039476 | probable xyloglucan endotransglucosylase/hydrolase protein 25 [Setaria italica] | XTH25 | 57.22 | 18.71667 | -1.6122 | 1.05E-06 | 4.39E-05 |
| Unigene0042910 | lysM receptor-like kinase [Zea mays] | CERK1 | 48.48 | 12.15333 | -1.99604 | 4.55E-28 | 9.82E-26 |
| Unigene0045328 | NAC domain-containing [Saccharum hybrid cultivar SP80-3280] | NAC090 | 9.306667 | 1.54 | -2.59533 | 1.89E-14 | 1.85E-12 |
| Unigene0047118 | U-box domain-containing protein 25 [Sorghum bicolor] | PUB25 | 1.35 | 5.286667 | 1.969399 | 1.49E-07 | 7.09E-06 |
| Unigene0049376 | probable protein phosphatase 2C 2 [Sorghum bicolor] | Os01g0295700 | 230.9967 | 29.59667 | -2.96437 | 1.64E-62 | 1.63E-59 |
| Unigene0049575 | probable E3 ubiquitin-protein ligase XERICO [Sorghum bicolor] | XERICO | 57.59667 | 19.36667 | -1.57241 | 9.55E-09 | 5.39E-07 |
| Unigene0050233 | mitogen-activated protein kinase 4 [Saccharum hybrid cultivar ROC22] | MPK5 | 425.2833 | 55.52333 | -2.93726 | 2.58E-53 | 1.71E-50 |
| Unigene0050621 | probable protein phosphatase 2C 47 [Sorghum bicolor] | PP2C27 | 12.00667 | 5.21 | -1.20448 | 5.08E-09 | 2.96E-07 |
| Unigene0054023 | AP2-EREBP transcription factor, partial [Zea mays] | PTI5 | 3.706667 | 16.62333 | 2.165015 | 0.002896 | 0.046701 |
| Unigene0054038 | WRKY transcription factor 6 isoform X1 [Sorghum bicolor] | WRKY6 | 46.85667 | 12.56 | -1.89942 | 5.76E-13 | 5.03E-11 |
| Unigene0058460 | Glycosyltransferase family 61 protein [Hordeum vulgare] | MUCI21 | 129.2967 | 51.70333 | -1.32236 | 3.68E-08 | 1.90E-06 |
| Unigene0064385 | H0425E08.4 [Oryza sativa] | METTL18 | 40.25 | 17.80333 | -1.17684 | 1.64E-12 | 1.39E-10 |
| Unigene0064447 | probable choline kinase 2 isoform X1 [Panicum hallii] | At1g74320 | 8.15 | 3.576667 | -1.18818 | 1.09E-07 | 5.27E-06 |
| Unigene0064619 | ethylene-responsive transcription factor ERF109 [Sorghum bicolor] | ERF109 | 1117.94 | 35.65333 | -4.97066 | 1.68E-62 | 1.64E-59 |
| Unigene0066387 | photosystem II subunit O-2 [Arabidopsis thaliana] | PSBO2 | 3.006667 | 1.12 | -1.42467 | 0.002303 | 0.038744 |
| Unigene0070005 | mitochondrial uncoupling protein 5 [Saccharum officinarum] | PUMP5 | 557.06 | 65.13667 | -3.09629 | 3.05E-20 | 4.36E-18 |
| Unigene0070874 | aspartyl protease family protein 2 [Sorghum bicolor] | APF2 | 152.8267 | 26.70333 | -2.5168 | 2.58E-25 | 4.87E-23 |
| Unigene0071649 | chitinase [Saccharum hybrid cultivar Yacheng05-179] | CHIA | 324.6833 | 1600.273 | 2.301213 | 1.37E-12 | 1.16E-10 |
| Unigene0079426 | exocyst complex component EXO70B1 [Sorghum bicolor] | EXO70B1 | 136.5533 | 50.28 | -1.44141 | 1.24E-21 | 1.89E-19 |
| Unigene0080151 | ethylene-responsive transcription factor ERF109 [Sorghum bicolor] | ERF110 | 9.33 | 0.833333 | -3.48491 | 4.21E-08 | 2.15E-06 |
| Unigene0080205 | cytochrome P450 [Saccharum hybrid cultivar R570] | CYP74A1 | 295.56 | 57.96667 | -2.35016 | 3.36E-15 | 3.48E-13 |
| Unigene0080658 | protein DOWNY MILDEW RESISTANCE 6 [Sorghum bicolor] | F3H-2 | 4.91 | 2.146667 | -1.19362 | 7.13E-05 | 0.002003 |
| Unigene0081239 | ethylene-responsive transcription factor ERF095-like [Panicum hallii] | ERF113 | 2.223333 | 0.153333 | -3.85798 | 5.39E-06 | 0.000197 |
| Unigene0081710 | probable WRKY transcription factor 70 [Setaria italica] | WRKY53 | 312.6433 | 47.49667 | -2.71862 | 4.78E-23 | 7.91E-21 |
| Unigene0085088 | formin-like protein 16 [Sorghum bicolor] | FH16 | 76.5 | 6.843333 | -3.48269 | 1.70E-61 | 1.59E-58 |
| Unigene0092072 | glutamyl-tRNA reductase, chloroplastic [Sorghum bicolor] | OsI_033071 | 38.14 | 17.87667 | -1.09323 | 1.57E-13 | 1.44E-11 |
| Unigene0093374 | probable carboxylesterase 6 [Sorghum bicolor] | CXE6 | 161.5633 | 50.82333 | -1.66854 | 1.84E-13 | 1.68E-11 |
| Unigene0094032 | 1-aminocyclopropane-1-carboxylate synthase 1 [Sorghum bicolor] | ACC1 | 1.326667 | 0.113333 | -3.54916 | 7.58E-08 | 3.76E-06 |
| Unigene0094996 | ethylene-responsive transcription factor ERF020 [Setaria italica] | ERF020 | 1.126667 | 0.001 | -10.1378 | 0.00108 | 0.020929 |
| Unigene0096131 | expressed protein [Oryza sativa Japonica Group] | -- | 38.66667 | 3.01 | -3.68326 | 3.51E-45 | 1.66E-42 |
| Unigene0096371 | vacuolar protein sorting-associated protein 2 homolog 1 [Sorghum bicolor] | VPS2.1 | 10.22667 | 4.85 | -1.07628 | 1.09E-05 | 0.000372 |
| Unigene0099873 | transcription factor MYB41 [Setaria italica] | MYB102 | 9.553333 | 1.323333 | -2.85183 | 1.13E-08 | 6.27E-07 |
| Unigene0101027 | U-box domain-containing protein 26 [Sorghum bicolor] | PUB25 | 20.28667 | 9.6 | -1.07943 | 1.77E-07 | 8.27E-06 |
| Unigene0104995 | probable LRR receptor-like serine/threonine-protein kinase At1g74360 [Sorghum bicolor] | At1g74360 | 48.67333 | 16.97667 | -1.51958 | 8.49E-09 | 4.84E-07 |
| Unigene0109255 | MACPF domain-containing protein NSL1 [Sorghum bicolor] | NSL1 | 20.59 | 2.366667 | -3.12102 | 4.36E-24 | 7.57E-22 |
| Unigene0113415 | patatin-like protein 2 [Sorghum bicolor] | PLP2 | 5.703333 | 0.333333 | -4.09677 | 3.48E-25 | 6.50E-23 |

**Supplementary figures:**

**
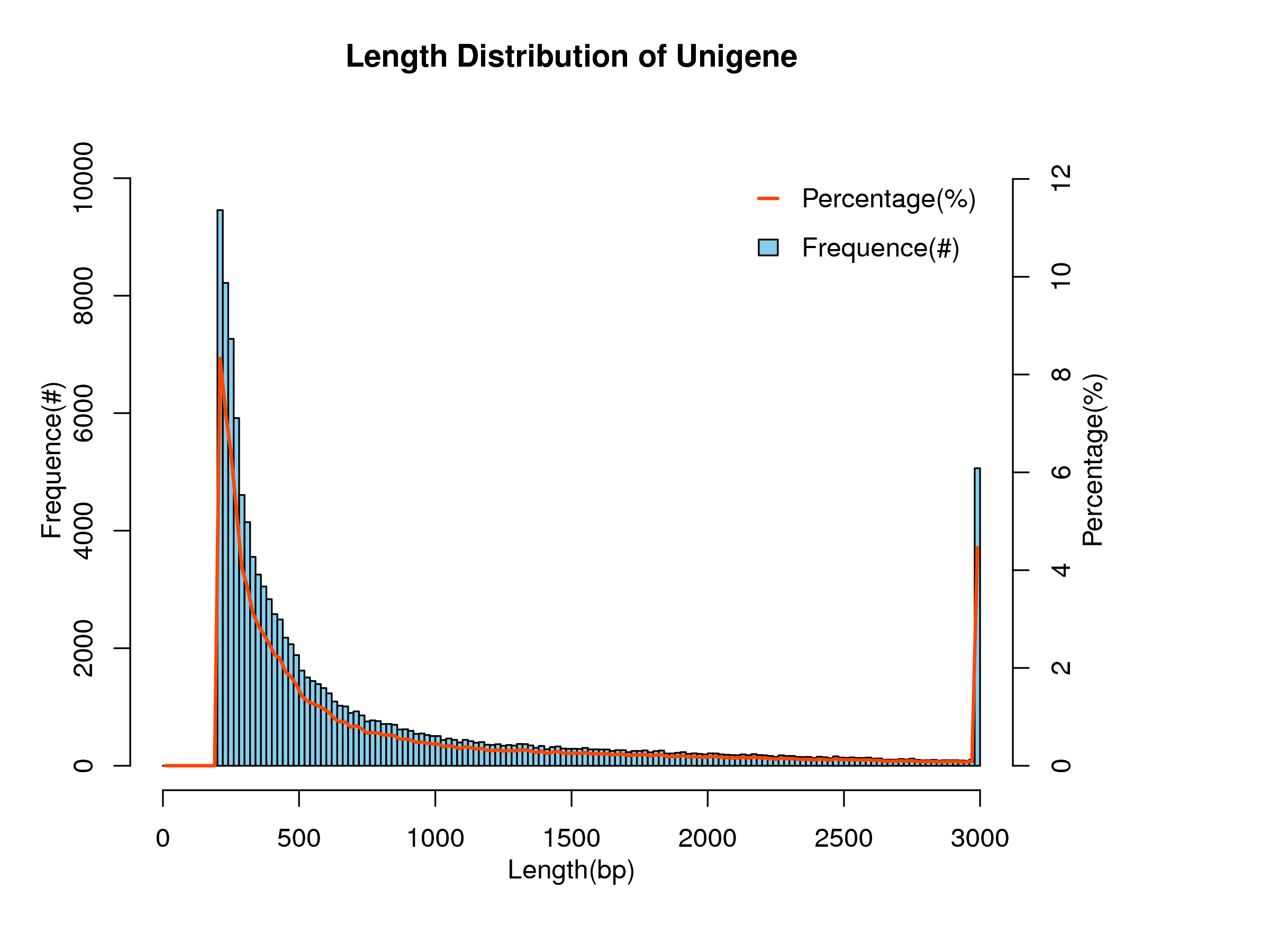
**

**Figure S1: Frequency and percent distribution of unigenes.**

**
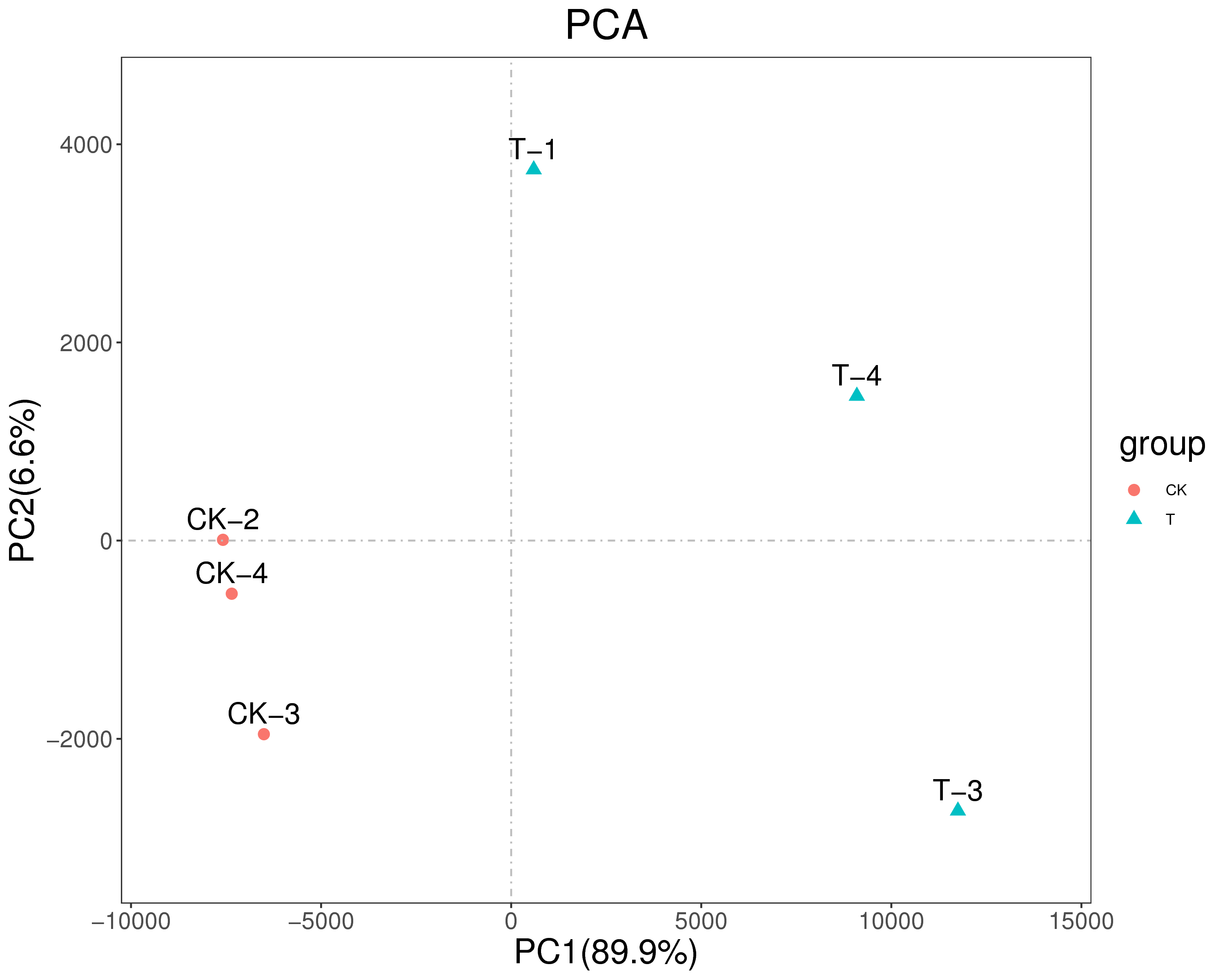
**

**Figure S2: Principal Component Analysis (PCA) denotes gene expression levels**

**
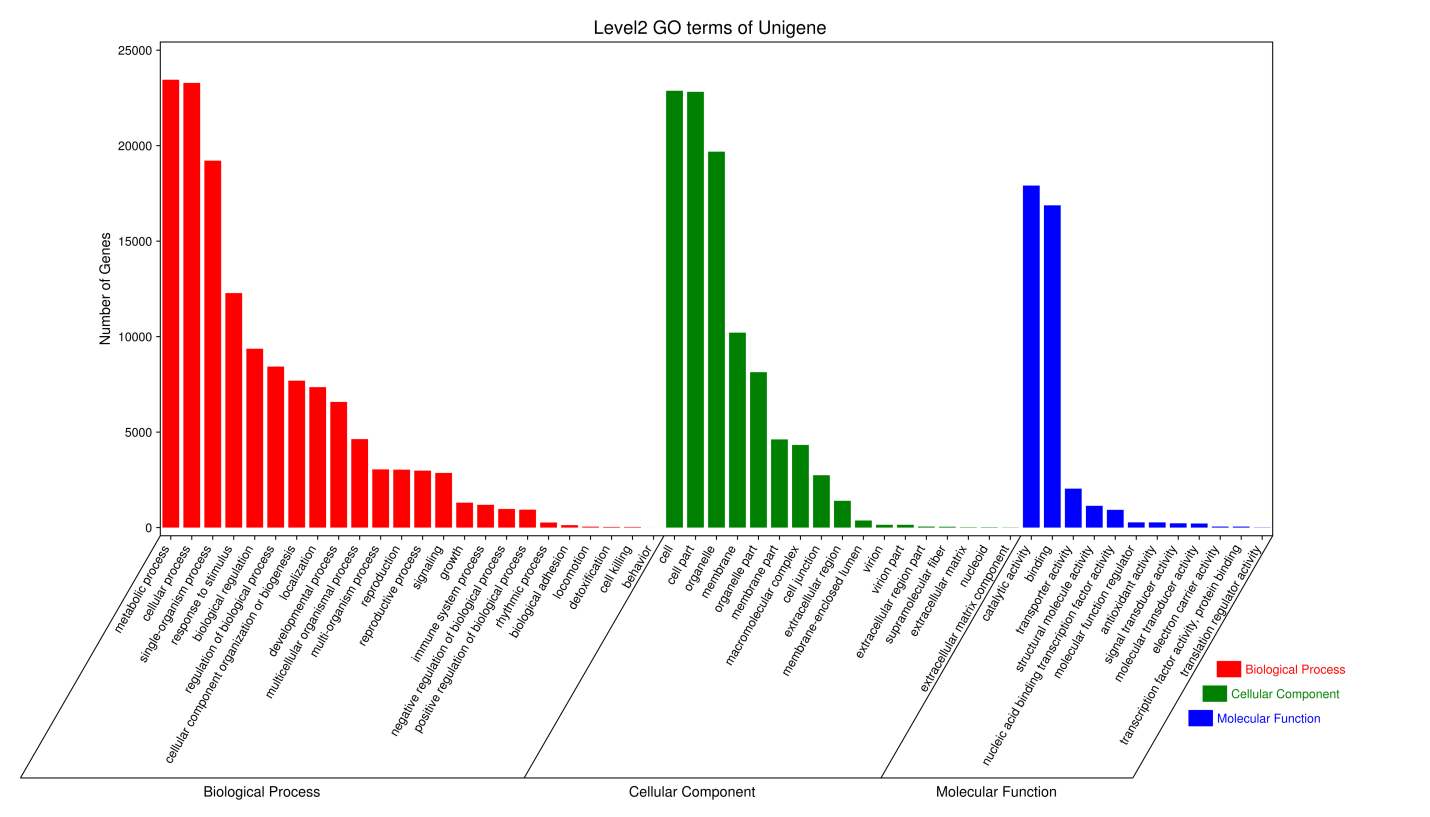
**

**Figure S3: Functional GO annotation of DGEs.**

**
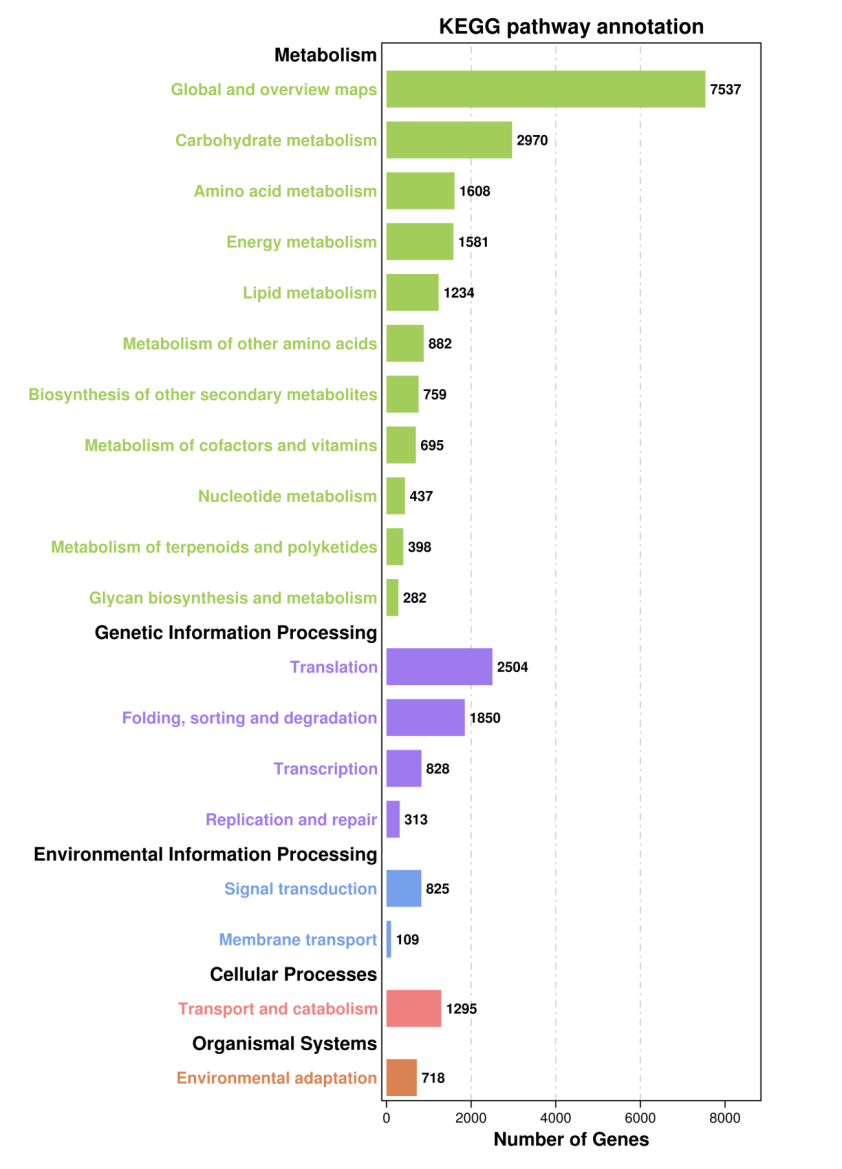
**

**Figure S4: Functional KEGG classification of the DGEs in the pathway analysis.**

**
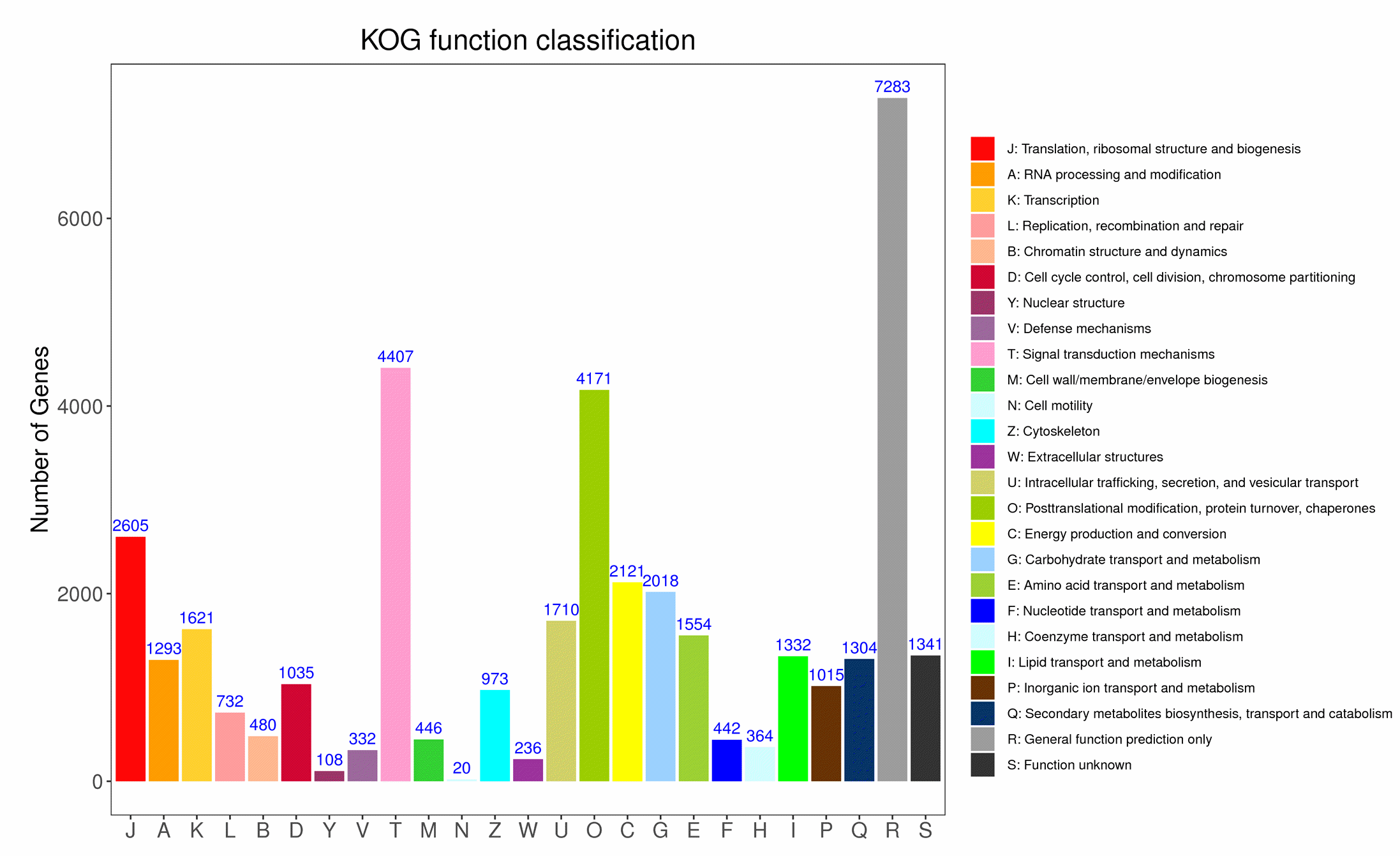
**

**Figure S5: KOG Function classification**

**
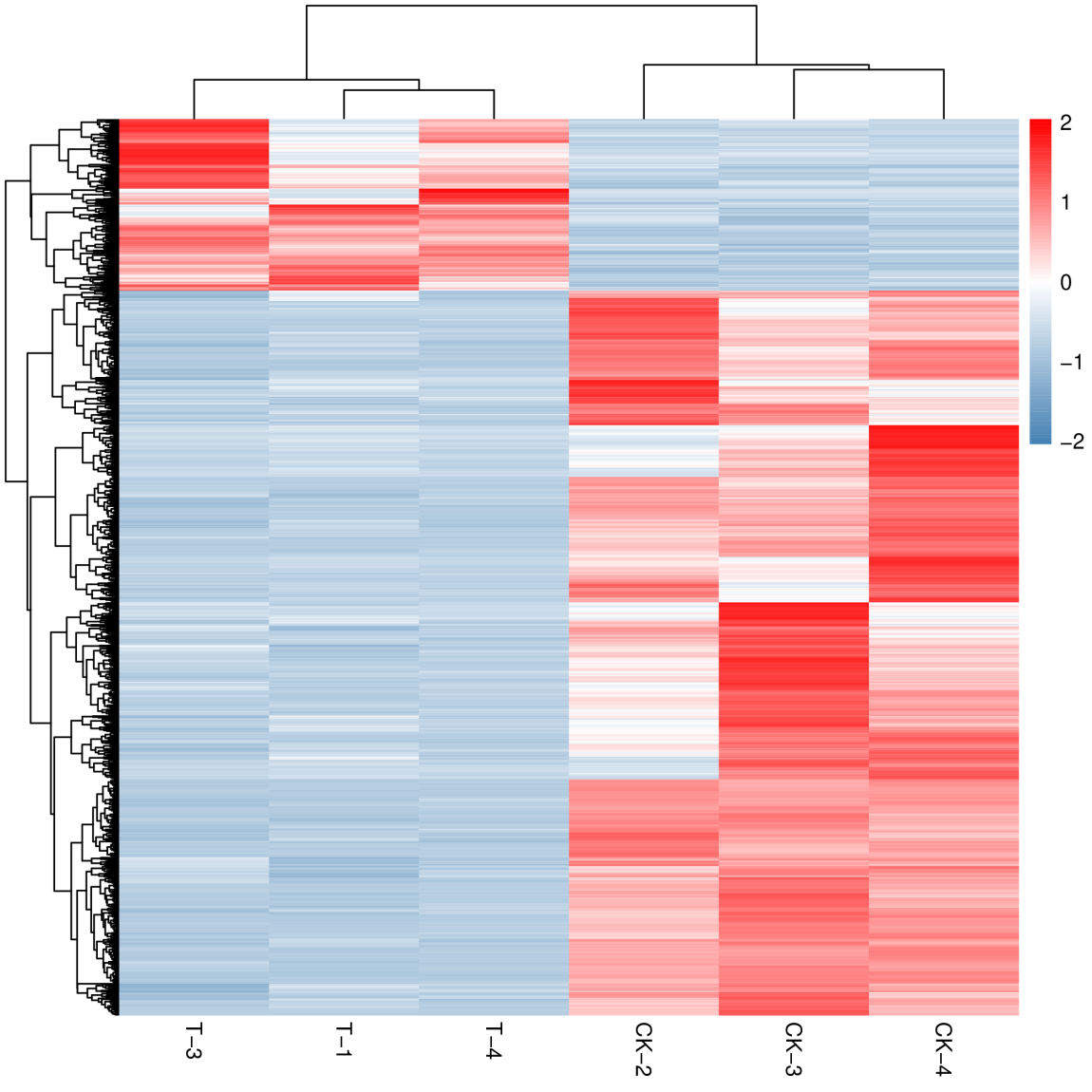
**

**Figure S6: Heatmap represents the** Hierarchical cluster analysis between the samples. Different columns in the figure represent different samples, and different rows represent different genes.

**
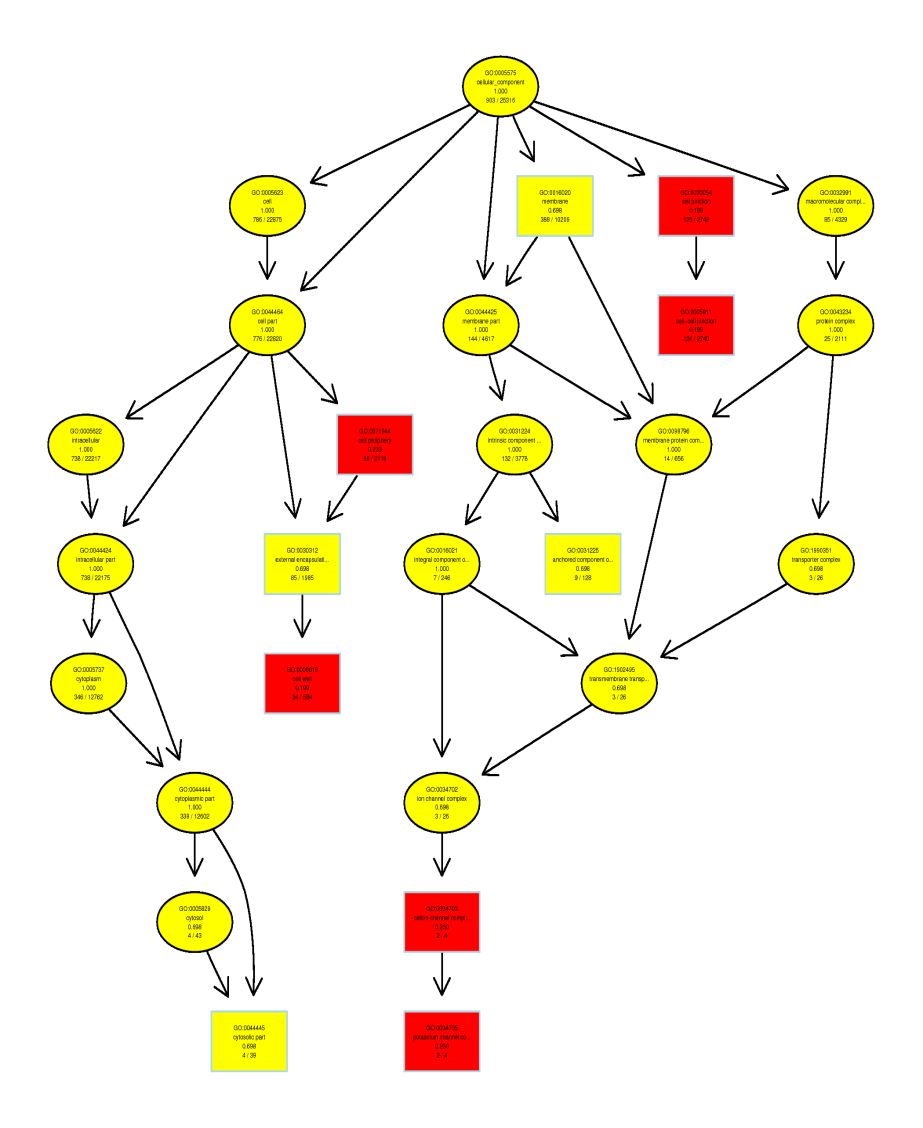
**

**Figure S7:** **Cellular component based enriched GO term in CK vs T. Red to yellow color exhibiting the high to low enrichment DEGs in each GO term**.

**
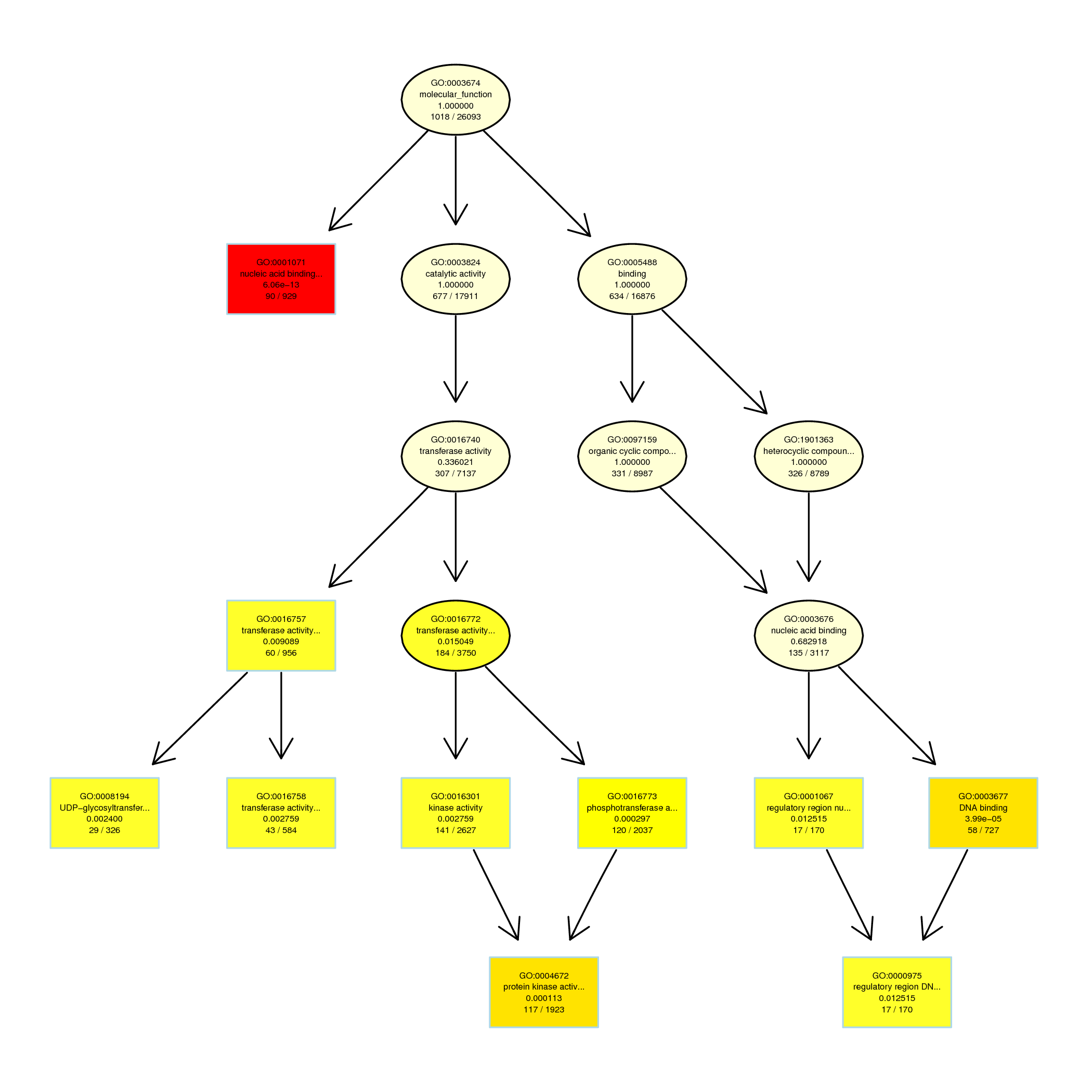
**

**Figure S8: Molecular functions based enriched GO term in CK vs T. Red to yellow color exhibiting the high to low enrichment DEGs in each GO term.**
